# Standardised Nomenclature, Abbreviations, and Units for the Study of Bone Marrow Adiposity: Report of the Nomenclature Working Group of the International Bone Marrow Adiposity Society

**DOI:** 10.3389/fendo.2019.00923

**Published:** 2020-01-24

**Authors:** Nathalie Bravenboer, Miriam A. Bredella, Christophe Chauveau, Alessandro Corsi, Eleni Douni, William F. Ferris, Mara Riminucci, Pamela G. Robey, Shanti Rojas-Sutterlin, Clifford Rosen, Tim J. Schulz, William P. Cawthorn

**Affiliations:** ^1^Department of Clinical Chemistry, Amsterdam Movement Sciences, Amsterdam University Medical Center, Vrije Universiteit, Amsterdam, Netherlands; ^2^Department of Radiology, Massachusetts General Hospital and Harvard Medical School, Boston, MA, United States; ^3^Univ. Littoral Côte d'Opale, Boulogne-sur-Mer, France; ^4^Univ. Lille, Lille, France; ^5^CHU Lille, Lille, France; ^6^Physiopathologie des Maladies Osseuses Inflammatoires, Boulogne-sur-Mer, France; ^7^Department of Molecular Medicine, Sapienza University of Rome, Rome, Italy; ^8^Biological Sciences Research Center “Alexander Fleming”, Athens, Greece; ^9^Department of Biotechnology, Agricultural University of Athens, Athens, Greece; ^10^Division of Endocrinology, Department of Medicine, Faculty of Medicine and Health Sciences, Stellenbosch University, Cape Town, South Africa; ^11^Skeletal Biology Section, NIDCR, NIH, DHHS, Bethesda, MD, United States; ^12^Institute for Research in Immunology and Cancer, Université de Montréal, Montreal, QC, Canada; ^13^Maine Medical Research Center Institute, Scarborough, ME, United States; ^14^German Institute of Human Nutrition Potsdam-Rehbrücke, Nuthetal, Germany; ^15^German Center for Diabetes Research (DZD), München, Germany; ^16^BHF Centre for Cardiovascular Science, The Queen's Medical Research Institute, University of Edinburgh, Edinburgh, United Kingdom

**Keywords:** nomenclature, bone marrow adiposity, bone marrow adipose tissue, bone marrow adipocyte, skeletal stem cells, histomorphometry, MRI, computed tomography

## Abstract

Research into bone marrow adiposity (BMA) has expanded greatly since the late 1990s, leading to development of new methods for the study of bone marrow adipocytes. Simultaneously, research fields interested in BMA have diversified substantially. This increasing interest is revealing fundamental new knowledge of BMA; however, it has also led to a highly variable nomenclature that makes it difficult to interpret and compare results from different studies. A consensus on BMA nomenclature has therefore become indispensable. This article addresses this critical need for standardised terminology and consistent reporting of parameters related to BMA research. The International Bone Marrow Adiposity Society (BMAS) was formed in 2017 to consolidate the growing scientific community interested in BMA. To address the BMA nomenclature challenge, BMAS members from diverse fields established a working group (WG). Based on their broad expertise, the WG first reviewed the existing, unsystematic nomenclature and identified terms, and concepts requiring further discussion. They thereby identified and defined 8 broad concepts and methods central to BMA research. Notably, these had been described using 519 unique combinations of term, abbreviation and unit, many of which were overlapping or redundant. On this foundation a second consensus was reached, with each term classified as “*to use*” or “*not to use*.” As a result, the WG reached a consensus to craft recommendations for 26 terms related to concepts and methods in BMA research. This was approved by the Scientific Board and Executive Board of BMAS and is the basis for the present recommendations for a formal BMA nomenclature. As an example, several terms or abbreviations have been used to represent “bone marrow adipocytes,” including *BMAds, BM-As*, and *BMAs*. The WG decided that *BMA* should refer to “bone marrow adiposity”; that *BM-A* is too similar to *BMA*; and noted that “Ad” has previously been recommended to refer to adipocytes. Thus, it was recommended to use *BMAds* to represent *bone marrow adipocytes*. In conclusion, the standard nomenclature proposed in this article should be followed for all communications of results related to BMA. This will allow for better interactions both inside and outside of this emerging scientific community.

## Introduction

**Figure 1:**
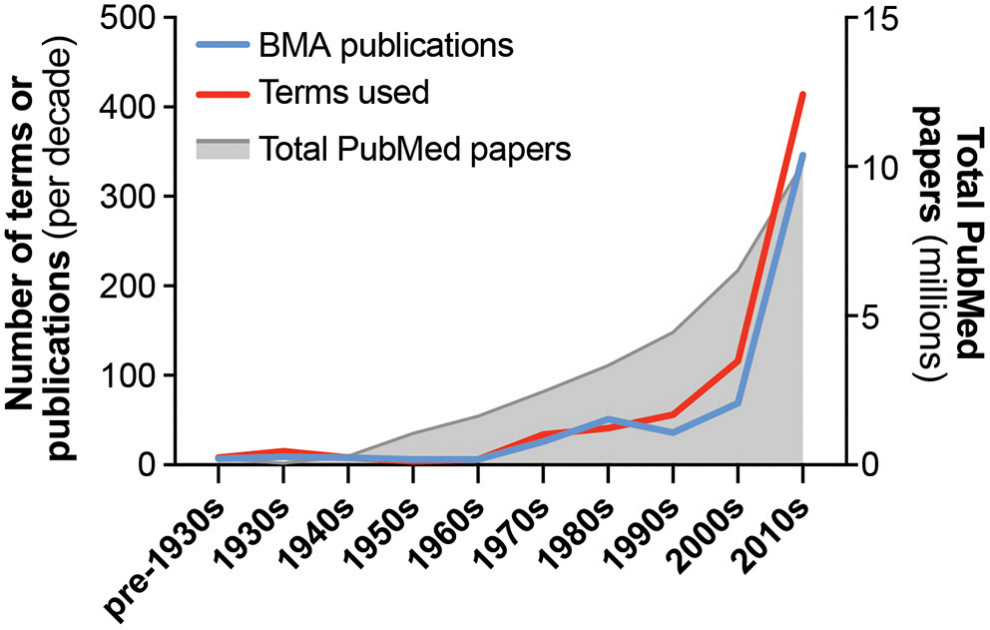
Growth in publications and terminology related to bone marrow adiposity. The number of publications and unique terms relevant to the study of bone marrow adiposity (BMA) are shown, arranged by decade of publication. Also shown is the total number of papers indexed in PubMed for each time period. Publications relevant to BMA were identified through a systematic search of PubMed using the following terms: “marrow[Title] AND (fat[Title/Abstract] OR adipose[Title/Abstract] OR adipocyte[Title/Abstract] OR adiposity[Title/Abstract])”; publication dates up to 31/07/2019 were included. Because earlier studies (e.g., pre-1950) are often not indexed in PubMed, many of these papers were added manually based on our existing knowledge of the literature. Search results were then manually assessed to identify publications relevant to the study of BMA and to exclude results that were not directly relevant. This was necessary because many results related to other subjects, such as fat embolisms of bone marrow or, from 2000 onwards, adipose tissue stem cells. This approach identified 568 papers relevant to BMA nomenclature. By reading these papers, we distinguished the full range of terms that have been used to report concepts and/or measurements related to BMA research. If terms were associated with an abbreviation and/or unit of measurement, these were also recorded. Together, this generated a list of 519 unique combinations of term, abbreviation and unit; if two papers used the same term (e.g., bone marrow adipocyte) but with different abbreviations or units (e.g., BMAd vs. BM-AD), then these were counted as two unique terms. The number of papers and unique terms used per decade are shown. Key concepts and methods are described in Table 1, and terms related to these are presented in Tables 2–9. Further details are provided in Supplementary Tables 1–8.

Bone marrow adiposity (BMA) is the phenomenon of fat storage within the bone marrow (BM). Several different cell types within the skeleton are capable of lipid uptake, including haematopoietic stem cells and osteoblasts (1–3). However, BM adipocytes are the principal cell type responsible for this BM fat storage. Studies relating to BMA have been published since at least the mid-nineteenth century, yet for much of the twentieth century there was relatively little research in this field. However, the past 20 years have seen a resurgence of interest in this topic: even as publication rates have grown across all fields, publications relating to BMA are increasing at an even greater rate (Figure 1). While the earliest studies of BM adipocytes focused on their roles in haematopoiesis, the field has since expanded to include many other disciplines, including skeletal biology, endocrinology and metabolism, stem cells, cancer biology, ageing, biomedical imaging, and beyond. This multidisciplinary nature is one of the strengths of the burgeoning BMA research community; however, it has also contributed to increasing variability in the terminology used in the BMA literature (Figure 1). This is leading to confusion and risks hindering progress in this field. Therefore, there is a need for a standardised nomenclature to facilitate communication between researchers from different fields, and to provide a foundation and consensus for future research relating to BMA.

The challenges for BMA nomenclature were first discussed in 2017 at the Third International Meeting on Bone Marrow Adiposity in Lausanne, Switzerland (4). To address this challenge, members of the International Bone Marrow Adiposity Society (BMAS), representing diverse fields, established a Nomenclature Working Group (WG). The authors of this manuscript represent the Nomenclature WG of BMAS. Our WG has since met several times, including via teleconference and in person, to identify the present state of nomenclature relevant to BMA research and to identify recommended terms, abbreviations and units for a standardised nomenclature. As stated in a previous nomenclature position paper for bone histomorphometry (5), “*Our purpose is not to encourage or discourage the use of abbreviations and symbols but to ensure that the same ones are used by everybody*.”

## Publications and Terminology for the Study of Bone Marrow Adiposity: Historical Perspectives

The existence of adipocytes as a major component of the BM has been noted since at least the 1860s, when Bizozzero and Neumann independently identified BM as the site of blood production (6, 7). In his seminal 1875 book on histology, Ranvier noted that caudal vertebrae of tailed animals are full of fat (8), further confirming that fat is a constituent of normal BM anatomy. Thereafter, references to fat cells, “yellow marrow” and “yellow adipose tissue” within the BM can be found not only in the writings of Neumann (9), but also in contemporary English-language works on pathology and BM anatomy (10, 11). Indeed, Coats noted that the BM is a place “*where normally adipose tissue exists*” (10). Use of the term “yellow marrow” underscored its distinction from the “red marrow” in which haematopoiesis occurs.

Piney's excellent 1922 paper summarised these earlier studies of BM anatomy but used the term “fatty marrow” instead of “yellow marrow” or “yellow adipose tissue” (12). Both “fatty marrow,” “yellow marrow,” and “yellow bone marrow” continued to be used interchangeably in the 1930s, sometimes being combined into the term “yellow fatty marrow” (13–19) or “yellow fat” (15). These and other contemporaneous studies continued to highlight the existence of “fat cells” in histological BM sections (15, 16, 18–21) but the term “adipocyte” is rarely used, and some papers ignore this cellular nature by instead referring to “fat spaces,” (22). Still other studies mention BM fat cells without referring to “fatty marrow” or “yellow marrow” (20, 21), and vice versa (17). Thus, in the 1930s it seems that BM adipocytes were not yet considered as an integrated adipose tissue.

In the latter half of the 1930s and the early 1940s, the term “bone marrow fat” begins to appear, often used alongside “fatty marrow” and “yellow marrow” (19, 23). The term “red marrow fat” was also used in contrast to “yellow marrow fat” (23); this demonstrates recognition that adipocytes also exist in the red marrow, and that these may have different properties to those within the yellow marrow. Hilditch and Murti further noted that the “bone marrow fat” of oxen shares properties of perinephric adipose tissue (23), presaging later suggestions that adipocytes within BM might constitute a *bona fide* adipose tissue (24). Nevertheless, the notion of marrow fat as an adipose tissue was not reiterated until the mid-1950s when Evans et al. suggested that marrow fat is essentially a fat depot, based on its similar lipid composition to perinephric adipose tissue (24). This period also sees the first use of more-quantitative histomorphometric analyses (25, 26), although it is not until the late 1980s that this would become widespread.

From the mid-1950s to mid-1960s the use of “marrow fat” became far more prevalent (24, 27–30), largely replacing “yellow marrow” as the term of choice. One notable exception is the 1967 study by Zakaria and Shafrir (31), who introduced the acronym “YBM” to refer to yellow bone marrow. They showed that, like white adipose tissue (WAT), YBM is capable of uptake and esterification of glucose and free fatty acids, as well as lipolysis for fatty acid release. Based on this, they concluded that YBM could be considered an adipose tissue. This period also includes one of the earliest uses of “marrow adiposity” (32), a term that has become increasingly prevalent in the past two decades (Table 2).

The 1970s saw increased interest in BMA, with notable growth in both the number of publications and the range of terms used therein (Figure 1). The terms “bone marrow adipose tissue” and “bone marrow adipocyte” first appear in the mid-1970s (33, 34), with another obvious shift being the increasing use of “marrow adipose cells,” “marrow adipocytes,” “marrow adipose tissue,” or “bone marrow adipose tissue” (33–45). This may reflect the growing recognition of BM adipocytes, collectively, as an integrated adipose tissue. However, many contemporary papers continued using “fatty marrow,” “yellow marrow,” and “marrow fat” (46–51), and in some cases a mixture of all of these terms can be found within a single paper (52). Thus, the increased study of BMA did not coincide with any consensus or standardisation of the nomenclature used. The late 1970s also saw the use of terms to specify anatomical location, such as “femoral” or “vertebral adipose cell” (40), or “proximal” and “distal” to describe marrow fat and red marrow (51). As we discuss herein in the section on Subtypes of Bone Marrow Adipocytes, terms addressing the site-specific properties of BM adipocytes are an important aspect of the standardised nomenclature for BMA research (Tables 1, 6).

**Table 1:** Key concepts and methods in the field of bone marrow adiposity.

**Concept or method**	**Definition**	**Recommended terms and abbreviations**	**Further details**
**Bone marrow adiposity**	The phenomenon of fat storage within the bone marrow (BM), primarily within BM adipocytes.	• Bone marrow adiposity (BM adiposity; BMA) • Yellow marrow; red marrow; fatty marrow (*Not abbreviated*)	**Table 2**
**Bone marrow adipocytes**	The cells within the BM whose primary function is lipid storage.	• Bone marrow adipocyte (BM adipocyte; BMAd)	**Table 3**
**Bone marrow adipose tissue**	The functionally integrated tissue formed collectively by BM adipocytes	• Bone marrow adipose tissue (BMAT) • Bone marrow fat (BM fat)	**Table 4**
**Site-specific differences (BMAd subtypes)**	The concept that BM adipocytes have distinct morphological, molecular and functional characteristics, depending on their skeletal location. This may also apply to BMAd progenitors.	• Constitutive or Regulated BMAT or BMAd (cBMAT, cBMAd; rBMAT, rBMAd) • *Use “distal” or “proximal” to denote BMAT or BMAd location within long bones; and bone-specific terms to denote skeletal site (e.g., femoral, tibial, vertebral, etc…)*	**Table 5**
**Stem and progenitor cells**	The self-renewing, multipotent progenitor cells within the BM that give rise to adipocytes, osteoblasts, chondrocytes, and fibroblasts.	• Skeletal stem cell (SSC) • Bone marrow stromal cell (BMSC) • *Do not refer to bone marrow/mesenchymal “stem” cells (this is a misnomer)*	**Table 6**
**Histo-morphometry**	Use of histomorphometry to quantify bone marrow adiposity, either on a per-adipocyte level (e.g., *Ad.Ar, Ad.Dm, Ad.Pm*)* or a whole-tissue level *(e.g., adipocyte density, adipose area, adipose volume)*.	• Adipocyte area (Ad.Ar, μm^2^) • Adipocyte diameter (Ad.Dm, μm) • Adipocyte perimeter (Ad.Pm, μm) • Adipocyte density *or* Adipocyte Number* (N.Ad relative to Ma.Ar, Ma.V, T.Ar or TV) • Adipose area^*^ (Ad.Ar relative to Ma.Ar, T.Ar; report as %) • Adipose volume^*^ (Ad.V relative to Ma.V or TV; report as %)	**Table 7**
**Magnetic resonance imaging or spectroscopy (MRI/MRS)**	Use of proton-based MRI/MRS to quantify the proportion or absolute amount of fat within the BM. Typically *in vivo*, volumetric measurements.	• Fat fraction (FF, %) • Bone marrow fat fraction (BMFF, %) • Proton density fat fraction (PDFF, %)	**Table 8**
**Computed tomography**	Use of CT to estimate BM fat fraction, or μCT to measure BMAT volume or BMAd density. Typically done *ex vivo* using contrast agents but can also be done *in vivo*.	• FF (%) or BMFF (%) • Adipose area per marrow area^*^(Ad.Ar/Ma.Ar, %) • Adipose volume per marrow volume^*^ (Ad.V/Ma.V, %) • Adipocyte density^*^ (N.Ad/Ma.V or N.Ad/M.Ar)	**Table 9**

These concepts and methods were identified through a systematic literature search, as described in the legend to Figure 1. The recommendations include 26 unique terms and 35 abbreviations. Abbreviations used in this table (^*^) are defined as follows: N.Ad, adipocyte number; Ad.Ar, total adipocyte area; Ad.V, total adipocyte volume; Ma.Ar, marrow area; Ma.V, marrow volume; T.Ar, tissue area; TV, tissue volume. Further details for each concept/method are provided in the indicated tables and in the main text.

**Table 2:** Summary of terms and abbreviations that have been used to refer to bone marrow adiposity, yellow marrow, fatty marrow, or red marrow.

**Term**	**Abbreviation**	Year first used^*^	No. of papers using this term^*^	**Recommended term(s) and abbreviation(s)**
**BONE MARROW ADIPOSITY**				
Marrow adiposity	–	1967	119	Bone marrow adiposity (BM adiposity, BMA)
Bone marrow adiposity	–	2002	78	
	BM adiposity	2011	12	
	BMA	2013	9	
	BMAT	2017	1	
Bone adiposity	–	2009	4	
**YELLOW MARROW**				
Yellow marrow	–	1883	109	Yellow marrow (*No abbreviation)*
	YM	2010	1	
Yellow bone marrow	–	1936	23	
	YBM	1967	2	
	Yellow BM	1996	2	
	YM	2019	1	
Yellow fatty marrow; yellow fatty bone marrow; yellow fat marrow	–	1933	7	
**FATTY MARROW**				
Fatty marrow	–	1922	82	Fatty marrow (*No abbreviation*)
Fatty bone marrow	–	1990	7	
	Fatty BM	2008	1	
Fatty yellow marrow	–	2013	7	
**RED MARROW**				
Red marrow	–	1883	122	Red marrow *(No abbreviation)*
	RM	2010	2	
Red marrow (active marrow)	–	2003	1	
Red bone marrow	–	1922	21	
	Red BM	1996	2	
	RM	2019	1	
	RBM	2015	2	
h(a)em(at)opoietic marrow	–	1936	24	
**OTHER TERMS (SEE** **SUPPLEMENTARY TABLE 1****)**				

Relevant terms and abbreviations were identified through a systematic search of the literature relating to bone marrow adiposity. Within these search results, the total number of papers using each term/abbreviation combination is indicated, along with the year that these were first used; asterisks (^*^) are shown to emphasise that other uses may exist. For most terms, no abbreviation is used (–). Based on this and other considerations (described in the main text), we recommend use of the term bone marrow adiposity (abbreviated as “BM adiposity” or “BMA”). Use of the terms “yellow marrow,” “fatty marrow” or “red marrow” is also acceptable, given the long-term use of these terms in the BMA literature. Red marrow is in some cases described as hematopoietic marrow, hemopoietic marrow, haematopoietic marrow or haemopoietic marrow; for simplicity, we have grouped together papers using these spelling variants. Terms specific to measurements of bone marrow adiposity, such as “fat fraction” or “fat content,” have not been included and are shown instead in Tables 6–8. Additional terms and abbreviations, as well as references for papers using each combination, are provided in Supplementary Table 1.

**Table 3:** Summary of terms and abbreviations used to refer to bone marrow adipocytes.

**Term**	**Abbreviation**	Year first used^*^	No. of papers using this term^*^	**Recommended term(s) and abbreviation(s)**
**BONE MARROW ADIPOCYTE**				
Adipocyte	–	1981	39	Bone marrow adipocyte (BM adipocyte, BMAd)
	AC	2018	1	
Adipose cell	–	1971	16	
	Adip	2005	1	
Bone adipocyte	–	1981	6	
Marrow adipocyte	–	1978	105	
Marrow adipose cell	–	1971	8	
Bone marrow adipocyte	–	1976	100	
	BM adipocyte	1995	30	
	BMA	2016	17	
	BMAd	2019	2	
	MAT adipocyte	2015	6	
	BMAT adipocyte	2018	3	
	BM-A	2013	2	
	BM-AD	2018	2	
	Marrow AC	2016	1	
Bone marrow adipose cell	–	1974	3	
Marrow-associated adipose cell	–	1974	1	
Medullary adipocyte	–	2010	1	
Skeletal adipocyte	–	2015	1	
Yellow adipocyte	–	2019	1	
**BONE MARROW FAT CELL**				
Fat cell	–	1893	48	Don't use
	FC	1935	1	
Fatty cell	–	1985	3	
Marrow fat cell	–	1931	14	
Bone marrow fat cell	–	1966	10	
	BM fat cell	1998	1	
Fat-containing cell	–	1980	2	
	FCC	1983	1	
***OTHER TERMS (SEE****SUPPLEMENTARY TABLE 2****)***				

Data are presented as described for Table 2; asterisks (^*^) are shown in the column headers to emphasise that other uses may exist.

*Based on this and other considerations (described in the main text), we recommend use of the term bone marrow adipocyte, abbreviated as BM adipocyte or BMAd. Terms related to measurements of bone marrow adipocytes, such as “adipocyte density,” have not been included and are shown instead in Tables 6–8. Additional terms and abbreviations, as well as references for papers using each combination, are provided in Supplementary Table 2*.

**Table 4:** Summary of terms and abbreviations used to refer to bone marrow adipose tissue, yellow adipose tissue, or marrow fat.

**Term**	**Abbreviation**	Year first used^*^	No. of papers using this term^*^	**Recommended term(s) and abbreviation(s)**
**BONE MARROW ADIPOSE TISSUE**				
Adipose tissue	–	1883	17	Bone marrow adipose tissue (BMAT)
Adipose tissue from bone marrow	–	1999	1	
Adipose marrow	–	1991	4	
Adipose marrow tissue	–	2017	1	
Marrow adipose	–	1971	2	
Marrow adipose tissue	–	1971	31	
	MAT	2012	56	
Marrow-associated adipose tissue	–	1974	1	
Bone marrow adipose	–	1979	5	
bone marrow adipose tissue	–	1974	34	
	BMAT	2007	49	
	BM adipose tissue	1988	6	
	MAT	2014	15	
	BM-AT	2019	1	
**MARROW FAT**				
Marrow fat	–	1954	101	Marrow fat or Bone marrow fat (BM fat)
	MF	2008	3	
Bone marrow fat	–	1936	80	
	BM fat	2002	3	
	BMF	2007	11	
	MF	2014	1	
Medullary fat	–	1994	2	
Bone fat	–	2010	1	
**YELLOW ADIPOSE TISSUE**				
Yellow adipose tissue	–	1883	2	Don't use
	YAT	2011	4	
Yellow adipose	–	2016	1	
Yellow fat	–	1933	2	
Yellow adipose marrow	–	2018	1	
Fatty tissue	–	1922	1	
Fatty yellow adipose tissue	–	2019	1	
Bone marrow fatty tissue	–	1993	1	

Data are presented as described for Table 2; asterisks (^*^) are shown in the column headers to emphasise that other uses may exist. Based on this and other considerations (described in the main text), we recommend use of the term bone marrow adipose tissue, abbreviated as BMAT. Use of “marrow fat” or “bone marrow fat (BM fat)” is also acceptable, given the long-standing use of these terms in the BMA literature. References for papers using each combination of term and abbreviation are provided in Supplementary Table 3.

**Table 5:** Summary of terms and abbreviations used to refer to different subtypes of BMAT and BMAds.

**Term used**	**Abbreviation**	Year first used^*^	No. of papers using this term^*^	**Recommended term(s) and abbreviation(s)**
**CONSTITUTIVE OR REGULATED BMAT AND BMAds**				
Constitutive marrow adipose tissue	cMAT	2014	26	**Use of cBMAT, cBMAd, rBMAT, and rBMAd is acceptable** *(But when discussing subtypes, it is preferable to refer to skeletal location of the BMAT and BMAds)*
	Constitutive MAT	2017	1	
Constitutive bone marrow adipose tissue	cBMAT	2017	7	
	Constitutive BMAT	2017	2	
Constitutive bone marrow adipocyte	cMAT adipocyte	2015	6	
	cBMA	2016	7	
Regulated marrow adipose tissue	rMAT adipocyte	2014	26	
	Regulated MAT	2017	1	
Regulated bone marrow adipose tissue	rBMAT	2017	7	
	Regulated BMAT	2017	2	
Regulated bone marrow adipocyte	rMAT adipocyte	2015	6	
	rBMA	2016	8	
	–	2017	1	
**DISTAL OR PROXIMAL BMAT AND BMAds**				
Distal marrow adipose tissue	distal MAT	2016	1	**Use “distal” or “proximal” to refer to BMAT/BMAd location within long bones**
Distal marrow fat	–	1979	1	
Distal tibia marrow adipose tissue	dMAT	2017	1	
Proximal marrow adipose tissue	Proximal MAT	2016	1	
Proximal marrow fat	–	1979	1	
Proximal tibia marrow adipose tissue	pMAT	2017	1	
**BONE-SPECIFIC TERMS**				
Femoral adipose cell	–	1977	1	**When required, use bone-specific terms to indicate location of BMAT or BMAds** *(see text for further details)*
Femur marrow fat	FMF	1984	1	
Tibial adipose bone marrow	–	1985	1	
Vertebrae adipose cell	–	1977	1	
Vertebral fat	–	1989	1	
Intravertebral bone marrow fat	–	2002	1	
Vertebral bone marrow fat	–	2012	1	
	VMB fat	2017	1	
Vertebral BMAT	vBMAT	2018	1	
Vertebral marrow fat	–	2012	1	
Vertebral MAT	vMAT	2018	1	
**BMAT WITHIN RED OR YELLOW MARROW**				
Red marrow fat	–	1940	2	**Don't use**
Red bone marrow fat	–	2001	2	
	RBM fat	2019	1	
Red marrow adipose tissue	rMAT	2018	2	
Yellow marrow adipose tissue	yMAT	2018	2	

Data are presented as described for Table 2; asterisks (^*^) are shown in the column headers to emphasise that other uses may exist. When necessary, we recommend indicating site-specific differences in BMAT/BMAd subtypes by referring to the skeletal site (e.g., “femoral,” “tibial,” “vertebral”) and the location within these bones (e.g., “proximal” or “distal”). Use of “constitutive” or “regulated” is also acceptable, with the recommended acronyms being cBMAT, cBMAd, rBMAT, and rBMAd, consistent with the recommended nomenclature for bone marrow adipocytes (BMAd) and bone marrow adipose tissue (BMAT). Further considerations are provided in the main text. References for papers using each combination of term and abbreviation are provided in Supplementary Table 4.

**Table 6:** Summary of terms and abbreviations used to refer to progenitors for BMAds.

**Abbreviation**	**Term used**	Year first used^*^	No. of papers using this term^*^	**Recommended term(s) and abbreviation(s)**
**SSC**	Skeletal stem cell (SSC)	2004	12	**Skeletal stem cell (SSC)**
	Skeletal stem cell (*no abbreviation)*	2004	11	
	Mouse skeletal stem cell (mSSC)	2015	1	
	human skeletal stem cell (hSSC)	2018	1	
**BMSC**	Bone marrow stromal cell	2004	20	**Use BMSC to refer to bone marrow stromal cells** *(see text for further considerations)*
	Bone marrow mesenchymal stem cell	2013	5	
	Bone marrow mesenchymal stem cell	2012	1	
	Bone marrow stromal cell (mBMSC)^**^	2015	1	
	Bone mesenchymal stem cell	2016	1	
	Bone-derived marrow mesenchymal stem cell	2017	1	
	Bone marrow stromal cell (BM stromal cell)	2017	1	
	Bone marrow mesenchymal cell	2018	1	
	Bone marrow mesenchymal stromal cell	2018	1	
	Not defined	2018	1	
	Bone marrow mesenchymal stem/stromal cell	2019	1	
**MSC**	Mesenchymal stem cell	2003	59	**Don't use**
	Mesenchymal stromal cell	2012	10	
	Abbreviation not defined	2015	5	
	Human mesenchymal stem cell (hMSC)	2004	4	
	Bone marrow mesenchymal stromal cell	2014	4	
	Mesenchymal stem/stromal cell	2016	4	
	Marrow stromal cell	2003	3	
	Bone marrow mesenchymal stem cell	2015	3	
	Bone marrow stromal cell	2004	2	
	Human bone marrow stromal cell (hMSC)	2004	1	
	Bone marrow mesenchymal stem cell	2012	1	
	Mesenchymal progenitor	2016	1	
	Bone marrow-derived mesenchymal stem cell	2018	1	
	Bone mesenchymal stem cell	2018	1	
**BM-MSC**	Bone marrow mesenchymal stem cell	2013	5	**Don't use**
	Bone marrow mesenchymal stromal cell	2018	2	
	Bone marrow MSC	2017	1	
	Bone marrow skeletal stem cell^‡^	2018	1	
	Bone marrow stromal stem cell^‡^	2019	1	
*No abbreviation used (see Supplementary Table 5)*				

Data are presented as described for Table 2; asterisks (^*^) are shown in the column headers to emphasise that other uses may exist. Terms are organised by abbreviation used, and then by frequency and date of use. Further considerations are provided in the main text. Terms used without an abbreviation, and references for papers using each combination of term and abbreviation, are presented in Supplementary Table 5.

** *mBMSC, mouse bone marrow stromal cell*.

‡ *Although we recommend using “skeletal stem cell” and “bone marrow stromal cell,” the former should be abbreviated as “SSC” rather than “BM-MSC”; and the latter should be described as a “stromal cell,” not a “stem cell,” and should be abbreviated as “BMSC,” not as “BM-MSC”*.

The number of BMA-related publications and terms continued to increase in the 1980s (Figure 1), with one notable development being the emergence of new methods for quantitative assessment of BMA. These include magnetic resonance imaging (MRI) (53–57), computed tomography (CT) and dual-energy CT (58–61), and advances in histomorphometric analysis (62–64). Typically, these methods provide readouts of the fraction of BM consisting of fat or adipocytes, as reflected by use of terms such as “fat fraction” (56), “fat content” (57–59, 61, 65), and “adipose tissue fraction” (63). These method-related terms often are associated with units of measurement, although the units used frequently vary between studies. This theme has persisted in more-recent BMA research, with numerous combinations of terms, abbreviations, and units applied to the same measurement (Tables 7–9). Thus, an important goal of our proposed nomenclature is to standardise the terminology used in reporting common measurements in the BMA field.

**Table 7:** Summary of terms, abbreviations, and units that have been used to report histomorphometric measurements of bone marrow adiposity.

**Measurement type**	Year first reported^*^	No. of papers^*^	**Recommended term(s)**	**Recommended abbreviation(s) and unit(s)**
**Adipocyte area** (per cell; mean or median)	2002	28	**Adipocyte area** (mean or median)	Ad.Ar, μm2 *(Showing the frequency distribution of Ad.Ar is recommended)*
**Adipocyte area** (total)	2015	1	**Don't use** *(instead report as % Ma.Ar or % T.Ar)*	
**Adipocyte diameter** (per cell; mean or median)	1989	25	**Adipocyte diameter** (mean or median)	Ad.Dm, μm *(Showing the frequency distribution of Ad.Dm is recommended)*
**Adipocyte perimeter** (per cell; mean or median)	2002	5	**Adipocyte perimeter** (mean or median)	Ad.Pm, μm *(Showing the frequency distribution of Ad.Pm is recommended)*
**Adipocyte perimeter** (total; mean or median)	2015	1	**Don't use** *(instead report average Ad.Pm per cell, as above)*	
**Adipocyte density** (cells per field, area, or volume)	1990	70	**Adipocyte density** or **Adipocyte number**	N.Ad/Ma.Ar, N.Ad/Ma.V, N.Ad /T.Ar, N.Ad /TV. *Units should be clearly stated (e.g., cells/mm2, cells/μm2, etc …)*
**Adipocyte number** (absolute N.Ad without clearly defined referent)	1993	10	**Don't use** *(instead report Adipocyte density or Adipocyte number as N.Ad per referent area or volume, as above)*	
**Adipose area** (per field, marrow area or tissue area)	1950	39	**Adipose area** or **Adipose tissue area**	**Report as adipose area (Ad.Ar) relative to marrow area (Ma.Ar) or tissue area (T.Ar)**. *This must be presented as %*
**Adipose tissue area** (absolute)	2017	1	**Don't use** *(instead report as % Ma.Ar or % T.Ar, as above)*	
**Adipose volume** (per field, marrow volume or tissue volume)	1991	29	**Adipose volume** or **Adipose tissue volume**	**Report as adipose volume (Ad.V) relative to marrow volume (MA.V) or tissue volume (TV)**. *This must be presented as %*.
**Adipose volume** (absolute)	1983	1	**Don't use** *(instead report as % Ma.V or % TV, as above)*	
**Other Measurements** (Supplementary Table 6)			**Don't use**	

Terms are grouped based on the type of measurement reported. For each type of measurement, several different terms, abbreviations and units have been used in the literature; these variants are shown in Supplementary Table 6. For the Year first reported and the Number of papers, asterisks (^*^) are shown to emphasise that other uses may exist. We recommend that measurements of BMAd density are presented as cell number relative to the area or volume of the marrow or total tissue, and that these are reported as “Adipocyte density”; however for consistency with the published guidelines for bone histomorphometry (5), it is also acceptable to describe this measurement as “Adipocyte number,” provided that a suitable referent region is used. Adipocyte area (Ad.Ar; μm^2^) or volume (Ad.V; μm^3^) can be reported either for individual adipocytes or for the sum of all adipocytes within the regions analysed. The former should be reported as the mean or median per adipocyte or as a distribution of adipocyte areas or volumes per sample. The latter should be reported as “Adipose area” and presented as % relative to the area or volume of the marrow (Ma) or total tissue (T). This is consistent with the standardised nomenclature for bone histomorphometry (5), and with our recommendations for BMA measurements based on MRI/MRS or CT (Tables 8, **9**). In some instances, it may be necessary to use “Extravascular marrow” area or volume (ExVa.Ma.Ar or ExVa.Ma.V). This and further considerations are discussed in the main text. References for papers using each combination of term, abbreviation and unit are provided in Supplementary Table 6.

**Table 8:** Summary of the most common terms, abbreviations, and units that have been used to report MRI/1H-MRS-based measurements of bone marrow adiposity.

**Term**	**Abbreviations used**	**Units used**	Year first used^*^	No. of papers using this term^*^	**Recommendations for MRI/MRS measurements**
**FAT FRACTION**					
**Fat fraction**	*None;* FF	*n.s.;* %; Lipid/water ratio	1999	27	**Fat fraction (FF), Bone marrow fat fraction (BMFF), proton density fat fraction (PDFF)**. *Values should be reported as %*.
**Marrow fat fraction**	*None;* FF; MFF	*n.s.;* %; Lipid/water ratio	1992	27	
**Bone marrow fat fraction**	*None;* BM fat fraction; BMF; BMFF; FF	*n.s.;* %; Lipid/water ratio	1995	23	
**Proton density fat fraction**	*None;* proton density FF; PDFF; bone marrow PDFF	*n.s.;* %	2014	21	
Signal fat fraction; fat signal fraction	*None;* sFF	*n.s.;* %	2012	7	
Lipid fraction; bone marrow lipid fraction	*None;* LF	%; Lipid/water ratio	1985	4	
**FAT CONTENT**					
Fat content	*None;* FC	*n.s.;* %	1987	15	**Don't use for reporting MR-based measurements of fat fraction**.
Bone marrow fat content	*None;* BMF content; bone marrow FC; FC	*n.s.;* %; Lipid/water ratio	2009	11	
Marrow fat content	*None*	%; % Fat fraction; L	2005	9	
Bone marrow adipose tissue content	MAT content; BMAT content	*n.s.;* %; Lipid/water ratio	2016	3	
**RATIO (FAT/WATER OR LIPID/WATER)**					
Lipid/water ratio	*None;* l/w; LWR	%; Lipid/water ratio	2004	13	**Don't use**
Fat/water ratio; bone marrow fat/water ratio	*None*	%; Lipid/water ratio	2014	1	
Bone marrow fat/water %	*None*	%	2011	1	
**AREA**					
Bone marrow adipose tissue area	BMA	cm^2^	2015	1	**Don't use**. *Instead present relative to marrow area*
Bone marrow adiposity cross-sectional area	CSA	cm^2^	2017	1	
Yellow bone marrow cross-sectional area	CSA	mm^2^	2013	1	
**OTHER TERMS (SEE** **SUPPLEMENTARY TABLE 7****)**					

Terms are grouped into subcategories (bold, capitalised text) based on the wording used. Other data are presented as described for Tables 2–7; for the Year first reported and the Number of papers, asterisks (^*^) are shown to emphasise that other uses may exist. We recommend that MRI/MRS-based readouts be reported as fat fraction, because this is the approach used most commonly in the existing literature. For consistency, fat fraction measurements should be reported as %, rather than as lipid/water ratios. Measurements of BMAT area (Ad.Ar) should be reported as % of marrow area (M.Ar), i.e., Ad.Ar/M.Ar, as recommended for histological or CT-based measurements (Tables 7, 9). Further considerations are discussed in the main text. Additional terms/abbreviations/units, as well as references for papers using each combination, are provided in Supplementary Table 7.

**Table 9:** Summary of terms, abbreviations, and units that have been used to report CT-based measurements of bone marrow adiposity.

**Imaging method**	**Measurement type**	Year first reported^*^	No. of papers^*^	**Recommended term(s)**	**Recommended abbreviation and unit**
***SECT, DECT** or **μCT***(Without contrast agent)**	**Fat fraction** (% or fraction)	1987	8	**Fat fraction; Bone marrow fat fraction**	**FF** (%), **BMFF** (%)
	**Adipose volume** (per marrow volume)	2019	2	**Adipose volume**	**Ad.V/Ma.V** (%)
	**BM density**	1986	6	**Don't use** *(Report as Adipose volume, FF or BMFF, as above)*	
	**Adipose volume** (per tissue volume)	2011	2	**Don't use** *(Report as % Ma.V, as above)*	
***μCT** (Samples stained with osmium tetroxide or other contrast agents)*	**Adipose volume** (per marrow volume)	2012	11	**Adipose volume**	Ad.V/Ma.V (%)
	**Adipocyte density** (per marrow volume)	2014	2	**Adipocyte density** or **Adipocyte number**	N.Ad/Ma.V *(Cell number/unit volume)*
	**Adipocyte area** (Distribution of individual adipocyte areas)	2015	1	**Adipocyte area** (mean or median)	**Ad.Ar**, **μm**^**2**^ *(Show frequency distribution)*m
	**Total adipocyte area** *(per Slice)*	na	na	**Adipose area**	**Ad.Ar/Ma.Ar** (%)
	**Adipocyte volume** *(per slice)*	na	na	**Adipocyte volume** (mean or median)	Ad.V, μm^3^ *(show frequency distribution)*
	**Adipose volume** *(per tissue or total volume)*	2014	12	**Don't use** *(Report as % Ma.V, as above)*	
	**Adipose volume** *(Unclear if per tissue volume or per marrow volume)*	2015	4		
	Adipose volume (absolute)	2015	10		

Data are presented as described for Table 7, except that imaging method is also indicated. For the Year first reported and the Number of papers, asterisks (^*^) are shown to emphasise that other uses may exist. For each type of measurement, several different terms, abbreviations, and units have been used in the literature; these variants are presented in Supplementary Table 8. Most in vivo studies using SECT or DECT report measurements of fat fraction or relative adipose volume, and therefore we recommend reporting these as indicated in the table. Most ex vivo μCT studies assess adipose volume normalised to marrow volume or total tissue volume, although in some cases it is unclear which of these is used for normalisation. Other studies present absolute adipose volume or assess adipocyte density or area. We recommend that μCT-based readouts of adipose volume or area be reported relative to marrow volume (Ma.V) or marrow area (Ma.Ar), and that individual adipocyte areas be reported as the mean or median, or as a frequency distribution. This is consistent with our recommendations for histology-based measurements (Table 7). Further considerations are discussed in the main text. References for papers using each combination of term, abbreviation and unit are provided in Supplementary Table 8.

In the 1990s there was further growth in the number of terms used, despite a slight decrease in publications (Figure 1). This suggests an increasing diversity and continued lack of consensus for BMA nomenclature. Studies using histomorphometry or MRI became increasingly prevalent, with proton magnetic resonance spectroscopy (1H-MRS) also emerging as a powerful tool for BMA quantification (Tables 7, 8). The 1990s also saw an increased focus on progenitors for BM adipocytes, exemplified by references to “bone marrow stromal cells” (Table 6).

The numbers of BMA-related papers and terminology further increased in the 2000s, and since 2010 this growth has been even more substantial (Figure 1). The abbreviation BMAT, for “bone marrow adipose tissue,” first appears in 2007 (66), preceding the first use of MAT (“marrow adipose tissue”) in 2012 (67); each of these abbreviations is now widespread in the BMA literature (Table 3). Similarly, “bone marrow adiposity” first appears in 2002 but has become increasingly prevalent ever since (68) (Table 2). The past two decades have also witnessed the development of new methods for BMA analysis, including μCT of osmium tetroxide-stained bones (69, 70) and advances in MRI/MRS-based quantitation (71). These developments therefore have led to the introduction of additional new terms related to such methods (Tables 7–9). Many other notable terms have also emerged in the BMA literature during this period, such as SSC (for “skeletal stem cell”) (72) (Table 6), and use of “regulated” and “constitutive” to distinguish distinct BMAT subtypes (73) (Table 5). Given the vast number of terms used since 2000, it is not possible to succinctly summarise all of the key developments in these paragraphs. Therefore, readers should consult Tables 1–9, and further data in the Supplement, for a full overview of the nomenclature used in the BMA literature to date.

## Key Concepts and Goals for a Standardised Nomenclature

This historical overview is based on our systematic search of PubMed (Figure 1) and our knowledge of other less-accessible papers. Thus, references to the “first use” of a term apply only to this extensive body of BMA-related literature; because some relevant publications may have been missed, some terms may have even earlier uses. Nevertheless, these historical perspectives on the BMA literature provide an essential foundation on which to establish a standardised nomenclature for studies relevant to BMA. The two major benefits of this literature review are as follows.

Firstly, we show that the existing BMA literature uses a highly diverse, heterogeneous terminology to report concepts and measurements relevant to BMA. As shown in Figure 1, the rate of publications within the BMA literature has increased dramatically in the past decade, reflecting the growing interest in this topic; however, the number of unique terms is growing even more quickly (Figure 1). Thus, since the 1990s, there has been an increasing diversity and lack of consensus for nomenclature relating to BMA research. Indeed, new terms continue to be proposed to this day (74). It is likely that research into BMA will continue expanding, and therefore it is essential to adopt a standardised nomenclature to provide a foundation and consensus for this growing field.

Secondly, reviewing the history of BMA research has identified key concepts and methods relevant to studies of BMA (Table 1). A standardised nomenclature must therefore incorporate terms, abbreviations and units related to these concepts and methods.

The following sections provide further discussion of the nomenclature for each of these, concluding with recommendations for the terms, abbreviations and units to be used in future reports of BMA research.

## Nomenclature Recommendations

### Bone Marrow Adiposity

We define bone marrow adiposity as “The phenomenon of fat storage within the BM, primarily within BM adipocytes.” This can be considered an overarching concept for the field: it is the central theme that links several diverse disciplines, including haematology, bone biology, metabolism, endocrinology, stem cells, developmental biology, oncology, gerontology, and beyond. Given the centrality of this concept, we think it is important to provide both a clear definition and a standardised abbreviation for use in future studies. The abbreviation “BM adiposity” is recommended because “BM” is already used across the biomedical literature to refer to bone marrow, and therefore “BM adiposity” should be widely understandable. However, we also recommend the abbreviation “BMA” for two reasons: firstly, this abbreviation for “bone marrow adiposity” has become increasingly common in the literature since its first use in 2013 (Table 2); and, secondly, “BMA” is now recognised for this meaning through its use in the name BMAS (the International Bone Marrow Adiposity Society) and in the names of the five international meetings devoted to this topic (BMA2015–BMA2019) (4, 75, 76). It is important to note that BMA has also been used as an acronym for “bone marrow aspiration” (77) and “bone marrow aspirate” (78); however, the increasing use and recognition of BMA to refer to bone marrow adiposity should minimise any confusion with these other uses.

In considering the concept of BMA, it is essential to also highlight the terms “yellow marrow,” “fatty marrow,” and “red marrow.” Each of these refers to concepts intimately related to BMA and has longstanding and widespread use in the field (Table 2). Thus, “yellow marrow” and “fatty marrow” are used to refer to more-lipid-laden regions of BM whereas “red marrow” refers to those regions of the BM where adiposity is less prominent and haematopoiesis predominates; each of these terms is also used for macroscopic descriptions of different regions of BM, often in clinical contexts. Although numerous variations of these three terms have been used, “yellow marrow,” “fatty marrow,” and “red marrow” are by far the most common (Table 2 and Supplementary Table 1). Thus, given their widespread use, historical significance and clinical recognition, we recommend continued use of these terms in future studies relevant to BMA.

Further details of these four recommended terms, and the many terms that we recommend to avoid using, are provided in Table 2 and Supplementary Table 1.

### Bone Marrow Adipocytes

A defining feature of BMA is the storage of lipid within bone marrow adipocytes. We define these cells as a population of *bona fide* adipocytes, that is, a cell type whose main functional and morphological characteristic is the storage and metabolism of lipids in a single large or several smaller triglyceride-filled vacuoles. This distinguishes adipocytes from other cell types, such as hepatocytes and myofibers, that in principle are also able to store triglycerides ectopically. In these latter cells, lipid accumulation is thought of as predominantly pathological, presumably due to lipotoxic reactions. In addition, haematopoietic stem cells and osteoblasts are also capable of lipid uptake (1–3). However, unlike these other cell types, mature adipocytes are uniquely equipped for metabolising and storing triglycerides and intermediates of lipid metabolism and feature a higher level of resistance to lipotoxicity. We would therefore also recommend against use of less-specific or even colloquial terms, including “fatty cell” or “fat cell” in a combination with the bone marrow, as these may refer to any type of lipid-containing cell (Table 3 and Supplementary Table 2).

A number of abbreviations to specifically define bone marrow-resident adipocytes have been used in the recent literature on BMA. Our recommendation is to maintain consistency with the broader context of adipocyte biology and bone histomorphometry, where adipocytes are commonly abbreviated with “Ad” (5). We therefore propose the consistent use of the terms “BMAd” or “BM adipocyte” to designate mature adipocytes within the bone marrow (Table 3 and Supplementary Table 2).

In addition to their ability to store lipids, another important property of BMAds is secretion of bioactive factors. This is reminiscent of both white and brown adipocytes, which release hormones, lipid species, cytokines, and other factors to exert local and systemic effects (79). Collectively, such adipocyte-derived secreted factors are known as “adipokines” and these contribute extensively to the physiological and pathological functions of adipose tissue (79). BMAds are also becoming increasingly recognised for their ability to secrete adipokines and thereby exert paracrine and endocrine functions (80). The two most prominent adipokines are the hormones leptin and adiponectin, which regulate energy homeostasis and have other diverse effects (79). BMAds express and secrete leptin both in primary culture and after *in vitro* differentiation from human bone marrow stromal cells (BMSCs) (81–83). BMAds also express and secrete adiponectin and might influence circulating concentrations of this adipokine (80, 84, 85). This suggests that BMAds might have endocrine functions.

BMAds also secrete many other endocrine and paracrine factors, including RANKL (86–88), DPP-4 (89), and stem cell factor (SCF) (90); cytokines such as interleukin-6 (IL-6), IL-3, IL-8, tumor necrosis factor-α (TNF-α), CXCL1, CXCL2, CXCL12, and MCP-1 (91–95); lipid species, such as free fatty acids (96–98); and RNA molecules within extracellular vesicles (80). Through these factors BMAds are reported to modulate haematopoiesis and skeletal remodelling. A full discussion of these functions is beyond the scope of this position paper, but more details are available in several recent reviews (80, 99, 100).

Relating to lipid storage, an unresolved question concerns BM adipocytes' unilocular vs. multilocular nature. These properties are traditionally linked to white and brown adipocyte identity, respectively (79, 101). Some studies suggest that BMAds with a brown adipocyte-like phenotype can occur in the bone marrow cavity (102), and brown adipocyte-like phenotypes can be induced *in vitro* using cell culture models of BMAds, for example after overexpression of FoxC2 (103) or SIRT1 (103). However, recent data indicate that *in vitro* cell models do not reliably recapitulate the properties of BMAds *in vivo* (74). Indeed, microarrays show that UCP1 transcripts are not enriched in whole BM of mice or humans (104, 105), nor is *Ucp1* expression greater in BMAds vs. white adipocytes of mice (106). Recent work using lineage tracing and genetic models has also demonstrated that BMAds do not express *Ucp1* during development or after adrenergic stimulation in mice (107). Similarly, primary BMAd progenitors have very limited, if any, brown adipogenic potential (89), and BMAds *in vivo* do not undergo cold-induced glucose uptake (108). However, multilocular BMAds do exist and account for around 5% of all BMAds in the long bones of mice (107). Thus, while it seems that BM adipocytes are distinct from brown adipocytes, it is unlikely that this multilocularity is indeed equated to brown adipocyte-like functions of BMAds. This also pertains to BMAd-intrinsic ability for rapid lipid mobilization in response to adrenergic stimulation or other physiological stimuli known to recruit brown adipocytes for their main function, which is thermogenesis. These remain open issues to be assessed in greater detail. Future authors may therefore opt to add further attributes to the term “bone marrow adipocyte” to better define parameters such as number of vacuoles (locularity), and also to reference anatomical localisation or metabolic characteristics. This relates to the recent discussion on distinct types of BMAds, regulated and constitutive, which will be discussed in the section on Subtypes of Bone Marrow Adipocytes.

### Bone Marrow Adipose Tissue

One area of debate is whether BMAds are simply a subpopulation of BM cells that constitute a part of BM as a tissue, or whether, collectively, BMAds act as an integrated adipose tissue. References to “adipose tissue” within the BM can be found as early as 1883 (10), and studies from the 1940s and 1950s also proposed “yellow marrow” or “marrow fat” to be an adipose tissue, based on its similar lipid composition with WAT depots (23, 24). In 1967, Zakaria and Shafrir studied explants of “yellow bone marrow” (YBM), concluding: “*The experiments demonstrate the capacity of YBM to synthesize fatty acids and glycerol from glucose, to take up and esterify long-chain FFA (free fatty acids) from an external medium and to release FFA under hormonal stimulation. All these activities are typical of adipose tissue function. Thus, the YBM seems to represent a metabolically active variety of fat store, similar to depots in other anatomical sites, but presumably with a specialized local importance”* (31). These studies support the concept that BMAds, collectively, form an integrated adipose tissue.

One caveat, discussed further in the section on Subtypes of Bone Marrow Adipocytes, is that BMAd characteristics vary depending on skeletal site. The studies described above focussed on the yellow marrow, in which BMAds form a contiguous unit that is morphologically similar to white adipose tissue (109). However, BMAds also exist interspersed among the red marrow, where they do not form a spatially contiguous grouping; it is less clear whether these cells, designated “regulated” BMAds, can be considered as an adipose tissue.

This issue was debated extensively among the members of the Nomenclature WG. After much discussion, we concluded that it is appropriate to refer to BMAds as an adipose tissue, even for those adipocytes interspersed among the red marrow. This decision is based on two key points. Firstly, even in white adipose tissue, adipocytes comprise <25% of the total cell population (110). Thus, the fact that BMAds do not predominate in the red marrow does not preclude these from being considered as an adipose tissue. Secondly, “tissue” has been defined as “*an aggregation of similarly specialized cells united in the performance of a particular function”* (5); hence, possessing a common function is more important than the physical grouping of the cells. Although the roles of BMAds are still being elucidated, it is clear that these cells work together to perform common functions (109), and therefore they can be considered as an integrated adipose tissue.

A second point of debate regards how to abbreviate “bone marrow adipose tissue.” By far the most common abbreviations for this are “MAT” and “BMAT” (Table 4). One benefit of “MAT” is that this is more consistent with other abbreviations in the adipose field, such as “WAT” and “BAT.” One downside to “BMAT” is that this also been used to refer to a “bone marrow aspirate and trephine” biopsy (111); however, there are several benefits to the use of “BMAT” to abbreviate “bone marrow adipose tissue.” Firstly, by using “BM,” “BMAT” refers unambiguously to the BM and is consistent with other abbreviations recommended in this standardised nomenclature (e.g., BMA, BMAd, BMSC, BMFF). Secondly, in the BMA literature the use of “BMAT” precedes use of “MAT” by 5 years, and therefore there is a historical priority for “BMAT” (Table 4). Finally, use of “BMAT” has been recommended in a previous editorial (112), which provides further rationale for its continued use. In summary, we recommend the term “bone marrow adipose tissue” and the abbreviation “BMAT.” This can be defined as a collection of BMAds, which may be clustered together or interspersed among the haematopoietic marrow, that work together to perform common functions.

Two other terms are also recommended. Despite the longstanding references to adipose tissue within the BM, it has historically been more common to refer to “marrow fat” or “bone marrow fat” (Table 4). These terms continue to be used to this day, often in clinical reports and/or to reflect gross measurements of BMA (Supplementary Table 3). Considering the historical prominence and continued use of these terms, we have included “marrow fat” and “bone marrow fat (BM fat)” in the standardised BMA nomenclature. However, when discussing the formation and function of BMAds as a collective, integrated tissue, use of “bone marrow adipose tissue (BMAT)” should be given preference.

Further details of these recommendations, and the many terms that we recommend to avoid using, are provided in Table 4 and Supplementary Table 3.

### Subtypes of Bone Marrow Adipocytes

One important concept in BMA research is that BMAds and BMAT display distinct characteristics depending on skeletal location. This was first shown in 1965 by Cohen and Gardiner, who found that during starvation in rabbits, lipid is mobilised from BMAds within the proximal red marrow but not from BMAds in the distal yellow marrow (28). Subsequent work in the 1970s extended these observations, including the demonstration of distinct lipid composition in proximal vs. distal BMAds [reviewed in (113)]. This concept was relatively overlooked until the mid 2010s, when Scheller et al. proposed the existence of “regulated” and “constitutive” subtypes of BMAds (73, 114). Further details, including the evidence supporting these two subtypes, are provided in an excellent recent review (113). Therein, regulated BMAds are “*defined histologically as single adipocytes interspersed within the haematopoietic BM. They form gradually throughout life and accumulate with aging*.” In contrast, constitutive BMAds “*form early in development, are larger in size, and appear histologically as densely packed groups of adipocytes with little intervening haematopoiesis”* (113).

The terms regulated and “constitutive” have since gained traction in the field, as evident through the increasing use of the abbreviations “rMAT” or “rBMAT” and “cMAT” or “cBMAT” to refer to these subtypes (Table 5). However, this classification raises an important question: are there only two general classes, or is the heterogeneity less binary than this? For example, in the mouse tibia, proximal (“regulated”), and distal (“constitutive”) BMAds display different properties, in particular in terms of fatty acid saturation, but, like their “regulated” counterparts, the “constitutive” BMAds can also be altered in certain contexts (115, 116). Moreover, in some models of lipodystrophy there is a loss of cBMAT at the distal tibia, whereas cBMAT in caudal vertebrae is maintained (117). Thus, like rBMAT, cBMAT can also display plasticity in response to environmental cues, and cBMAT properties vary depending on skeletal site. Finally, most studies of cBMAT and rBMAT are based in animal models; hence, it remains unclear to what extent these exist as distinct subtypes in humans.

Because of these complexities, as a priority we recommend referring to BMAT and BMAd subtypes based on their anatomical location, in preference to using the “constitutive” and “regulated” terminology. This approach, which already is common in the literature (Table 5), provides a definitive, unambiguous description that addresses the issue of site-specific characteristics. However, given the increasing use of “constitutive” and “regulated,” and the evidence supporting existence of these two broad subtypes (113), we accept that there is still value in continued use of these terms. Therefore, if authors do wish to use these terms, we recommend using rBMAT and cBMAT to refer to the regulated and constitutive subtypes of BMAT, and rBMAd and cBMAd when referring to these subtypes of BM adipocytes.

Further details of these recommendations, and the many terms that we recommend to avoid using, are provided in Table 5 and Supplementary Table 4.

### Progenitors for Bone Marrow Adipocytes

BMAds originate from marrow-derived skeletal stem cells (SSCs) (72, 118). These cells go by different names, based on two different concepts regarding their differentiation properties and their tissue of origin. The first concept emanates from *in vivo* transplantation studies. These studies confirm the existence of multipotent progenitors present in BM stroma that can generate heterotopic bone/marrow organs (ossicles) with donor-derived skeletal cell phenotypes (chondrocytes, osteoblasts, stromal cells), including BMAds [reviewed in (119)]. The term Bone Marrow Stromal Cell (BMSC) is a time-honoured term used to indicate the population of non-hematopoietic, non-endothelial, rapidly adherent cells isolated from BM; adherence in culture is one characteristic property of these stromal cells. Clonal analysis of BMSCs coupled with *in vivo* transplantation revealed the presence of a *subset* of multipotent cells (120, 121). Later, these multipotent cells were found to be sinusoidal pericytes, cells that wrap around blood vessels providing them with stability. Furthermore, they were found to restore the perivascular compartment from which they originate (the ability to self-renew), making these cells bona fide SSCs (122). Thus, SSCs are a multipotent, self-renewing subset of BMSCs: all SSCs are BMSCs, but not all BMSCs are SSCs. It is essential to emphasise this because these terms have been used inconsistently in the literature, causing confusion about the relationship between SSCs and BMSCs.

Notably, the term “skeletal stem cell” currently describes a biological activity rather than a well-defined cell phenotype, since the specific identity of multipotent and self-renewing SSCs is not yet clear. As discussed in the accompanying BMAS Methodologies position paper by Tratwal et al., agreement on how to purify SSCs has not yet been reached: even populations highly enriched by using certain cell surface markers are still not homogeneous. Nonetheless, the use of the term “SSC” is appropriate based on the retrospective evidence of stemness demonstrated by the generation of ossicles by clonal populations of BMSCs. However, it is important to note that other populations of SSCs have recently been identified in the growth plate and in the periosteum in both mice and humans, but it does not appear that SSCs from these origins contribute to BMA [reviewed in (123)] (104, 123–126). Similarly, stromal cells capable of adipogenesis *ex vivo* have been isolated from cortical bone of the femoral diaphysis and cancellous/cortical bone of the proximal epiphysis of rats (127, 128). Although it remains to be confirmed if these cells can generate BMAds *in vivo*, their *ex vivo* adipogenic capacity differs depending on skeletal site. This echoes the site-specific differences in BMAd subtypes discussed in the previous section. Thus, the tissue origin of SSCs must always be identified. Indeed, not all populations of SSCs give rise to adipocytes, as determined by *in vivo* transplantation or lineage tracing in mice. Of note, “*in vivo*” is the operative term here, as it is known that the *in vitro* assay for adipogenesis is prone to artefact. In many fibroblastic populations, a few cells accumulate fat from serum in the medium, but do not synthesize triglycerides or hydrolyse them to the same extent as *bona fide* adipocytes.

According to the second concept, the term “Mesenchymal Stem Cell (MSC)” was introduced, based on the ability of BMSCs/SSCs to make cells and tissues of *mesodermal* origin. These include not only skeletal cell types (chondrocytes, osteoblasts and BMAds) but also non-skeletal phenotypes such as myoblasts, tenocytes, fibrocytes (ligament), white adipocytes, and others (124). However, as defined by developmental biologists, mesenchyme is an embryonic connective tissue that makes connective tissue, blood, and blood vessels during foetal development, and is not found in post-natal tissue (129). In spite of this, it was proposed that “MSCs” reside in all post-natal tissues, based on non-specific cell surface markers shared by virtually all fibroblastic cells (130), and that all “MSCs” are pericytes (125), able to give rise to osteoblasts, chondrocytes and adipocytes, making them equivalent to SSCs within the BM. However, during embryonic development, none of the non-skeletal cell types, including pericytes, have a common embryonic origin (118). Lastly, the “MSC” term emerged from less-than-rigorous *in vitro* studies; more-recent *in vivo* work has confirmed that the progenitor activity of different populations of perivascular stromal cells (if any) is always restricted to that of the tissue of origin (126).

For these reasons, the use of the term “Mesenchymal Stem Cell” and any other definition including the word “Mesenchymal,” especially the use of the acronym “BMSC” to indicate bone marrow *mesenchymal stem* cells rather than bone *marrow stromal* cells, is not scientifically accurate and is not recommended. Thus, we recommend using “SSCs” to refer to “Skeletal Stem Cells” and “BMSCs” to refer to “Bone Marrow Stromal Cells.” Describing the anatomical location from which these cells are isolated, as well as the species of origin, is also important when reporting studies of SSCs and BMSCs.

Further details of these recommendations, and the terms and abbreviations that we recommend to avoid using, are provided in Table 6 and Supplementary Table 5.

### BMAT Morphometric Analyses

The elaboration of an appropriate nomenclature for BMAT morphometry cannot be conceived without considering the guidelines for bone histomorphometry that have been established for more than 25 years (5, 131). Indeed, many of the terms usable for BMAT morphometry are well-defined and properly abbreviated in the first Standardized Nomenclature, Symbols, and Units for Bone Histomorphometry (131) and in its revision (5).

As for bone, BMAT morphometry can be based on two- or three-dimensions (2D or 3D) and applied to many types of biological material, most commonly iliac crest bone/BM biopsies obtained from human subjects, and bones from experimental animals. Recent studies have also analysed BMAds within BM plugs of transgenic mice, allowing study of BMAd development by lineage tracing (132). A mixture of terminology for 2D and 3D techniques of analysis should not be used in the same article, unless different methods of analysis (i.e., histology vs. micro-CT) are used (5).

Both primary measurements and referents (i.e., some clearly defined area or volume within the sample) must be considered. The former include Adipocyte Area (Ad.Ar), Volume (Ad.V), Perimeter (Ad.Pm), Diameter (Ad.Dm), and Number (N.Ad); each of these abbreviations is consistent with the guidelines for bone histomorphometry (5). In addition to adipocyte number, measurements of the size of individual adipocytes (Ad.Ar, Ad.V, Ad.Pm, Ad.Dm) are important in the physiology and pathology of BMAT. Indeed, changes in total BMAT may be the result of mechanisms that lead to the increase or decrease of either BMAd size (lipid storage/lipolytic activity) or number (adipogenic differentiation, or BMAd apoptosis and clearance). Size-related measurements may have meaning *per se* and not require a referent. For example, they can be used for the calculation of mean (and median) of BMAds and to construct distribution curves; the latter provide a more accurate representation of BMAd sizes across the sample and therefore are the recommended method of presenting such data (Table 7).

In contrast to these measurements of BMAd size, for N.Ad, total adipose tissue area and total adipose volume, referents are needed, and must be properly defined. Since BM is distributed throughout the cavities of the skeleton and includes distinct kinds of tissues/cells (haematopoietic cells, BMAds, and BMSCs) and is highly vascularised, the referents may be different. Some of them (i.e., Tissue Area in 2D and Tissue Volume in 3D) have been already defined and abbreviated (T.Ar and TV, respectively) in the Standardized Nomenclature, Symbols, and Units for Bone Histomorphometry (5). For studies of BMA, additional referents to be considered include Marrow Area (Ma.Ar, in 2D) and Marrow Volume (Ma.V, in 3D), which coincide with the spaces of the skeleton delimited by endosteal surfaces (cancellous bone surfaces and endocortical surfaces).

The choice between T.Ar (or TV) or Ma.Ar (or Ma.V) as referents is important for the accurate interpretation of the data. For example, when osteopenia does occur, the Ma.Ar (or Ma.V) increases while T.Ar (or TV) does not. In addition, since haematopoietic cells and adipocytes are located in the Extra-Vascular compartment of the BM, the use of this region (Area: ExVa.Ma.Ar, i.e., Marrow Area *minus* Vascular Area; Volume: ExVa.Ma.V, i.e., Marrow Volume *minus* Vascular Volume) as referent could be more appropriate and, in principle, to be preferred in respect to Ma.Ar and Ma.V. Indeed, this Extra-Vascular compartment can be used as a referent not only for assessments of BMA (i.e., with total Ad.Ar, total Ad.V, or N.Ad as the numerator), but also for measuring the percentage of haematopoietic marrow. The latter is calculated by expressing the haematopoietic area (Hm.Ar) or haematopoietic volume (Hm.V) relative to ExVa.Ma.Ar or ExVa.Ma.V, respectively. As discussed in the accompanying BMAS Methodologies paper, the ratio of haematopoietic to adipose area (Hm.Ar./Ad.Ar.) may also be of interest in some instances but should not be confounded with measures of adiposity (e.g., Ad.Ar/Ma.Ar) or hematopoietic cellularity (e.g., Hm.Ar/Ma.Ar).

One challenge is that measurement of the Extra-Vascular Marrow compartment can be highly time-consuming. Thus, we suggest to use the area (or volume) of the Extra-Vascular Marrow compartment as referent in specific conditions only, for example in mouse long bone whole-mount sections in which the vascular spaces (sinusoids and central vein) may constitute a very significant and dynamic area or volume (133); and, in humans, in those haematological diseases in which BM vascularity is known to be altered (134). Use of the Extra-Vascular Marrow referent is, however, still limited by technological challenges, which may resolve as whole-mount 3D microscopy and high-resolution contrast-enhanced μCT become more widely available to reconstruct, and subtract, the vascular network within the BM space (135, 136). Subtraction of the central vein from the Marrow Area is, however, common in whole-bone histology of murine long bones due to the dilated lumen often associated with retraction artefacts upon fixation (reviewed in the accompanying BMAS Methodologies position paper by Tratwal et al.).

A final consideration, debated extensively among WG members, is whether to use the term “Adipocyte density” or “Adipocyte number” when describing measurements of N.Ad relative to a referent region (i.e., Ma.Ar, Ma.V, T.Ar, TV, ExVa.Ma.Ar, or ExVa.Ma.V). Such measurements report the population density of BMAds within the region of interest, and therefore we recommend that these are reported as “Adipocyte density” (Table 7). However, one concern with the term ‘density' is that readers may confuse this with a measurement of the actual physical density of BMAds (i.e., mass per unit volume of the cells); this concern was raised in the published guidelines for bone histomorphometry (5), which therefore prioritises the term “number” over “density” when reporting relative cell numbers. Thus, to be consistent with these previous guidelines, we agree that use of “Adipocyte number” is also acceptable when describing measurements of N.Ad relative to a referent region. What is essential is that studies should never report measurements of N.Ad alone: a suitable referent must always be used.

Details of the proposed nomenclature regarding BMAT morphometry are presented in Table 7, with further information in Supplementary Table 6.

### MRI- and/or MRS-Based Analyses

MRI analysis of BMA was first reported in the mid-1980s (53–57), but the past 20 years have seen a notable increase in publications using MRI and/or 1H-MRS for assessment of BMA (Table 8 and Supplementary Table 7). Most studies report the fat fraction (FF) or lipid:water ratio, calculated from the lipid and water peaks in MR spectra (71); however, there is much inconsistency in how these measurements are reported. As shown in Table 8, measurements described by the term “fat fraction (FF),” or the related terms “marrow fat fraction” or “bone marrow fat fraction (BMFF),” typically are reported as a %, but in some cases the actual measurement reported is the lipid/water ratio and not the % fat fraction. Conversely, sometimes measurements described as a “ratio” are actually reported as % fat fraction. Confusing issues further, some studies have used the term “fat content” when reporting measurements of % fat fraction, whilst others have used this term when reporting the lipid/water ratio. In other cases, the terms “fat fraction” or “fat content” are used, but the units of measurement are not stated; this may cause confusion about whether the measurement is a fraction or a ratio. Finally, MRI has been used to report the volume or area of BMA from 3D or 2D images, respectively (Table 8).

This variability underscores the need for standardisation. To date, most studies use the term “fat fraction,” “marrow fat fraction,” or “bone marrow fat fraction” to report the % fat fraction of the BM (Table 8). These measurements typically are based on dual-echo MRI or MRS data, which can be subject to confounding factors (e.g., attenuation of the T2^*^ signal) (71). In contrast, measurement of the proton density fat fraction (PDFF), based on multi-echo data, removes the effect of these confounding factors and thereby represents a truly standardised BMA measurement (71). Thus, in addition to 1H-MRS, PDFF is emerging as another gold-standard for BMA quantification. Nevertheless, many studies report dual-echo fat fraction measurements. We recommend reporting MRI and 1H-MRS-based measurements as fat fraction (FF) or bone marrow fat fraction (BMFF) when based on dual-echo data, and as PDFF when based on appropriate multi-echo sequences. These measurements should be reported as %, rather than the lipid/water ratio.

A final issue is the ability of MR to measure the degree of lipid saturation. Terms such as “unsaturation index” (UI) and “unsaturated lipid fraction” (ULF) have been used (137–141), and this capacity to assess not just BMAT quantity, but also BMAT quality, may reveal further insights about the clinical implications of altered BMA (71, 99). However, compared to reports of FF, BMFF or PDFF, there are far fewer papers reporting BMAT saturation, and therefore we feel it is too early to recommend terms, abbreviations or units for describing such measurements.

Further details of these recommendations, and the terms that we recommend to avoid using, are provided in Table 8 and Supplementary Table 7.

### CT-Based Analyses

As for MRI, CT-based BMA analysis was first reported in the mid-1980s (58, 59, 61). However, the application of these two approaches has since diverged: MRI has found widespread use for clinical BMA assessment *in vivo* but remains relatively under used in preclinical animal studies. In contrast, only a handful of studies have used CT to measure BMA *in vivo*, whereas its preclinical use has flourished in recent years, particularly for μCT (Table 9). The latter is primarily a result of the development of contrast agents, such as osmium tetroxide, that allow μCT-based visualisation and quantification of BMA *ex vivo*.

Clinically, both single-energy CT (SECT) and dual-energy CT (DECT) have been used to assess BMA *in vivo* and *ex vivo*. SECT-based measurements of BM density, expressed in Hounsfield Units, correlate inversely with BMA; however, these measurements include the density of yellow and red marrow as well as trabecular bone, and therefore do not allow for direct quantification of BMA (142). Nevertheless, several recent studies have used SECT to estimate relative adipose volume within the BM based on HU thresholds indicative of adipose tissue (108, 143). In contrast to SECT, DECT is able to separate BMA from the other tissue components, allowing true quantification of BMA. Such DECT-based measurements, reported as fat fraction (Table 9), show good agreement with MRI/MRS-based assessment of BMA (142, 144, 145). Thus, for consistency with nomenclature for histomorphometry and MRI/MRS-based measurements (Tables 7, 8), we recommend that CT-based assessment of BMA should be reported as fat fraction (FF, %), bone marrow fat fraction (BMFF, %), or adipose volume (Ad.V/Ma.V, %).

*Ex vivo*, staining of tissue samples with contrast agents allows direct measurement of adipose volume by μCT. Most such studies report Ad.V as the % or fraction relative to Ma.V or TV, although a sizeable minority have reported absolute Ad.V; in some cases, it is not clear if absolute or relative adipose volume is being reported (Table 9). Consistent with our proposals for BMAT histomorphometry (Table 7), we recommend that μCT-based readouts of adipose volume should be reported relative to Ma.V (i.e., as Ad.V/Ma.V, %). For the referent, Ma.V should be prioritised over TV because the latter can include bone and other tissues that necessarily exclude BMAds; the size of these tissues may also vary based on the physiological or pathological context, confounding analysis of BMA. Thus, presenting Ad.V/Ma.V provides a more accurate assessment of BMA.

An emerging application of μCT is the ability to measure the number and size of individual BMAds. Adipocyte density (i.e., N.Ad/Ma.V) has been reported for samples stained with osmium tetroxide (146) or with a Hafnium-based polyoxometalate contrast agent (135). Similarly, nano-CT has been used to quantify the area of individual BMAds in 2D image slices of osmium tetroxide-stained samples, allowing the distribution of individual BMAd areas to be determined (147). As for BMAT histomorphometry, we recommend reporting these readouts as “Adipocyte density” (N.Ad/Ma.V) and the frequency distribution of “Adipocyte area” (Ad.Ar, μm^2^), respectively; “Adipocyte number” may also be used in place of “Adipocyte density,” as discussed in the section BMAT Morphometric Analyses. An obvious extension of these methods would be the measurement of total adipocyte area in 2D slices of CT scans, and of individual adipocyte volume in 3D scans. While such readouts have not yet been published (Table 8), for the sake of foresight we recommend that these should be reported, respectively, as “Adipose area” (Ad.Ar/Ma.Ar, %) or frequency distributions of “Adipocyte volume” (Ad.V, μm^3^).

Further details of these recommendations, and the terms that we recommend to avoid using, are provided in Table 9 and Supplementary Table 8.

## Concluding Remarks

The study of BMA is a dynamic, vibrant, and expanding field of research. As populations and sub-populations of cells are more accurately defined, it may be necessary to reappraise the nomenclature to accommodate subtle nuances found between cells that may influence function. Similarly, it is likely that the BMA nomenclature will have to be updated to reflect advances in methodologies relevant to BMA research. The Nomenclature Working Group of the International Bone Marrow Adiposity Society will therefore endeavour to regularly reassess the field and make practical recommendations on nomenclature to help define and characterise the cells and environment that initiate, propagate and maintain BMA, and the methods used to assess these. Such future updates will depend on the continued growth and development of the BMA field. For now, we hope that the nomenclature guidelines herein will provide a foundation to support this growth; to promote collaboration; and to provide a consensus for the diverse fields of study related to BMA.

## Author Contributions

WC conceptualized the manuscript and coordinated the literature review, tables, figure, and assembly of the different sections written by the other authors. WC led the writing for the sections on Bone Marrow Adiposity, Bone Marrow Adipose Tissue, Subtypes of Bone Marrow Adipocytes, and CT-based analyses. CC led the writing for the Abstract. TS led the writing for the section on Bone Marrow Adipocytes. MR, PR, and AC wrote the section on Progenitors for Bone Marrow Adipocytes. AC wrote the section on BMAT Morphometric Analyses, with MR providing additional input. MB and WC wrote the section on MRI- and/or MRS-Based Analyses. WF led the writing of the Concluding Remarks. All authors, including NB, SR-S, ED, and CR discussed the terminology identified and, through several meetings, agreed on the nomenclature recommendations. All authors edited and approved the final version of the manuscript.

### Conflict of Interest

The authors declare that the research was conducted in the absence of any commercial or financial relationships that could be construed as a potential conflict of interest.
