# Supplementary material for: Standardised Nomenclature, Abbreviations, and Units for the Study of Bone Marrow Adiposity: Report of the Nomenclature Working Group of the International Bone Marrow Adiposity Society
Source: Front Endocrinol (Lausanne). 2020 Jan 24;10:923. doi: 10.3389/fendo.2019.00923 (PMC6993042; doi:10.3389/fendo.2019.00923)
Supplement: Supplementary file 1 [file Data_Sheet_1.pdf]

frontiers

Supplementary Material for

Standardised Nomenclature, Abbreviations, and Units for the study of Bone Marrow Adiposity: Report of the Nomenclature Working Group of the International Bone Marrow Adiposity Society.

Nathalie Bravenboer, Miriam A. Bredella, Christophe Chauveau, Alessandro Corsi, Eleni Douni, William F. Ferris, Mara Riminucci, Pamela G. Robey, Shanti Rojas-Sutterlin, Clifford Rosen, Tim J. Schulz, and William P. Cawthorn

Author affiliations and contributions are described in the main manuscript.

Inventory of Supplementary Material:

Supplementary Table 1 (related to Table 2): Full list of all terms and abbreviations that have been used to refer to bone marrow adiposity, yellow marrow, fatty marrow or red marrow.

Supplementary Table 2 (related to Table 3): Full list of all terms and abbreviations that have been used to refer to bone marrow adipocytes.

Supplementary Table 3 (related to Table 4): Full list of all terms and abbreviations that have been used to refer to bone marrow adipose tissue, yellow adipose tissue or marrow fat.

Supplementary Table 4 (related to Table 5): Full list of all terms and abbreviations that have been used to refer to subtypes or site-specific differences in BMAT and BMAds.

Supplementary Table 5 (related to Table 6): Full list of all terms and abbreviations that have been used to refer to progenitors for BMAds.

Supplementary Table 6 (related to Table 7): Full list of all terms, abbreviations and units that have been used to refer to histomorphometric measurements related to BMA.

Supplementary Table 7 (related to Table 8): Full list of all combinations of terms, abbreviations and units that have been used to report MRI/MRS-based measurements of bone marrow adiposity.

Supplementary Table 8 (related to Table 9): Full list of all terms, abbreviations and units that have been used to report CT-based measurements of bone marrow adiposity.

Supplementary References: Includes 560 references for publications cited in the Supplementary Tables.

Term	Abbreviation	Year first used*	No. of papers using this term*	References
Bone marrow adiposity				
marrow adiposity	*none*	1967	119	[1-119]
bone marrow adiposity	*none*	2002	78	[5; 7; 9; 10; 12; 22; 26; 27; 33; 34; 36; 37; 39; 40; 48; 49; 52; 57; 60; 62; 67; 79; 80; 82; 83; 88; 92; 107; 111-113; 116; 120-165]
	BM adiposity	2011	12	[54; 104; 110; 166-174]
	BMA	2013	9	[114; 175-182]
	BMAT	2017	1	[183]
bone adiposity	*none*	2009	4	[7; 74; 112; 134]
adiposity	*none*	2017	2	[85; 104]
yellow bone marrow adiposity	yellow BMA	2013	1	[175]
proximal tibia adiposity	*none*	2018	1	[172]
yellow marrow				
yellow marrow	*none*	1883	108	[8; 34; 40; 43-45; 47; 49; 55; 58; 65; 73; 78; 82; 87; 89; 91; 101; 110; 117; 134; 137; 151; 158; 159; 164; 167; 168; 171; 174; 176; 178; 180; 181; 184-258]
	YM	2010	1	[259]
yellow marrow (inactive marrow)	*none*	2003	1	[260]
yellow bone marrow	*none*	1936	23	[40; 101; 118; 138; 160; 175; 187; 188; 220; 261-274]
	YBM	1967	2	[275; 276]
	yellow BM	1996	2	[277; 278]
	YM	2019	1	[256]
yellow bone marrow adiposity	yellow BMA	2013	1	[175]

yellow fatty marrow	*none*	1933	7	[116; 186; 195; 270; 279]
yellow fatty bone marrow	*none*	2017	1	[280]
yellow fat marrow	*none*	1979	1	[194]
adipocytic yellow marrow	*none*	2015	1	[43]
yellow adipocyte-rich marrow	*none*	2017	1	[73]
yellow adipose marrow	*none*	2018	1	[281]
aplastic (yellow) marrow	*none*	1974	1	[282]
Fatty marrow				
fatty marrow	*none*	1922	82	[5; 32; 48; 51; 52; 56; 69; 78; 84; 86; 94; 97; 117; 123; 132; 164; 170; 176; 186; 187; 189; 190; 196; 200; 202; 203; 205; 206; 208; 210-216; 219; 221; 222; 238; 245; 246; 250; 251; 253; 259; 262; 266; 271; 277; 280; 282-312]
fatty bone marrow	*none*	1990	7	[264; 265; 280; 313-316]
	fatty BM	2008	1	[317]
fat marrow	*none*	1985	1	[201]
fat bone marrow	FBM	2014	1	[267]
fatty yellow marrow	*none*	2013	7	[29; 114; 138; 142; 223; 280; 318]
fatty portion of the marrow	*none*	1949	1	[319]
aplastic (fatty) marrow	*none*	1974	1	[282]
Red marrow				
red marrow	*none*	1883	122	[6-8; 29; 34; 43-45; 47; 49; 55; 58; 65; 73; 77; 78; 82; 87; 89; 98; 101; 110; 114; 117; 123; 134; 137; 138; 140; 142; 151; 158; 159; 164; 167; 168; 171; 173; 174; 176; 178; 180; 184-187; 189-193; 195; 196; 204; 206-221; 223-229; 231-242; 245-253; 255-258; 268; 280; 282-284; 287; 289; 290; 292; 302; 310; 311; 320-327]
	RM	2010	2	[259; 318]

red marrow (active marrow)	*none*	2003	1	[260]
red bone marrow	*none*	1922	21	[40; 84; 101; 118; 138; 160; 173; 175; 187; 243; 263; 266; 271; 273; 274; 278; 283; 328-331]
	red BM	1996	2	[272; 277]
	RM	2019	1	[256]
	RBM	2015	2	[276; 332]
red distal bone marrow	*none*	1979	1	[194]
red femoral marrow	*none*	1933	2	[194; 285]
h(a)em(at)opoietic marrow	*none*	1936	24	[5; 7; 91; 97; 117; 134; 167; 176; 187; 190; 191; 218; 221; 245; 251; 253; 271; 282; 291; 292; 309; 333-335]
red h(a)em(at)opoietic marrow	*none*	2017	5	[114; 116; 254; 270; 280]
h(a)em(at)opoietic red marrow	*none*	1979	4	[110; 123; 194; 250]
h(a)em(at)opoietic bone marrow	*none*	1979	3	[9; 266; 336]
	HBM	2014	1	[267]
red h(a)em(at)opoietic bone marrow	*none*	2017	2	[73; 281]
erythropoietic marrow	*none*	2018	1	[246]
nonfatty marrow	*none*	1995	1	[211]

Supplementary Table 1 (related to Table 2): Full list of all terms and abbreviations that have been used to refer to bone marrow adiposity, yellow marrow, fatty marrow or red marrow. Relevant terms and abbreviations were identified through a systematic search of the literature on bone marrow adiposity (BMA). Based on this search, citations for papers using each term/abbreviation combination are shown in the right-hand column, with the total number of papers and the year of first use shown in the preceding columns; asterisks are shown to emphasise that other uses may exist. In some cases, no abbreviation is used (*none*). Recommended terms and abbreviations are presented in Table 2.

Term	Abbreviation	Year first used*	No. of papers using this term*	References
Bone marrow adipocyte				
bone marrow adipocyte	*none*	1976	100	[7; 13; 14; 17; 23; 26; 29; 30; 33; 43; 45; 49; 56; 57; 67; 71; 72; 76; 77; 84; 92; 100; 103; 105; 108; 111; 112; 114; 115; 123-126; 128; 130; 135; 136; 139; 142; 148; 155; 156; 162-165; 177; 179; 204; 228; 236; 237; 242; 244; 271; 272; 307; 316; 331; 337-377]
	BM adipocyte	1995	30	[54; 63; 78; 93; 110; 153; 167; 169-172; 176; 181; 239; 278; 306; 317; 318; 322; 323; 378-387]
	BMA	2016	17	[65; 74; 104; 107; 157; 168; 174; 249; 268; 273; 324; 327; 388-392]
	BMAd	2019	2	[182; 257]
	MAT adipocyte	2015	6	[68; 234; 238; 247; 280; 325]
	BMAT adipocyte	2018	3	[91; 97; 388]
	BM-A	2013	2	[266; 326]
	BM-AD	2018	2	[173; 393]
	marrow AC	2016	1	[147]
marrow adipocyte	*none*	1978	105	[3; 6-8; 17-19; 26; 29-32; 39; 41; 42; 47; 54; 58; 61; 62; 66; 73; 77; 82; 84; 86-88; 94-96; 100; 101; 106; 108; 110; 111; 113; 116; 119; 123; 124; 126; 128; 139; 142; 143; 151; 152; 154; 159; 162; 178; 180; 194; 217; 218; 231; 234; 238; 242; 248; 253; 254; 260; 280; 281; 308; 321; 323; 331; 335; 342; 345; 347; 349; 352; 358; 361; 362; 365; 369; 375; 392; 394-414]
adipocyte	*none*	1981	39	[52; 55; 57; 69; 70; 72; 83; 85; 88; 90; 99; 109; 117; 118; 154; 158; 183; 205; 221; 222; 240; 252; 260; 269; 274; 279; 309; 312; 334; 415-424]
	AC	2018	1	[251]
adipose cell	*none*	1971	16	[191-193; 196; 198-200; 221; 277; 278; 282; 291; 415; 425-427]
	Adip	2005	1	[314]
marrow adipose cell	*none*	1971	8	[33; 191; 291; 292; 337; 425; 428; 429]

bone adipocyte	*none*	1981	6	[26; 366; 430-433]
bone marrow adipose cell	*none*	1974	3	[292; 337; 425]
marrow-associated adipose cell	*none*	1974	1	[425]
medullary adipocyte	*none*	2010	1	[357]
skeletal adipocyte	*none*	2015	1	[234]
yellow adipocyte	*none*	2019	1	[173]
Bone marrow fat cell				
fat cell	*none*	1893	48	[82; 118; 140; 163; 185; 187; 189; 191; 192; 195; 197; 214; 259; 265; 285; 290-292; 298; 317; 330; 336; 337; 348; 416; 418; 434-455]
	FC	1935	1	[287]
fatty cell	*none*	1985	3	[295; 314; 456]
marrow fat cell	*none*	1931	14	[58; 96; 192-195; 294; 298; 329; 402; 425; 434; 457; 458]
bone marrow fat cell	*none*	1966	10	[29; 162; 337; 359; 399; 459-463]
	BM fat cell	1998	1	[379]
fat-containing cell	*none*	1980	2	[330; 464]
	FCC	1983	1	[465]
fat-storage cell	*none*	1986	2	[296; 297]
Other terms				
fat spaces	*none*	1934	1	[328]
giant fat cell	*none*	1981	1	[466]
unilocular fat cell	*none*	1999	1	[460]
mature adipocyte	*none*	2004	2	[315; 467]
fat-accumulating cell	*none*	2007	1	[265]
fibroblast-like fat cell	FLFC	2008	1	[317]
marrow-resident adipocyte	*none*	2017	1	[73]
yellow fat cell	*none*	2017	1	[87]

Supplementary Table 2 (related to Table 3): Full list of all terms and abbreviations that have been used to refer to bone marrow adipocytes. Data were obtained and are presented as described for Supplementary Table 1. Recommended terms and abbreviations are presented in Table 3.

Term	Abbreviation	Year first used*	No. of papers using this term*	References
Bone marrow adipose tissue				
adipose tissue	*none*	1883	17	[112; 146; 184; 193; 194; 198; 199; 207; 252; 261; 291; 307; 333; 334; 345; 439; 468]
adipose tissue from bone	*none*	1999	1	[460]
adipose marrow	*none*	1991	4	[88; 218; 326; 468]
adipose marrow tissue	*none*	2017	1	[88]
marrow adipose	*none*	1971	2	[87; 291]
marrow adipose tissue	*none*	1971	31	[31; 38; 59; 75; 87; 94; 95; 118; 119; 129; 149; 150; 162; 177; 193; 195; 196; 236; 256; 291; 292; 298; 342; 344; 368; 396; 408; 410; 422; 425; 469]
	MAT	2012	56	[21; 44; 47; 53; 58; 62; 66-68; 71; 76; 78; 82; 83; 85; 89; 90; 101; 102; 106; 108; 116; 117; 145; 148; 152; 159; 168; 183; 234; 238; 242; 248; 250; 253; 254; 269; 271; 272; 278; 280; 281; 306; 323; 325; 400; 403-406; 470-475]
marrow-associated adipose	*none*	1974	1	[425]
bone marrow adipose tissue	*none*	1974	34	[8; 46; 65; 85; 100; 109; 163; 177; 191; 194; 230; 236; 251; 254; 273; 274; 301; 338; 365; 368; 389; 399; 422; 424; 429-432; 476-481]
	BMAT	2007	49	[40; 73; 75; 77; 82; 91; 93; 97; 103-105; 107; 111; 150; 153; 155; 164; 168; 170-172; 174; 176; 178; 179; 182; 220; 224; 247; 249; 256-258; 268; 272; 305; 315; 322; 327; 331; 388; 391; 393; 410; 482-486]
	BM adipose tissue	1988	6	[181; 235; 239; 298; 387; 392]
	MAT	2014	15	[80; 110; 165; 167; 169; 247; 318; 370; 377; 385; 386; 400; 411; 412; 487]
	BM-AT	2019	1	[173]

bone marrow adipose	*none*	1979	5	[182; 274; 353; 375; 426]
Yellow adipose tissue				
yellow adipose tissue	*none*	1883	2	[184; 228]
	YAT	2011	4	[135; 272; 321; 398]
yellow adipose	*none*	2016	1	[322]
yellow fat	*none*	1933	2	[186; 331]
yellow adipose marrow	*none*	2018	1	[281]
fatty tissue	*none*	1922	1	[283]
fatty yellow adipose tissue	*none*	2019	1	[164]
bone marrow fatty tissue	*none*	1993	1	[341]
Marrow fat				
marrow fat	*none*	1954	101	[7-14; 17; 19; 21; 23; 24; 29; 30; 34; 38; 41; 44; 49; 54-56; 61; 66; 69; 70; 82; 84; 86; 96; 99; 114; 115; 118; 119; 125; 131; 133; 143; 145; 147; 149; 151; 193; 194; 205; 220; 222; 240; 248; 255; 259; 275; 300-303; 306; 312; 313; 321; 323; 324; 355; 395; 398; 401; 420; 425; 441; 456; 457; 468; 473; 480; 488-512]
	MF	2008	3	[4; 43; 45]
bone marrow fat	*none*	1936	80	[9; 13; 17; 27; 28; 30; 40; 46; 57; 73; 80; 87; 88; 125; 130; 135; 137; 138; 141; 144; 145; 149; 152; 160; 181; 183; 187; 188; 220; 229; 232; 233; 237; 240; 241; 243; 245; 255; 259; 276; 279; 302; 326; 332; 337; 343; 350; 354; 355; 360; 365; 376; 390; 398; 399; 414; 419; 422; 478; 480; 482; 485; 487; 504; 506; 509-511; 513-524]
	BM fat	2002	3	[78; 221; 382]
	BMF	2007	11	[55; 98; 114; 142; 158; 228; 251; 273; 320; 367; 371]
	MF	2014	1	[421]
medullary fat	*none*	1994	2	[512; 525]
bone fat	*none*	2010	1	[259]

Supplementary Table 3 (related to Table 4): Full list of all terms and abbreviations that have been used to refer to bone marrow adipose tissue, yellow adipose tissue or marrow fat. Data were obtained and are presented as described for Supplementary Table 1. Recommended terms and abbreviations are presented in Table 4.

Term used	Abbreviation	Year first used*	No. of papers using this term*	References
Constitutive or Regulated BMAT and BMAds				
constitutive marrow adipose tissue	cMAT	2014	26	[58; 67; 78; 97; 101; 102; 108; 110; 159; 165; 167; 168; 234; 238; 253; 254; 272; 273; 280; 318; 322; 325; 370; 405; 470; 510]
	constitutive	2017	1	[271]
constitutive bone marrow adipose tissue	cBMAT	2017	7	[73; 107; 164; 170; 171; 174; 249]
	constitutive	2017	2	[178; 391]
constitutive bone marrow adipocyte	cMAT adipocyte	2015	6	[68; 110; 234; 238; 280; 325]
	cBMA	2016	7	[157; 168; 249; 268; 324; 327; 389]
regulated marrow adipose tissue	rMAT	2014	26	[58; 67; 78; 97; 101; 102; 108; 110; 159; 165; 167; 168; 234; 238; 253; 254; 272; 273; 280; 318; 322; 325; 370; 405; 470; 510]
	regulated MAT	2017	1	[271]
regulated bone marrow adipose tissue	rBMAT	2017	7	[73; 107; 164; 170; 171; 174; 249]
	regulated BMAT	2017	2	[178; 391]
regulated bone marrow adipocyte	rMAT adipocyte	2015	6	[68; 110; 234; 238; 280; 325]
	rBMA	2016	8	[107; 157; 168; 249; 268; 324; 327; 389]
	none	2017	1	[73]
Distal or Proximal BMAT and BMAds				
distal marrow adipose tissue	distal MAT	2016	1	[318]
distal marrow fat	*none*	1979	1	[194]
distal tibia marrow adipose tissue	dMAT	2017	1	[242]
proximal marrow adipose tissue	proximal MAT	2016	1	[318]
proximal marrow fat	*none*	1979	1	[194]
proximal tibia marrow adipose tissue	pMAT	2017	1	[242]

Bone-specific terms				
femoral adipose cell	*none*	1977	1	[192]
femur marrow fat	FMF	1984	1	[526]
tibial adipose bone marrow	*none*	1985	1	[338]
vertebrae adipose cell	*none*	1977	1	[192]
vertebral fat	*none*	1989	1	[527]
intravertebral bone marrow fat	*none*	2002	1	[528]
vertebral bone marrow fat	*none*	2012	1	[529]
	VMB fat	2017	1	[79]
vertebral BMAT	vBMAT	2018	1	[179]
vertebral marrow fat	*none*	2012	1	[222]
vertebral MAT	vMAT	2018	1	[278]
BMAT within red or yellow marrow				
red marrow fat	*none*	1940	2	[188; 513]
red bone marrow fat	*none*	2001	2	[330; 332]
	RBM fat	2019	1	[276]
red marrow adipose tissue	rMAT	2018	2	[97; 410]
yellow marrow adipose tissue	yMAT	2018	2	[97; 410]
Other terms				
structured fat	*none*	1984	1	[188; 450; 513]

Supplementary Table 4 (related to Table 5): Full list of all terms and abbreviations that have been used to refer to subtypes or site-specific differences in BMAT and BMAds. Data were obtained and are presented as described for Supplementary Table 1. The term "structured fat" was used to refer to the constitutive-like BMAds that exist in contiguous groups, so is included here for this reason. Recommended terms and abbreviations are presented in Table 5.

Abbreviation	Term used	Year first used*	No. of papers using this term*	References
**SSC**	skeletal stem cell	2004	12	[72; 102; 103; 165; 178; 218; 231; 244; 325; 530-532]
	skeletal stem cell *(no abbreviation)*	2004	11	[161; 180; 181; 258; 393; 402; 413; 533-535]
	mouse skeletal stem cell (mSSC)	2015	1	[530]
	human skeletal stem cell (hSSC)	2018	1	[532]
**BMSC**	bone marrow stromal cell	2004	20	[23; 26; 36; 59; 64; 111; 133; 139; 161; 169; 178; 231; 280; 303; 315; 404; 484; 531; 534; 536]
	bone marrow mesenchymal stem cell	2013	5	[37; 165; 273; 365; 409]
	bone marrow mesenchymal stem cell	2012	1	[132]
	bone marrow stromal cell (mBMSC)**	2015	1	[49]
	bone mesenchymal stem cell	2016	1	[238]
	bone-derived marrow mesenchymal stem cell	2017	1	[154]
	bone marrow stromal cell (BM stromal cell)	2017	1	[168]
	bone marrow mesenchymal cell	2018	1	[389]
	bone marrow mesenchymal stromal cell	2018	1	[325]
	not defined	2018	1	[102]
	bone marrow mesenchymal stem/stromal cell	2019	1	[115]
BM stromal cell	bone marrow stromal cell	1995	3	[176; 378; 379]
BM mesenchymal stromal cell	bone marrow mesenchymal stromal cell	2018	1	[171]
MMC	multipotent mesenchymal cell	2012	1	[22]

MSC	mesenchymal stem cell	2003	59	[6; 8; 10; 16; 34; 37; 41; 42; 53; 56; 61; 62; 65; 66; 73; 78; 83; 85; 87; 92; 101; 108; 128; 139; 142; 152; 155; 156; 161; 164; 177; 228; 231; 239; 248; 254; 259; 265; 268; 270; 274; 281; 309; 316; 348; 357; 376; 377; 392; 402; 420; 433; 471; 473; 519; 531; 535-537]
	mesenchymal stromal cell	2012	10	[24; 88; 159; 174; 269; 322; 325; 372; 388; 393]
	abbreviation not defined	2015	5	[88; 242; 406; 472; 510]
	human mesenchymal stem cell (hMSC)	2004	4	[52; 154; 388; 533]
	bone marrow mesenchymal stromal cell	2014	4	[84; 93; 135; 167]
	mesenchymal stem/stromal cell	2016	4	[112; 151; 178; 272]
	marrow stromal cell	2003	3	[77; 348; 537]
	bone marrow mesenchymal stem cell	2015	3	[100; 110; 233]
	bone marrow stromal cell	2004	2	[347; 504]
	Human bone marrow stromal cell (hMSC)	2004	1	[417]
	bone marrow mesenchymal stem cell	2012	1	[132]
	mesenchymal progenitor	2016	1	[147]
	bone marrow-derived mesenchymal stem cell	2018	1	[90]
	bone mesenchymal stem cell	2018	1	[95]
BM-MSC	bone marrow mesenchymal stem cell	2013	5	[157; 172; 266; 387; 412]
	bone marrow mesenchymal stromal cell	2018	2	[326; 393]
	bone marrow MSC	2017	1	[239]
	bone marrow skeletal stem cell	2018	1	[172]
	bone marrow stromal stem cell	2019	1	[258]
No abbreviation used	mesenchymal stem cell	2004	38	[5; 31; 67; 77; 79-81; 94; 97; 100; 105; 106; 116; 117; 123; 126; 127; 156; 158; 159; 176; 218; 253; 258; 331; 354; 359; 361; 362; 411; 414; 423; 487; 505; 522; 524; 530; 532]
	bone marrow stromal cell	1990	18	[58; 88; 90; 124; 125; 128; 134; 163; 204; 304; 342; 364; 390; 410; 532; 533; 538]

	mesenchymal progenitor	2013	9	[77; 107; 115; 170; 182; 226; 248; 407; 413]
	marrow stromal cell	2003	4	[252; 418; 533; 535]
	bone marrow mesenchymal stromal cell	2010	4	[72; 85; 98; 125]
	mesenchymal progenitor cell	2017	4	[82; 119; 169; 249]
	bone marrow mesenchymal stem cell	2016	3	[69; 173; 374]
	mesenchymal stromal cell	2016	3	[72; 103; 486]
	mesenchymal cell	1970	2	[290; 291]
	bone mesenchymal stem cell	2017	2	[119; 480]
	bone marrow mesenchymal cell	2016	1	[69]
	bone marrow stem cell	2017	1	[75]
	mesenchymal precursor	2017	1	[74]

Supplementary Table 5 (related to Table 6): Full list of all terms and abbreviations that have been used to refer to progenitors for BMAds. Data were obtained and are presented as described for Supplementary Table 1. In many cases, the same abbreviation has been applied to several different terms. Conversely, in many cases terms have been used without ascribing an abbreviation, as indicated in the final section of this table. In some cases, the species of the cells is also indicated (** e.g. mBMSC for "mouse BMSC"). Recommended terms and abbreviations are presented in Table 6.

Measurement type	Described as	Abbreviations used	Units used	Year first used*	No.	References	Recommended term(s) and unit(s)
**adipocyte area** (per cell)	adipocyte size	*none*; Ad.Ar	μm^2, mm^2	2002	15	[15; 30; 36; 59; 60; 62; 80; 93; 111; 125; 130; 148; 350; 382; 393]	**Report as 'Adipocyte area' (Ad.Ar, μm^2).** *Showing the frequency distribution of Ad.Ar is recommended*
	adipocyte area	*none;* Ad.Ar	μm^2, mm^2, pixels	2012	10	[19; 172; 234; 318; 327; 368; 405; 406; 409; 412]	
	adipocyte size (cross-sectional area)	*none*	μm^2, area x 10^{-4} mm	2007	2	[239; 352]	
	femoral adipocyte vacuole area	*none*	μm^2	2011	1	[166]	
	adipocyte cross section area	*none*	mm^2	2017	1	[87]	
**adipocyte area** (total)	total adipocyte area	Ad.Ar	mm^2	2015	1	[45]	**Don't use** *(instead report as % Ma.Ar or % T.Ar)*
**adipocyte diameter** (per cell)	adipocyte diameter; mean adipocyte diameter; adipocyte mean diameter; fat cell diameter	none; MAD; Ad.Dm; Ad.MD	*n.s.*, µm	1989	16	[14; 31; 32; 41; 61; 94; 143; 155; 329; 346; 374; 377; 401; 418; 429; 507]	**Report as 'Adipocyte diameter' (Ad.Dm, µm)****
	adipocyte size; fat cell size; bone marrow fat cell size	none; Ad.Dm; Ad.Size	µm	1993	9	[25; 86; 96; 103; 308; 341; 461; 463; 477]	
**adipocyte perimeter** (per cell)	adipocyte perimeter	*none;* Ad.Pm	µm, mm	2002	4	[19; 349; 360; 382]	**'Adipocyte perimeter' (Ad.Pm, µm)****
	adipocyte circumference	Ad.C	mm^{-2}	2019	1	[104]	
**adipocyte perimeter** (total)	total adipocyte perimeter	Ad.Pm	mm	2015	1	[45]	**Don't use**

**adipocyte density** (cells per marrow area)	adipocyte density; bone marrow adipocyte density	Ad.D; Ad.Dn, Ma.V; %ATV	no/mm^2, cells/mm^2 marrow area, cells/mm^2 marrow volume, % adipose tissue volume	1990	6	[19; 31; 45; 163; 205; 377]	**Report as 'Adipocyte density' or 'Adipocyte number' relative to marrow area (N.Ad/Ma.Ar) or volume (N.Ad/Ma.V); or tissue area (N.Ad/T.Ar) or volume (N.Ad/TV).** Units should be clearly stated (e.g. cells/mm^2, cells/µm^2, etc...)
	adipocyte number; adipocyte no.	N.A/MV; BM	cells per marrow area, no./mm^2 bone marrow, % adipocytes in BM	2000	4	[239; 344; 382; 396]	
	adipocyte counts	*none;* N.At/M.Ar	cells/mm^2, cells/0.1.mm^2	2005	3	[63; 226; 349]	
	bone marrow adipocytes	*none*	cells/mm^2 marrow area	2012	1	[23]	
	number of fat cells per unit area	Na.Fa/Ma.V	cells/mm^2	2005	1	[351]	
**adipocyte density** (cells per tissue area)	adipocyte density; marrow adipocyte density	*none;* Ad.D; AV/TV; N.Ad.T.Ar	#/mm^2, #/µm^2, 1/mm^2, no./mm^2, cells/mm^2, %	1991	22	[18; 20; 30; 32; 41; 59-62; 80; 94; 112; 130; 143; 156; 308; 350; 352; 360; 397; 401; 468]	
	adipocyte number; bone marrow adipocyte number; number of adipocytes	*none*; Ad.N; AD#; N.Ad/T.Ar	/mm^2, #/mm^2, 1/mm^2, no./mm^2, mm-2, number/mm^2	2008	21	[14; 19; 37; 48; 64; 86; 87; 96; 104; 111; 125; 126; 148; 154; 353; 360; 374; 406; 409; 412; 539]	
	adipocyte number per mm^2; adipocytes/mm^2; adipocytes/µm^2; adipocyte number/cm2	*none*; Ad.N	adipocyte number per mm^2, adipocytes/mm^2, 1/mm^2, #/µm^2, adipocyte number/cm2, adipocytes/µm^2	2003	6	[42; 45; 54; 72; 102; 418]	
	adipocytes per total area; adipocytes/area; adipocyte number/tissue area	*none*; N.Ad/T.Ar	/mm^2, no.mm^2, #/µm^2	2010	3	[10; 113; 156]	
	adipocyte count	*none*	adipocytes/mm^2	2018	1	[92]	

**adipocyte density** (cells per marrow or tissue volume)	adipocyte number/marrow volume; cell number per μm^3	*none;* Ad.N/MV	adipocyte number/marrow volume, cells/mm^3, cells/μm^3	1989	5	[25; 51; 69; 244; 429]	**Report N.Ad/Ma.V or N.Ad/TV** (as described above)
	number of adipocytes per microliter	Nv	adipocytes/µL	2002	1	[346]	
	adipocyte density	Ad.Dn	cells/mm^3	2019	1	[103]	
**adipocyte density** (cells per field)	adipocyte number	*none*; AD/HPF	cells/field, cells/section, cells/10 serial sections, % cells field of view	2004	11	[6; 15; 33; 93; 155; 239; 244; 355; 364; 398; 467]	**Don't use**
	adipocyte density	AD/field	cells per field	2018	1	[393]	
**adipocyte number**	adipocyte number; adipocyte #; fat cell number; No. adipocytes	*none;* N.Ad	number	1993	10	[17; 127; 165; 234; 329; 341; 347; 352; 507; 540]	**Don't use** (instead report adipocyte density, as above)
**adipose area** (per marrow area)	adipose tissue fraction; fat fraction; bone marrow fat fraction; marrow fat tissue fraction	*none;* FF	%, fraction *(not %)*	1989	4	[36; 346; 429; 477]	**Report as adipose area (Ad.Ar) relative to marrow area (Ma.Ar) or tissue area (T.Ar). This should be presented as % and referred to as 'Adipose area' or 'Adipose tissue area'.** *Don't report absolute adipose area without using a referent area*
	percent adipocyte area; marrow adipose tissue %	*none;* BM	%, % per BM area, % (adipocytes/marrow)	2012	4	[41; 59; 61; 399]	
	adipocyte coverage	BM	% of total BM	2018	1	[393]	
	marrow fat proportion	*none*	% of marrow area	2019	1	[111]	
	metaphyseal adipose area	*none*	% of medullar area	2012	1	[22]	
**adipose area** (per tissue area)	adiposity; marrow adiposity	*none;* Ad.Ar/T.Ar	%	2013	5	[30; 60; 62; 80; 104]	
	adipocyte area; area of fat infiltration; AV/TV	none; AV/TV	%, % tissue area	2001	4	[33; 148; 334; 353]	
	adipocyte area/tissue area	*none;* Ad.Ar/T.Ar	%, fraction *(not %)*	2010	4	[102; 125; 130; 156]	
	fat fraction; vertebral fat	Ad.A/T.A	%	2009	2	[122; 507]	
**adipose area** (per haematopoietic tissue area)	adipocytic tissue/hematopoietic tissue	A/H ratio	*n.s., %*	2002	2	[507; 541]	

**adipose volume** (per haematopoietic marrow volume)	marrow adiposity	AdV/HmMaV	%	1998	1	[3]	**Report as adipose volume (Ad.V) relative to marrow volume (MA.V) or tissue volume (TV). This should be presented as % and referred to as 'Adipose volume' or 'Adipose tissue volume'.** *Don't report absolute adipose volume without using a referent volume*
**adipose volume** (per marrow volume)	adipocyte volume; adipocyte volume/marrow volume; marrow adipose tissue volume	*none;* AV/MV; Ad.V.Ma.V; Ad.V/MV	*n.s.,* %	2000	13	[19; 25; 31; 32; 45; 51; 69; 156; 344; 374; 377; 396; 539]	
	fat marrow volume; bone fat volume as % marrow volume	FV/MV; Fa.V/Ma.V	%	2004	2	[351; 467]	
	BMAT fraction; BMAT volume fraction	AdV/MarV; AD.V/Ma.V	%	2019	2	[103; 485]	
**adipose volume** (per tissue volume)	adipocyte volume/tissue volume; adipose volume/tissue volume; adipose volume/TV	Ad/TV; AV/TV	*n.s.,* %, fraction (*not %*)	1998	8	[3; 14; 43; 50; 106; 116; 347; 401]	
	adipocyte volume; total adipose tissue volume	AV/TV; Ad.V/TV	%	1991	2	[377; 468]	
	adipocyte volume fraction; BMAT volume fraction	AV/TV; Ad.V/TV	%	2016	2	[64; 103]	
	adipose tissue volume per total volume	AV/TV	%	2001	1	[479]	
	marrow adiposity	FV/BV	*%*	2011	1	[13]	
**adipose volume** (absolute)	marrow adipose volume	MAV	*n.s.*	1983	1	[542]	**Don't use** *(instead report as % Ma.V or % TV)*
**adipocyte volume** (absolute)	adipocyte volume	*none*	*n.s.*	1993	1	[477]	**Don't use**

**adipose tissue area** (absolute)	marrow adipose tissue area	*none*	mm^2	2017	1	[87]	**Don't use** (instead report as % Ma.Ar, % Hm.Ar o`r % T.Ar)
**adipose area** (per field or section area)	marrow adiposity; proximal tibia adiposity; bone marrow fat; fat content	*none*	%	1950	4	[15; 172; 543; 544]	
	adipocyte area	Adipo/SA	% (adipocyte area/section area)	2009	1	[504]	
**adipose area** (denominator unclear)	marrow fat; per adipocyte area; percentage of adipocyte area	*none*	pixels, %	2002	8	[24; 86; 94; 96; 308; 350; 382; 409]	
**adipocyte surface** (per trabecular surface)	Fat tissue fraction	*none*	%	2003	1	[260]	**Don't use**
**Other measurements**	unit adipocyte volume	AV/N.A	mcm2	2000	2	[344; 396]	
	adipocyte size/marrow volume	Ad.size/MV	µm	2015	2	[51; 69]	
	Index of proportionality	N/S	adipocyte number/size	1993	1	[477]	
	Unit fat volume	Fa.V/N.Fa	μm^2	2005	1	[351]	

Supplementary Table 6 (related to Table 7): Full list of all terms, abbreviations and units that have been used to refer to histomorphometric measurements related to BMA. Data were obtained and are presented as described for Supplementary Table 1, except that recommendations are also shown here given the greater amount of terms, abbreviations and units that have been used for histological measurements. In many cases, the same abbreviation has been applied to several different terms, whereas sometimes measurements are described without an abbreviation (*none*) and/or units are not stated (*n.s.*). Owing to space limitations, several terms are shown in this table but not in Table 7; it is recommended that these terms no longer be used to report histology-based measurements relating to BMA. **For readouts of adipocyte perimeter (Ad.Pm) and diameter (Ad.Dm), we recommend showing the frequency distribution for each BMAd, rather than the mean or median of all BMAds.

Term	Abbreviation	Units	Year first used*	No. of papers using this term*	References
Fat fraction					
fat fraction	FF	*n.s.*	2004	1	[514]
	FF	%	2015	9	[95; 143; 150; 235; 252; 274; 414; 511; 524]
	FF	lipid/water ratio	2017	2	[311; 480]
	none	%	2001	14	[45; 70; 75; 86; 94; 140; 141; 229; 251; 330; 332; 411; 509; 523]
	none	*n.s.*	1999	1	[301]
marrow fat fraction	FF	%	2015	5	[56; 86; 96; 308; 408]
	FF	lipid/water ratio	2015	1	[545]
	FF	*n.s.*	2013	2	[32; 84]
	MFF	%	2014	3	[41; 70; 401]
	none	*n.s.*	1992	14	[43; 44; 88; 95; 99; 119; 132; 223; 227; 249;
	none	%	2010	2	[94; 259]
bone marrow fat fraction	BM fat fraction	%	*2001*	*5*	*[137; 141; 225; 259; 547]*
	BMF	%	2004	1	[514]
	BMFF	%	2015	2	[46; 180]
	BMFF	*n.s.*	2016	3	[168; 182; 237]
	BMFF	%	2015	1	[232]
	FF	*n.s.*	2002	1	[346]
	none	*n.s.*	1995	8	[140; 216; 227; 245; 271; 329; 481; 524]
	BM fat fraction	*n.s.*	2018	1	[171]
	none	lipid/water ratio	2015	1	[422]
proton density fat fraction	PDFF	%	2014	15	[97; 119; 137; 138; 141; 158; 160; 227; 232;
	PDFF	*n.s.*	2016	2	[237; 256]
	proton density FF	%	2017	1	[84]
	none	*n.s.*	2015	2	[46; 144]

signal fat fraction	sFF	%	2013	2	[223; 385]
	sFF	n.s.	2015	1	[43]
	none	*n.s.*	2012	1	[222]
fat signal fraction	FF	%	2017	1	[310]
	none	%	2012	1	[335]
	none	n.s.	2015	1	[422]
lipid fraction	*none*	*lipid/water ratio*	1985	1	[202]
	none	*%*	2000	2	[183; 550]
bone marrow lipid fraction	LF	%	2012	1	[419]
red marrow fat fraction	*none*	*n.s.*	2015	1	[45]
marrow lipid fraction	*none*	%	2013	1	[31]
marrow fat volume fraction	*none*	%	2015	1	[53]
MAT fraction	MAT fraction	lipid/water ratio	2015	2	[44; 474]
bone marrow proton density fat	PDFF	%	2017	1	[271]
fractional proton density of fat	*none*	n.s.	1985	1	[202]
proportion of protons in the fat	*none*	*%*	2001	1	[330]
vertebral fat fraction	VFF	%	2012	2	[222; 250]
	none	%	2009	1	[9]
vertebral marrow fat fraction	VFF	%	2018	1	[281]
yellow marrow fat fraction	*none*	*n.s.*	2015	1	[45]
Fat content					
fat content	FC	%	2012	3	[255; 267; 529]
	none	%	1987	4	[203; 309; 481; 502]
	none	*n.s.*	1990	8	[179; 211; 219; 299; 310; 375; 511; 550]
bone marrow fat content	BM fat content	*n.s.*	2018	1	[171]
	BMF content	%	2015	3	[98; 309; 367]
	bone marrow FC	%	2016	1	[146]
	FC	%	2012	1	[551]
	none	%	2011	3	[335; 506; 521]
	none	lipid/water ratio	2009	2	[12; 354]
marrow fat content	*none*	%	2005	7	[401; 501; 502; 505; 506; 546; 552]
	none	L	2007	1	[482]
	none	% fat fraction	2015	1	[509]
vertebral bone marrow fat content	VBM fat content	%	2017	1	[79]

fractional fat content	*none*	*n.s.*	1985	1	[202]
	none	%	2017	1	[241]
marrow adipose tissue content	MAT content	%	2016	1	[403]
	MAT content	lipid/water ratio	2018	1	[253]
bone marrow adipose tissue	BMAT content	*n.s.*	2018	1	[171]
Marrow fat					
bone marrow fat	BM fat	%	2017	1	[423]
	BMF	%	2014	2	[230; 407]
	l/w	lipid/water ratio	2014	1	[553]
	none	%	2011	6	[16; 35; 81; 99; 309; 520]
bone marrow fat composition	*none*	*n.s.*	2019	1	[276]
bone marrow fat concentration	*none*	%	2017	1	[243]
fat marrow	*none*	%	2006	1	[219]
intravertebral bone marrow fat	*none*	%	2002	1	[528]
marrow fat	*none*	%	2009	4	[81; 131; 421; 554]
	none	*n.s.*	1984	2	[294; 555]
marrow fat composition	*none*	*n.s.*	2014	1	[508]
marrow fat quantity	*none*	%	2015	1	[45]
vertebral marrow fat	*none*	%	2013	1	[35]
Bone marrow adipose tissue					
bone marrow adipose tissue	BMAT	L	2012	6	[225; 304; 305; 366; 420; 433]
bone marrow adipose tissue volume	BMAT volume	cm^3	2011	2	[220; 483]
marrow adipose tissue	MAT	%	2018	1	[411]
	MAT	lipid/water ratio	2014	3	[71; 385; 556]
red marrow adipose tissue	rMAT	%	2018	1	[410]
yellow marrow adipose tissue	yMAT	%	2018	1	[410]
Ratio (fat/water or lipid/water)					
bone marrow fat/water %	*none*	%	2011	1	[516]
bone marrow fat/water ratio	*none*	%	2014	1	[279]
fat/water ratio	*none*	*none*	2014	1	[279]
lipid/water ratio	l/w	*none*	2014	1	[508]
	LWR	*none*	2004	3	[16; 21; 514]
	none	%	2011	2	[516; 553]

	none	*none*	2013	7	[28; 71; 149; 306; 414; 474; 475]
Area					
bone marrow adipose tissue area	BMA	cm^2	2015	1	[233]
bone marrow adiposity cross-	CSA	cm^2	2017	1	[270]
yellow bone marrow cross-sectional	CSA	mm^2	2013	1	[175]
Other terms					
bone marrow adiposity	*none*	%	2012	1	[27]
fat component	*none*	n.s.	1999	1	[301]
fat concentration	*none*	n.s.	2000	1	[550]
fat proportion	*none*	%	2001	1	[547]
fat signal	*none*	n.s.	1993	1	[557]
fat signal intensity	*none*	n.s.	1995	2	[212; 330]
index of marrow conversion	IMC	n.s.	2006	1	[219]
percent marrow fat	%MF	%	2011	1	[131]
percentage of fat marrow	*none*	%	1999	1	[215]
proportion of fat	*none*	%	2001	1	[330]
spine fat	*none*	%	2011	1	[16]

Supplementary Table 7 (related to Table 8): Full list of all combinations of terms, abbreviations and units that have been used to report MRI/MRS-based measurements of bone marrow adiposity. Data were obtained and are presented as described for Supplementary Table 1. In some cases, no abbreviation is used (*none*) and/or units are not stated (*n.s.*). Recommended terms and abbreviations are presented in Table 8. Several terms are shown in this table but, because of space limitations, these have not been shown in Table 8; it is recommended that these terms no longer be used to report MRI/MRS-based measurements relating to BMA.

Imaging method	Measurement type	Described as	Abbreviations used	Units used	Year first used*	No. of papers*	References
***SECT, DECT or µCT** (without contrast agent)*	**Fat fraction** (% or fraction)	fat content; bone marrow fat content	*none*	%	1987	3	[498; 527; 558]
		yellow marrow fraction; marrow fat	YMF; MF	fraction; %	2015	3	[43; 50; 559]
		MAT fraction	MAT fraction	fraction	2015	1	[44]
		fractional volume of yellow marrow	YM	fraction	2019	1	[256]
	**Adipose volume** (per marrow volume)	adipose volume to total marrow volume	AV/TV	%	2019	1	[118]
		marrow adiposity index	MAI	%	2019	1	[118]
		BMAT	Ad.V/Ma.V	%	2019	1	[257]
	**BM density**	CT density	*none*	K**	1986	1	[496]
		Fat density; marrow fat density; CT marrow density	Marrow FD; MaD	g/cm^3, mg/cm^3	2008	4	[4; 5; 11; 34]
		Bone marrow density	*none*	mg/cm^3	2019	1	[105]
	**adipose volume** (per tissue volume)	marrow adiposity; marrow adipose tissue % (MAT %)	FV/BV, FV/TV, MAT	%	2011	2	[13; 89]
***µCT** (samples stained with osmium tetroxide or other contrast agents)*	**adipose volume** (per marrow volume)	adipocyte volume/marrow volume	*none*, Ad.V/Ma.V	%	2014	4	[91; 136; 182; 257]
		marrow adipose tissue/marrow volume; marrow fat/marrow volume	*none*, MAT/marrow	%	2014	4	[39; 76; 234; 318]
		metaphyseal adipose area	*none*	% medullar area	2012	*1*	[22]
		marrow adipose tissue (MAT)	MAT, MV	%MV	2014	1	[385]
		fat volume/total volume	FV/TV	fraction	2017	1	[242]
	**adipose volume** (per tissue or total volume)	adipocyte volume/total volume	AV/TV	fraction	2014	2	[38; 400]
		MAT volume/total volume; marrow	*none*, MAT/TV	%	2015	3	[145; 472; 487]
		adipose/tissue volume; vol. adipose	*none*	%	2017	2	[77; 102]
		MAT volume/femoral volume	MAT	%	2017	1	[406]
		MAT volume/tissue volume	MAT/TV	%	2016	1	[405]

		percentage of adipocyte volume in the total volume	Ad.V/TV	%	2018	1	[91]
		% osmium in mm^3/total bone volume	*none*	%	2014	1	[471]
		osmium volume/marrow cavity volume	OV/TV	*n.s.*	2016	1	[68]
	**adipose volume** (unclear if per tissue volume or per marrow volume)	bone marrow fat	*none*	*n.s.*	2017	1	[152]
		marrow fat fraction	*none*	fraction	2016	1	[404]
		marrow adipose tissue (MAT)	MAT	% osmium	2015	1	[473]
		percent adiposity	*none*	%	2017	1	[85]
	**adipose volume** (absolute)	MAT volume	MAT, MAT vol.	mm^3	2015	4	[76; 234; 370; 412]
		distal MAT volume; proximal MAT	*none*, dTib	mm^3	2016	3	[93; 405; 412]
		osmium volume	*none*	mm^3	2016	2	[68; 327]
		tibia BMAT volume; proximal tibia	*BMAT*	mm^3	2018	1	[172]
		marrow fat volume; distal fat volume; proximal fat volume	*FV, dFV, pFV*	mm^3	2017	1	[242]
	**adipocyte density** (per marrow volume)	adipocyte number; adipocyte density	Ad.N	mm^{-3}	2014	2	[136; 560]
	**adipocyte area** (distribution of individual adipocyte areas)	adipocyte area	*none*	µm^2	2015	1	[368]

Supplementary Table 8 (related to Table 9): Full list of all terms, abbreviations and units that have been used to report CT-based measurements of bone marrow adiposity. Terms are grouped by imaging method and the type of measurement reported. Data are presented as described for Supplementary Table 6. Most studies assess adipose volume normalized to marrow volume or total tissue volume, although in some cases it is unclear which of these is used for normalisation. In some cases, no abbreviation is used (*none*) and/or units are not stated (*n.s.*). **K = density relative to K_2HPO_4 phantom.

References:

[1] N. Gengozian, J.S. Batson, and B.M. Nelson, Bone-marrow grafting attempts in marmosets after whole-body irradiation. Int. J. Radiat. Biol. Relat. Stud. Phys. Chem. Med. 11 (1967) 553-61.

[2] C.M. Schnitzler, S.L. Biddulph, J.M. Mesquita, and K.A. Gear, Bone structure and turnover in the distal radius and iliac crest: A histomorphometric study. J. Bone Miner. Res. 11 (1996) 1761-1768.

[3] C.M. Schnitzler, and J. Mesquita, Bone marrow composition and bone microarchitecture and turnover in blacks and whites. J. Bone Miner. Res. 13 (1998) 1300-7.

[4] N. Di Iorgi, S.D. Mittelman, and V. Gilsanz, Differential effect of marrow adiposity and visceral and subcutaneous fat on cardiovascular risk in young, healthy adults. Int J Obes (Lond) 32 (2008) 1854-60.

[5] N. Di Iorgi, M. Rosol, S.D. Mittelman, and V. Gilsanz, Reciprocal relation between marrow adiposity and the amount of bone in the axial and appendicular skeleton of young adults. J. Clin. Endocrinol. Metab. 93 (2008) 2281-6.

[6] C.L. Ackert-Bicknell, K.R. Shockley, L.G. Horton, B. Lecka-Czernik, G.A. Churchill, and C.J. Rosen, Strain-specific effects of rosiglitazone on bone mass, body composition, and serum insulin-like growth factor-I. Endocrinology 150 (2009) 1330-40.

[7] L.R. McCabe, Switching fat from the periphery to bone marrow: why in Type I diabetes? Expert review of endocrinology & metabolism 4 (2009) 203-207.

[8] C.J. Rosen, C. Ackert-Bicknell, J.P. Rodriguez, and A.M. Pino, Marrow fat and the bone microenvironment: developmental, functional, and pathological implications. Crit. Rev. Eukaryot. Gene Expr. 19 (2009) 109-24.

[9] G. Trudel, M. Payne, B. Madler, N. Ramachandran, M. Lecompte, C. Wade, G. Biolo, S. Blanc, R. Hughson, L. Bear, and H.K. Uhthoff, Bone marrow fat accumulation after 60 days of bed rest persisted 1 year after activities were resumed along with hemopoietic stimulation: the Women International Space Simulation for Exploration study. J Appl Physiol (1985) 107 (2009) 540-8.

[10] M.J. Devlin, A.M. Cloutier, N.A. Thomas, D.A. Panus, S. Lotinun, I. Pinz, R. Baron, C.J. Rosen, and M.L. Bouxsein, Caloric restriction leads to high marrow adiposity and low bone mass in growing mice. J. Bone Miner. Res. 25 (2010) 2078-88.

[11] N. Di Iorgi, A.O. Mo, K. Grimm, T.A. Wren, F. Dorey, and V. Gilsanz, Bone acquisition in healthy young females is reciprocally related to marrow adiposity. The Journal of clinical endocrinology and metabolism 95 (2010) 2977-82.

[12] P.K. Fazeli, M.A. Bredella, M. Misra, E. Meenaghan, C.J. Rosen, D.R. Clemmons, A. Breggia, K.K. Miller, and A. Klibanski, Preadipocyte factor-1 is associated with marrow adiposity and bone mineral density in women with anorexia nervosa. J. Clin. Endocrinol. Metab. 95 (2010) 407-13.

[13] O. Demontiero, W. Li, E. Thembani, and G. Duque, Validation of noninvasive quantification of bone marrow fat volume with microCT in aging rats. Exp. Gerontol. 46 (2011) 435-40.

[14] G. Duque, W. Li, M. Adams, S. Xu, and R. Phipps, Effects of risedronate on bone marrow adipocytes in postmenopausal women. Osteoporos. Int. 22 (2011) 1547-53.

[15] B.A. Evans, M.J. Bull, R.C. Kench, R.E. Fox, L.D. Morgan, A.E. Stevenson, E.F. Gevers, M.J. Perry, and T. Wells, The influence of leptin on trabecular architecture and marrow adiposity in GH-deficient rats. J. Endocrinol. 208 (2011) 69-79.

[16] T. Harslof, L. Wamberg, L. Moller, H. Stodkilde-Jorgensen, S. Ringgaard, S.B. Pedersen, and B.L. Langdahl, Rosiglitazone decreases bone mass and bone marrow fat. The Journal of clinical endocrinology and metabolism 96 (2011) 1541-8.

[17] K.J. Motyl, M. Raetz, S.A. Tekalur, R.C. Schwartz, and L.R. McCabe, CCAAT/enhancer binding protein beta-deficiency enhances type 1 diabetic bone phenotype by increasing marrow adiposity and bone resorption. American journal of physiology. Regulatory, integrative and comparative physiology 300 (2011) R1250-60.

[18] K. Baek, and S.A. Bloomfield, Blocking beta-adrenergic signaling attenuates reductions in circulating leptin, cancellous bone mass, and marrow adiposity seen with dietary energy restriction. J Appl Physiol (1985) 113 (2012) 1792-801.

[19] A. Cohen, D.W. Dempster, E.M. Stein, T.L. Nickolas, H. Zhou, D.J. McMahon, R. Muller, T. Kohler, A. Zwahlen, J.M. Lappe, P. Young, R.R. Recker, and E. Shane, Increased marrow adiposity in premenopausal women with idiopathic osteoporosis. J. Clin. Endocrinol. Metab. 97 (2012) 2782-91.

[20] C.M. Fan, B.K. Foster, S.K. Hui, and C.J. Xian, Prevention of bone growth defects, increased bone resorption and marrow adiposity with folinic acid in rats receiving long-term methotrexate. PLoS One 7 (2012) e46915.

[21] P.K. Fazeli, M.A. Bredella, L. Freedman, B.J. Thomas, A. Breggia, E. Meenaghan, C.J. Rosen, and A. Klibanski, Marrow fat and preadipocyte factor-1 levels decrease with recovery in women with anorexia nervosa. J. Bone Miner. Res. (2012).

[22] C. Fournier, A. Perrier, M. Thomas, N. Laroche, V. Dumas, A. Rattner, L. Vico, and A. Guignandon, Reduction by strontium of the bone marrow adiposity in mice and repression of the adipogenic commitment of multipotent C3H10T1/2 cells. Bone 50 (2012) 499-509.

[23] K.R. Georgiou, T.J. King, M.A. Scherer, H. Zhou, B.K. Foster, and C.J. Xian, Attenuated Wnt/beta-catenin signalling mediates methotrexate chemotherapy-induced bone loss and marrow adiposity in rats. Bone 50 (2012) 1223-33.

[24] S.K. Hui, L. Sharkey, L.S. Kidder, Y. Zhang, G. Fairchild, K. Coghill, C.J. Xian, and D. Yee, The influence of therapeutic radiation on the patterns of bone marrow in ovary-intact and ovariectomized mice. PLoS One 7 (2012) e42668.

[25] J.Y. Ko, R.W. Wu, S.J. Kuo, M.W. Chen, D.W. Yeh, H.C. Ke, S.L. Wu, and F.S. Wang, Cannabinoid receptor 1 mediates glucocorticoid-induced bone loss in rats by perturbing bone mineral acquisition and marrow adipogenesis. Arthritis Rheum. 64 (2012) 1204-14.

[26] H. Sadie-Van Gijsen, N.J. Crowther, F.S. Hough, and W.F. Ferris, The interrelationship between bone and fat: from cellular see-saw to endocrine reciprocity. Cellular and molecular life sciences : CMLS (2012).

[27] J.M. Slade, L.M. Coe, R.A. Meyer, and L.R. McCabe, Human bone marrow adiposity is linked with serum lipid levels not T1-diabetes. J. Diabetes Complications 26 (2012) 1-9.

[28] M.A. Bredella, C.M. Gill, A.V. Gerweck, M.G. Landa, V. Kumar, S.M. Daley, M. Torriani, and K.K. Miller, Ectopic and Serum Lipid Levels Are Positively Associated with Bone Marrow Fat in Obesity. Radiology (2013).

[29] M.K. Herroon, E. Rajagurubandara, A.L. Hardaway, K. Powell, A. Turchick, D. Feldmann, and I. Podgorski, Bone marrow adipocytes promote tumor growth in bone via FABP4-dependent mechanisms. Oncotarget 4 (2013) 2108-23.

[30] U.T. Iwaniec, and R.T. Turner, Failure to generate bone marrow adipocytes does not protect mice from ovariectomy-induced osteopenia. Bone 53 (2013) 145-53.

[31] G.W. Li, Z. Xu, Q.W. Chen, S.X. Chang, Y.N. Tian, and J.Z. Fan, The temporal characterization of marrow lipids and adipocytes in a rabbit model of glucocorticoid-induced osteoporosis. Skeletal Radiol. 42 (2013) 1235-44.

[32] G.W. Li, S.X. Chang, J.Z. Fan, Y.N. Tian, Z. Xu, and Y.M. He, Marrow adiposity recovery after early zoledronic acid treatment of glucocorticoid-induced bone loss in rabbits assessed by magnetic resonance spectroscopy. Bone 52 (2013) 668-75.

[33] L.F. Liu, W.J. Shen, M. Ueno, S. Patel, S. Azhar, and F.B. Kraemer, Age-related modulation of the effects of obesity on gene expression profiles of mouse bone marrow and epididymal adipocytes. PLoS One 8 (2013) e72367.

[34] T. Rantalainen, R. Nikander, A. Heinonen, T. Cervinka, H. Sievanen, and R.M. Daly, Differential effects of exercise on tibial shaft marrow density in young female athletes. J. Clin. Endocrinol. Metab. 98 (2013) 2037-44.

[35] A.V. Schwartz, S. Sigurdsson, T.F. Hue, T.F. Lang, T.B. Harris, C.J. Rosen, E. Vittinghoff, K. Siggeirsdottir, G. Sigurdsson, D. Oskarsdottir, K. Shet, L. Palermo, V. Gudnason, and X. Li, Vertebral Bone Marrow Fat Associated With Lower Trabecular BMD and Prevalent Vertebral Fracture in Older Adults. J. Clin. Endocrinol. Metab. 98 (2013) 2294-300.

[36] B.J. Adler, D.E. Green, G.M. Pagnotti, M.E. Chan, and C.T. Rubin, High fat diet rapidly suppresses B lymphopoiesis by disrupting the supportive capacity of the bone marrow niche. PLoS One 9 (2014) e90639.

[37] K. Baek, H.J. Park, H.R. Hwang, and J.H. Baek, Propranolol attenuates calorie restriction- and high calorie diet-induced bone marrow adiposity. BMB reports 47 (2014) 587-92.

[38] S. Bornstein, S.A. Brown, P.T. Le, X. Wang, V. DeMambro, M.C. Horowitz, O. MacDougald, R. Baron, S. Lotinun, G. Karsenty, W. Wei, M. Ferron, C.S. Kovacs, D. Clemmons, Y. Wan, and C.J. Rosen, FGF-21 and Skeletal Remodeling During and After Lactation in C57BL6 Mice. Endocrinology (2014) en20141083.

[39] M.J. Devlin, M. Van Vliet, K. Motyl, L. Karim, D.J. Brooks, L. Louis, C. Conlon, C.J. Rosen, and M.L. Bouxsein, Early-onset type 2 diabetes impairs skeletal acquisition in the male TALLYHO/JngJ mouse. Endocrinology 155 (2014) 3806-16.

[40] M. Hu, J. Sheng, Z. Kang, L. Zou, J. Guo, and P. Sun, Magnetic resonance imaging and dual energy X-ray absorptiometry of the lumbar spine in professional wrestlers and untrained men. J. Sports Med. Phys. Fitness 54 (2014) 505-10.

[41] G.W. Li, Z. Xu, S.X. Chang, L. Zhou, X.Y. Wang, H. Nian, and X. Shi, Influence of early zoledronic acid administration on bone marrow fat in ovariectomized rats. Endocrinology 155 (2014) 4731-8.

[42] S. Nallamshetty, P.T. Le, H. Wang, M.J. Issacsohn, D.J. Reeder, E.J. Rhee, F.W. Kiefer, J.D. Brown, C.J. Rosen, and J. Plutzky, Retinaldehyde dehydrogenase 1 deficiency inhibits PPARgamma-mediated bone loss and marrow adiposity. Bone 67 (2014) 281-91.

[43] L. Arentsen, M. Yagi, Y. Takahashi, P.J. Bolan, M. White, D. Yee, and S. Hui, Validation of marrow fat assessment using noninvasive imaging with histologic examination of human bone samples. Bone 72 (2015) 118-22.

[44] M.A. Bredella, S.M. Daley, M.K. Kalra, J.K. Brown, K.K. Miller, and M. Torriani, Marrow Adipose Tissue Quantification of the Lumbar Spine by Using Dual-Energy CT and Single-Voxel (1)H MR Spectroscopy: A Feasibility Study. Radiology 277 (2015) 230-5.

[45] A. Cohen, W. Shen, D.W. Dempster, H. Zhou, R.R. Recker, J.M. Lappe, A. Kepley, M. Kamanda-Kosseh, M. Bucovsky, E.M. Stein, T.L. Nickolas, and E. Shane, Marrow adiposity assessed on transiliac crest biopsy samples correlates with noninvasive measurement of marrow adiposity by proton magnetic resonance spectroscopy ((1)H-MRS) at the spine but not the femur. Osteoporos. Int. 26 (2015) 2471-8.

[46] C. Cordes, M. Dieckmeyer, B. Ott, J. Shen, S. Ruschke, M. Settles, C. Eichhorn, J.S. Bauer, H. Kooijman, E.J. Rummeny, T. Skurk, T. Baum, H. Hauner, and D.C. Karampinos, MR-detected changes in liver fat, abdominal fat, and vertebral bone marrow fat after a four-week calorie restriction in obese women. J. Magn. Reson. Imaging 42 (2015) 1272-80.

[47] M.J. Devlin, and C.J. Rosen, The bone-fat interface: basic and clinical implications of marrow adiposity. The lancet. Diabetes & endocrinology 3 (2015) 141-147.

[48] K.R. Georgiou, R.R. Nadhanan, C.M. Fan, and C.J. Xian, Methotrexate-induced bone marrow adiposity is mitigated by folinic acid supplementation through the regulation of Wnt/beta-catenin signalling. J. Cell. Physiol. 230 (2015) 648-56.

[49] A.L. Hardaway, M.K. Herroon, E. Rajagurubandara, and I. Podgorski, Marrow adipocyte-derived CXCL1 and CXCL2 contribute to osteolysis in metastatic prostate cancer. Clin. Exp. Metastasis 32 (2015) 353-68.

[50] S.K. Hui, L. Arentsen, T. Sueblinvong, K. Brown, P. Bolan, R.G. Ghebre, L. Downs, R. Shanley, K.E. Hansen, A.G. Minenko, Y. Takhashi, M. Yagi, Y. Zhang, M. Geller, M. Reynolds, C.K. Lee, A.H. Blaes, S. Allen, B.B. Zobel, C. Le, J. Froelich, C. Rosen, and D. Yee, A phase I feasibility study of multi-modality imaging assessing rapid expansion of marrow fat and decreased bone mineral density in cancer patients. Bone 73 (2015) 90-7.

[51] J.Y. Ko, P.C. Chuang, H.J. Ke, Y.S. Chen, Y.C. Sun, and F.S. Wang, MicroRNA-29a mitigates glucocorticoid induction of bone loss and fatty marrow by rescuing Runx2 acetylation. Bone 81 (2015) 80-88.

[52] P.J. Martin, N. Haren, O. Ghali, A. Clabaut, C. Chauveau, P. Hardouin, and O. Broux, Adipogenic RNAs are transferred in osteoblasts via bone marrow adipocytes-derived extracellular vesicles (EVs). BMC cell biology 16 (2015) 10.

[53] S. Mostoufi-Moab, J. Magland, E.J. Isaacoff, W. Sun, C.S. Rajapakse, B. Zemel, F. Wehrli, K. Shekdar, J. Baker, J. Long, and M.B. Leonard, Adverse Fat Depots and Marrow Adiposity Are Associated With Skeletal Deficits and Insulin Resistance in Long-Term Survivors of Pediatric Hematopoietic Stem Cell Transplantation. J. Bone Miner. Res. 30 (2015) 1657-66.

[54] S. Periyasamy-Thandavan, S. Herberg, P. Arounleut, S. Upadhyay, A. Dukes, C. Davis, M. Johnson, M. McGee-Lawrence, M.W. Hamrick, C.M. Isales, and W.D. Hill, Caloric

restriction and the adipokine leptin alter the SDF-1 signaling axis in bone marrow and in bone marrow derived mesenchymal stem cells. Mol. Cell. Endocrinol. 410 (2015) 64-72.

[55] A.V. Schwartz, Marrow fat and bone: review of clinical findings. Front Endocrinol (Lausanne) 6 (2015) 40.

[56] Y. Yang, X. Luo, F. Yan, Z. Jiang, Y. Li, C. Fang, and J. Shen, Effect of zoledronic acid on vertebral marrow adiposity in postmenopausal osteoporosis assessed by MR spectroscopy. Skeletal Radiol. 44 (2015) 1499-505.

[57] C.J. Cain, J.T. Valencia, S. Ho, K. Jordan, A. Mattingly, B.M. Morales, and E.C. Hsiao, Increased Gs Signaling in Osteoblasts Reduces Bone Marrow and Whole-Body Adiposity in Male Mice. Endocrinology 157 (2016) 1481-94.

[58] H. Chkourko Gusky, J. Diedrich, O.A. MacDougald, and I. Podgorski, Omentum and bone marrow: how adipocyte-rich organs create tumour microenvironments conducive for metastatic progression. Obesity reviews : an official journal of the International Association for the Study of Obesity 17 (2016) 1015-1029.

[59] C. Fan, K.R. Georgiou, R.A. McKinnon, D.M. Keefe, P.R. Howe, and C.J. Xian, Combination chemotherapy with cyclophosphamide, epirubicin and 5-fluorouracil causes trabecular bone loss, bone marrow cell depletion and marrow adiposity in female rats. J. Bone Miner. Metab. 34 (2016) 277-90.

[60] U.T. Iwaniec, K.A. Philbrick, C.P. Wong, J.L. Gordon, A.M. Kahler-Quesada, D.A. Olson, A.J. Branscum, J.L. Sargent, V.E. DeMambro, C.J. Rosen, and R.T. Turner, Room temperature housing results in premature cancellous bone loss in growing female mice: implications for the mouse as a preclinical model for age-related bone loss. Osteoporos. Int. (2016).

[61] G. Li, Z. Xu, L. Hou, X. Li, X. Li, W. Yuan, M. Polat, and S. Chang, Differential effects of bisphenol A diglicydyl ether on bone quality and marrow adiposity in ovary-intact and ovariectomized rats. Am. J. Physiol. Endocrinol. Metab. 311 (2016) E922-e927.

[62] L.B. Lindenmaier, K.A. Philbrick, A.J. Branscum, S.P. Kalra, R.T. Turner, and U.T. Iwaniec, Hypothalamic Leptin Gene Therapy Reduces Bone Marrow Adiposity in ob/ob Mice Fed Regular and High-Fat Diets. Front Endocrinol (Lausanne) 7 (2016) 110.

[63] W. Lu, W. Wang, S. Wang, Y. Feng, and K. Liu, Rosiglitazone Promotes Bone Marrow Adipogenesis to Impair Myelopoiesis under Stress. PLoS One 11 (2016) e0149543.

[64] M.E. McGee-Lawrence, L.R. Carpio, R.J. Schulze, J.L. Pierce, M.A. McNiven, J.N. Farr, S. Khosla, M.J. Oursler, and J.J. Westendorf, Hdac3 Deficiency Increases Marrow Adiposity and Induces Lipid Storage and Glucocorticoid Metabolism in Osteochondroprogenitor Cells. J. Bone Miner. Res. 31 (2016) 116-28.

[65] E.V. Morris, and C.M. Edwards, Bone Marrow Adipose Tissue: A New Player in Cancer Metastasis to Bone. Front Endocrinol (Lausanne) 7 (2016) 90.

[66] G.M. Pagnotti, and M. Styner, Exercise Regulation of Marrow Adipose Tissue. Front Endocrinol (Lausanne) 7 (2016) 94.

[67] E.L. Scheller, B. Khoury, K.L. Moller, N.K. Wee, S. Khandaker, K.M. Kozloff, S.H. Abrishami, B.F. Zamarron, and K. Singer, Changes in Skeletal Integrity and Marrow Adiposity during High-Fat Diet and after Weight Loss. Front Endocrinol (Lausanne) 7 (2016) 102.

[68] T.A. Walji, S.E. Turecamo, A.C. Sanchez, B.A. Anthony, G. Abou-Ezzi, E.L. Scheller, D.C. Link, R.P. Mecham, and C.S. Craft, Marrow Adipose Tissue Expansion Coincides with Insulin Resistance in MAGP1-Deficient Mice. Front Endocrinol (Lausanne) 7 (2016) 87.

[69] F.S. Wang, W.S. Lian, W.T. Weng, Y.C. Sun, H.J. Ke, Y.S. Chen, and J.Y. Ko, Neuropeptide Y mediates glucocorticoid-induced osteoporosis and marrow adiposity in mice. Osteoporos. Int. 27 (2016) 2777-2789.

[70] Y. Yang, X. Luo, X. Xie, F. Yan, G. Chen, W. Zhao, Z. Jiang, C. Fang, and J. Shen, Influences of teriparatide administration on marrow fat content in postmenopausal osteopenic women using MR spectroscopy. Climacteric 19 (2016) 285-91.

[71] E.W. Yu, L. Greenblatt, A. Eajazi, M. Torriani, and M.A. Bredella, Marrow adipose tissue composition in adults with morbid obesity. Bone 97 (2016) 38-42.

[72] R. Yue, B.O. Zhou, I.S. Shimada, Z. Zhao, and S.J. Morrison, Leptin Receptor Promotes Adipogenesis and Reduces Osteogenesis by Regulating Mesenchymal Stromal Cells in Adult Bone Marrow. Cell Stem Cell 18 (2016) 782-96.

[73] T.H. Ambrosi, and T.J. Schulz, The emerging role of bone marrow adipose tissue in bone health and dysfunction. J Mol Med (Berl) 95 (2017) 1291-1301.

[74] A. Bartelt, T. Koehne, K. Todter, R. Reimer, B. Muller, F. Behler-Janbeck, J. Heeren, L. Scheja, and A. Niemeier, Quantification of Bone Fatty Acid Metabolism and Its Regulation by Adipocyte Lipoprotein Lipase. Int J Mol Sci 18 (2017).

[75] C.M. Bastos, I.M. Araujo, M.H. Nogueira-Barbosa, C.E.G. Salmon, F.J.A. de Paula, and L.E.A. Troncon, Reduced bone mass and preserved marrow adipose tissue in patients with inflammatory bowel diseases in long-term remission. Osteoporos. Int. 28 (2017) 2167-2176.

[76] S. Bornstein, M. Moschetta, Y. Kawano, A. Sacco, D. Huynh, D. Brooks, S. Manier, H. Fairfield, C. Falank, A.M. Roccaro, K. Nagano, R. Baron, M. Bouxein, C. Vary, I.M. Ghobrial, C.J. Rosen, and M.R. Reagan, Metformin Affects Cortical Bone Mass and Marrow Adiposity in Diet-Induced Obesity in Male Mice. Endocrinology 158 (2017) 3369-3385.

[77] Y. Fan, J.I. Hanai, P.T. Le, R. Bi, D. Maridas, V. DeMambro, C.A. Figueroa, S. Kir, X. Zhou, M. Mannstadt, R. Baron, R.T. Bronson, M.C. Horowitz, J.Y. Wu, J.P. Bilezikian, D.W. Dempster, C.J. Rosen, and B. Lanske, Parathyroid Hormone Directs Bone Marrow Mesenchymal Cell Fate. Cell Metab. (2017).

[78] M.C. Horowitz, R. Berry, B. Holtrup, Z. Sebo, T. Nelson, J.A. Fretz, D. Lindskog, J.L. Kaplan, G. Ables, M.S. Rodeheffer, and C.J. Rosen, Bone marrow adipocytes. Adipocyte (2017) 1-12.

[79] K.K. Ivaska, V. Huovinen, M. Soinio, J.C. Hannukainen, V. Saunavaara, P. Salminen, M. Helmio, R. Parkkola, P. Nuutila, and R. Kiviranta, Changes in bone metabolism after bariatric surgery by gastric bypass or sleeve gastrectomy. Bone 95 (2017) 47-54.

[80] J.A. Keune, C.P. Wong, A.J. Branscum, U.T. Iwaniec, and R.T. Turner, Bone Marrow Adipose Tissue Deficiency Increases Disuse-Induced Bone Loss in Male Mice. Scientific reports 7 (2017) 46325.

[81] T.Y. Kim, A.V. Schwartz, X. Li, K. Xu, D.M. Black, D.M. Petrenko, L. Stewart, S.J. Rogers, A.M. Posselt, J.T. Carter, D.M. Shoback, and A.L. Schafer, Bone Marrow Fat Changes After Gastric Bypass Surgery Are Associated With Loss of Bone Mass. J. Bone Miner. Res. (2017).

[82] B. Lanske, and C. Rosen, Bone Marrow Adipose Tissue: The First 40 Years. J. Bone Miner. Res. 32 (2017) 1153-1156.

[83] X. Li, K. Shet, K. Xu, J.P. Rodriguez, A.M. Pino, J. Kurhanewicz, A. Schwartz, and C.J. Rosen, Unsaturation level decreased in bone marrow fat of postmenopausal women with low bone density using high resolution magic angle spinning (HRMAS) 1H NMR spectroscopy. Bone 105 (2017) 87-92.

[84] G. Li, Z. Xu, A. Zhuang, S. Chang, L. Hou, Y. Chen, M. Polat, and D. Wu, Magnetic Resonance Spectroscopy-Detected Change in Marrow Adiposity Is Strongly Correlated to Postmenopausal Breast Cancer Risk. Clin Breast Cancer 17 (2017) 239-244.

[85] R.C. Lindsey, and S. Mohan, Thyroid hormone acting via TRbeta induces expression of browning genes in mouse bone marrow adipose tissue. Endocrine 56 (2017) 109-120.

[86] Y. Liu, S. Wu, R. Cui, Q. Chan, S. Zhang, Y. Liao, and C. Huang, Proton Magnetic Resonance Spectroscopy-Detected Changes of Marrow Fat Content in a Rabbit Model of Osteoporosis Treated With Epigallocatechin-3-Gallate. J. Comput. Assist. Tomogr. 41 (2017) 231-235.

[87] G. Trudel, H.K. Uhthoff, S. Solanki, and O. Laneuville, The effects of knee immobilization on marrow adipocyte hyperplasia and hypertrophy at the proximal rat tibia epiphysis. Acta Histochem. 119 (2017) 759-765.

[88] A.G. Veldhuis-Vlug, and C.J. Rosen, Mechanisms of marrow adiposity and its implications for skeletal health. Metabolism. 67 (2017) 106-114.

[89] E. Bani Hassan, O. Demontiero, S. Vogrin, A. Ng, and G. Duque, Marrow Adipose Tissue in Older Men: Association with Visceral and Subcutaneous Fat, Bone Volume, Metabolism, and Inflammation. Calcif. Tissue Int. 103 (2018) 164-174.

[90] C.A. Bradley, Bone: PPARgamma controls marrow adiposity. Nature reviews. Endocrinology 14 (2018) 3.

[91] X. Coutel, C. Olejnik, P. Marchandise, J. Delattre, H. Behal, G. Kerckhofs, and G. Penel, A Novel microCT Method for Bone and Marrow Adipose Tissue Alignment Identifies Key Differences Between Mandible and Tibia in Rats. Calcif. Tissue Int. 103 (2018) 189-197.

[92] K.A. Fader, R. Nault, S. Raehtz, L.R. McCabe, and T.R. Zacharewski, 2,3,7,8-Tetrachlorodibenzo-p-dioxin dose-dependently increases bone mass and decreases marrow adiposity in juvenile mice. Toxicol. Appl. Pharmacol. 348 (2018) 85-98.

[93] H. Fairfield, C. Falank, E. Harris, V. Demambro, M. McDonald, J.A. Pettitt, S.T. Mohanty, P. Croucher, I. Kramer, M. Kneissel, C.J. Rosen, and M.R. Reagan, The skeletal cell-derived molecule sclerostin drives bone marrow adipogenesis. J. Cell. Physiol. 233 (2018) 1156-1167.

[94] S. Li, H. Jiang, B. Wang, M. Gu, N. Zhang, W. Liang, and Y. Wang, Effect of Leptin on Marrow Adiposity in Ovariectomized Rabbits Assessed by Proton Magnetic Resonance Spectroscopy. J. Comput. Assist. Tomogr. 42 (2018) 588-593.

[95] S. Li, H. Jiang, B. Wang, M. Gu, X. Bi, Y. Yin, and Y. Wang, Magnetic Resonance Spectroscopy for Evaluating the Effect of Pulsed Electromagnetic Fields on Marrow Adiposity in Postmenopausal Women With Osteopenia. J. Comput. Assist. Tomogr. 42 (2018) 792-797.

[96] H. Lin, M. Zheng, X. Mao, X. Feng, J. Li, G. Rao, and F. Lin, Oxytocin treatment prevents marrow adiposity observed in alloxan-induced diabetic rabbits using proton MR spectroscopy. Endokrynol. Pol. 69 (2018).

[97] D. Martel, B. Leporq, M. Bruno, R.R. Regatte, S. Honig, and G. Chang, Chemical shift-encoded MRI for assessment of bone marrow adipose tissue fat composition: Pilot study in premenopausal versus postmenopausal women. Magn. Reson. Imaging 53 (2018) 148-155.

[98] F. Maurice, A. Dutour, C. Vincentelli, I. Abdesselam, M. Bernard, H. Dufour, Y. Lefur, T. Graillon, F. Kober, P. Cristofari, E. Jouve, L. Pini, R. Fernandez, C. Chagnaud, T. Brue, F. Castinetti, and B. Gaborit, Active cushing syndrome patients have increased ectopic fat deposition and bone marrow fat content compared to cured patients and healthy subjects: a pilot 1H-MRS study. Eur. J. Endocrinol. 179 (2018) 307-317.

[99] S.D. Mistry, G.N. Woods, S. Sigurdsson, S.K. Ewing, T.F. Hue, G. Eiriksdottir, K. Xu, J.F. Hilton, D.M. Kado, V. Gudnason, T.B. Harris, C.J. Rosen, T.F. Lang, X. Li, and A.V. Schwartz, Sex hormones are negatively associated with vertebral bone marrow fat. Bone 108 (2018) 20-24.

[100] S. Muruganandan, R. Govindarajan, and C.J. Sinal, Bone Marrow Adipose Tissue and Skeletal Health. Current osteoporosis reports 16 (2018) 434-442.

[101] V.S. Patel, M. Ete Chan, J. Rubin, and C.T. Rubin, Marrow Adiposity and Hematopoiesis in Aging and Obesity: Exercise as an Intervention. Current osteoporosis reports 16 (2018) 105-115.

[102] B. Yu, L. Huo, Y. Liu, P. Deng, J. Szymanski, J. Li, X. Luo, C. Hong, J. Lin, and C.Y. Wang, PGC-1alpha Controls Skeletal Stem Cell Fate and Bone-Fat Balance in Osteoporosis and Skeletal Aging by Inducing TAZ. Cell Stem Cell 23 (2018) 193-209 e5.

[103] K.M. Beekman, A.G. Veldhuis-Vlug, A. van der Veen, M. den Heijer, M. Maas, G. Kerckhofs, T.N. Parac-Vogt, P.H. Bisschop, and N. Bravenboer, The effect of PPARgamma inhibition on bone marrow adipose tissue and bone in C3H/HeJ mice. Am. J. Physiol. Endocrinol. Metab. 316 (2019) E96-e105.

[104] S. Costa, H. Fairfield, and M.R. Reagan, Inverse correlation between trabecular bone volume and bone marrow adipose tissue in rats treated with osteoanabolic agents. Bone 123 (2019) 211-223.

[105] J. Esche, L. Shi, T. Remer, M.F. Hartmann, S.A. Wudy, and E. Schönau, Glucocorticoids and body fat inversely associate with bone marrow density of the distal radius in healthy youths. J. Clin. Endocrinol. Metab. (2019).

[106] P.K. Fazeli, and A. Klibanski, The paradox of marrow adipose tissue in anorexia nervosa. Bone 118 (2019) 47-52.

[107] H. Hafner, E. Chang, Z. Carlson, A. Zhu, M. Varghese, J. Clemente, S. Abrishami, D.P. Bagchi, O.A. MacDougald, K. Singer, and B. Gregg, Lactational High-Fat Diet Exposure Programs Metabolic Inflammation and Bone Marrow Adiposity in Male Offspring. Nutrients 11 (2019).

[108] C.P. Hawkes, and S. Mostoufi-Moab, Fat-bone interaction within the bone marrow milieu: Impact on hematopoiesis and systemic energy metabolism. Bone 119 (2019) 57-64.

[109] I. Legroux-Gerot, J. Vignau, O. Viltart, P. Hardouin, C. Chauveau, and B. Cortet, Adipokines and bone status in a cohort of anorexic patients. Joint, bone, spine : revue du rhumatisme 86 (2019) 95-101.

[110] Y. Li, Y. Meng, and X. Yu, The Unique Metabolic Characteristics of Bone Marrow Adipose Tissue. Front Endocrinol 10 (2019).

[111] D.E. Maridas, E. Rendina-Ruedy, R.C. Helderman, V.E. DeMambro, D. Brooks, A.R. Guntur, B. Lanske, M.L. Bouxsein, and C.J. Rosen, Progenitor recruitment and adipogenic lipolysis contribute to the anabolic actions of parathyroid hormone on the skeleton. FASEB J. 33 (2019) 2885-2898.

[112] I.C. McCabe, A. Fedorko, M.G. Myers, Jr., G. Leinninger, E. Scheller, and L.R. McCabe, Novel leptin receptor signaling mutants identify location and sex-dependent modulation of bone density, adiposity, and growth. J. Cell. Biochem. 120 (2019) 4398-4408.

[113] L.R. McCabe, R. Irwin, A. Tekalur, C. Evans, J.D. Schepper, N. Parameswaran, and M. Ciancio, Exercise prevents high fat diet-induced bone loss, marrow adiposity and dysbiosis in male mice. Bone 118 (2019) 20-31.

[114] J. Paccou, G. Penel, C. Chauveau, B. Cortet, and P. Hardouin, Marrow adiposity and bone: Review of clinical implications. Bone 118 (2019) 8-15.

[115] J.L. Pierce, D.L. Begun, J.J. Westendorf, and M.E. McGee-Lawrence, Defining osteoblast and adipocyte lineages in the bone marrow. Bone 118 (2019) 2-7.

[116] Z.L. Sebo, E. Rendina-Ruedy, G.P. Ables, D.M. Lindskog, M.S. Rodeheffer, P.K. Fazeli, and M.C. Horowitz, Bone Marrow Adiposity: Basic and Clinical Implications. Endocr. Rev. (2019).

[117] V. Singhal, and M.A. Bredella, Marrow adipose tissue imaging in humans. Bone 118 (2019) 69-76.

[118] R. Zebaze, M. Osima, M. Bui, M. Lukic, X. Wang, A. Ghasem-Zadeh, E.F. Eriksen, A. Vais, C. Shore-Lorenti, P.R. Ebeling, E. Seeman, and A. Bjornerem, Adding Marrow Adiposity and Cortical Porosity to Femoral Neck Areal Bone Mineral Density Improves the Discrimination of Women With Nonvertebral Fractures From Controls. J. Bone Miner. Res. (2019).

[119] L. Zhu, Z. Xu, G. Li, Y. Wang, X. Li, X. Shi, H. Lin, and S. Chang, Marrow adiposity as an indicator for insulin resistance in postmenopausal women with newly diagnosed type 2 diabetes - an investigation by chemical shift-encoded water-fat MRI. Eur. J. Radiol. 113 (2019) 158-164.

[120] G.K. Chan, and G. Duque, Age-related bone loss: old bone, new facts. Gerontology 48 (2002) 62-71.

[121] C.J. Rosen, and M.L. Bouxsein, Mechanisms of disease: is osteoporosis the obesity of bone? Nat Clin Pract Rheumatol 2 (2006) 35-43.

[122] G.F. Maddalozzo, R.T. Turner, C.H. Edwards, K.S. Howe, J.J. Widrick, C.J. Rosen, and U.T. Iwaniec, Alcohol alters whole body composition, inhibits bone formation, and increases bone marrow adiposity in rats. Osteoporos. Int. 20 (2009) 1529-38.

[123] O. Naveiras, V. Nardi, P.L. Wenzel, P.V. Hauschka, F. Fahey, and G.Q. Daley, Bone-marrow adipocytes as negative regulators of the haematopoietic microenvironment. Nature 460 (2009) 259-63.

[124] S. Ambati, Q. Li, S. Rayalam, D.L. Hartzell, M.A. Della-Fera, M.W. Hamrick, and C.A. Baile, Central leptin versus ghrelin: effects on bone marrow adiposity and gene expression. Endocrine 37 (2010) 115-23.

[125] P.J. Menagh, R.T. Turner, D.B. Jump, C.P. Wong, M.B. Lowry, S. Yakar, C.J. Rosen, and U.T. Iwaniec, Growth hormone regulates the balance between bone formation and bone marrow adiposity. J. Bone Miner. Res. 25 (2010) 757-68.

[126] S.M. Bartell, S. Rayalam, S. Ambati, D.R. Gaddam, D.L. Hartzell, M. Hamrick, J.X. She, M.A. Della-Fera, and C.A. Baile, Central (ICV) leptin injection increases bone formation, bone mineral density, muscle mass, serum IGF-1, and the expression of osteogenic genes in leptin-deficient ob/ob mice. J. Bone Miner. Res. 26 (2011) 1710-20.

[127] L.M. Coe, D. Lippner, G.I. Perez, and L.R. McCabe, Caspase-2 deficiency protects mice from diabetes-induced marrow adiposity. J. Cell. Biochem. 112 (2011) 2403-11.

[128] L.F. Liu, W.J. Shen, M. Ueno, S. Patel, and F.B. Kraemer, Characterization of age-related gene expression profiling in bone marrow and epididymal adipocytes. BMC Genomics 12 (2011) 212.

[129] M.M. Rahman, G.V. Halade, P.J. Williams, and G. Fernandes, t10c12-CLA maintains higher bone mineral density during aging by modulating osteoclastogenesis and bone marrow adiposity. J. Cell. Physiol. 226 (2011) 2406-14.

[130] R.T. Turner, C.P. Wong, and U.T. Iwaniec, Effect of reduced c-Kit signaling on bone marrow adiposity. Anat Rec (Hoboken) 294 (2011) 1126-34.

[131] T.A. Wren, S.A. Chung, F.J. Dorey, S. Bluml, G.B. Adams, and V. Gilsanz, Bone marrow fat is inversely related to cortical bone in young and old subjects. J. Clin. Endocrinol. Metab. 96 (2011) 782-6.

[132] K.R. Georgiou, S.K. Hui, and C.J. Xian, Regulatory pathways associated with bone loss and bone marrow adiposity caused by aging, chemotherapy, glucocorticoid therapy and radiotherapy. American Journal of Stem Cells 1 (2012) 205-24.

[133] J. Li, N. Zhang, X. Huang, J. Xu, J.C. Fernandes, K. Dai, and X. Zhang, Dexamethasone shifts bone marrow stromal cells from osteoblasts to adipocytes by C/EBPalpha promoter methylation. Cell death & disease 4 (2013) e832.

[134] H. Sadie-Van Gijsen, F.S. Hough, and W.F. Ferris, Determinants of bone marrow adiposity: the modulation of peroxisome proliferator-activated receptor-gamma2 activity as a central mechanism. Bone 56 (2013) 255-65.

[135] A.L. Hardaway, M.K. Herroon, E. Rajagurubandara, and I. Podgorski, Bone marrow fat: linking adipocyte-induced inflammation with skeletal metastases. Cancer Metastasis Rev. 33 (2014) 527-43.

[136] Z. Xiao, L. Cao, Y. Liang, J. Huang, A.R. Stern, M. Dallas, M. Johnson, and L.D. Quarles, Osteoblast-specific deletion of Pkd2 leads to low-turnover osteopenia and reduced bone marrow adiposity. PLoS One 9 (2014) e114198.

[137] T. Baum, S.P. Yap, M. Dieckmeyer, S. Ruschke, H. Eggers, H. Kooijman, E.J. Rummeny, J.S. Bauer, and D.C. Karampinos, Assessment of whole spine vertebral bone marrow fat using chemical shift-encoding based water-fat MRI. J. Magn. Reson. Imaging 42 (2015) 1018-23.

[138] C.S. Gee, J.T. Nguyen, C.J. Marquez, J. Heunis, A. Lai, C. Wyatt, M. Han, G. Kazakia, A.J. Burghardt, D.C. Karampinos, J. Carballido-Gamio, and R. Krug, Validation of bone marrow fat quantification in the presence of trabecular bone using MRI. J. Magn. Reson. Imaging 42 (2015) 539-44.

[139] O. Ghali, O. Broux, G. Falgayrac, N. Haren, J.P. van Leeuwen, G. Penel, P. Hardouin, and C. Chauveau, Dexamethasone in osteogenic medium strongly induces adipocyte differentiation of mouse bone marrow stromal cells and increases osteoblast differentiation. BMC cell biology 16 (2015) 9.

[140] D.C. Karampinos, S. Ruschke, O. Gordijenko, E. Grande Garcia, H. Kooijman, R. Burgkart, E.J. Rummeny, J.S. Bauer, and T. Baum, Association of MRS-Based Vertebral Bone Marrow Fat Fraction with Bone Strength in a Human In Vitro Model. Journal of osteoporosis 2015 (2015) 152349.

[141] D.C. Karampinos, S. Ruschke, M. Dieckmeyer, H. Eggers, H. Kooijman, E.J. Rummeny, J.S. Bauer, and T. Baum, Modeling of T2* decay in vertebral bone marrow fat quantification. NMR Biomed. 28 (2015) 1535-42.

[142] J. Paccou, P. Hardouin, A. Cotten, G. Penel, and B. Cortet, The Role of Bone Marrow Fat in Skeletal Health: Usefulness and Perspectives for Clinicians. J. Clin. Endocrinol. Metab. 100 (2015) 3613-21.

[143] Y. Qiu, J. Yao, X. Wu, B. Zhou, H. Shao, T. Hua, Z. Xiong, and G. Tang, Longitudinal assessment of oxytocin efficacy on bone and bone marrow fat masses in a rabbit osteoporosis model through 3.0-T magnetic resonance spectroscopy and micro-CT. Osteoporos. Int. 26 (2015) 1081-92.

[144] T. Baum, C. Cordes, M. Dieckmeyer, S. Ruschke, D. Franz, H. Hauner, J.S. Kirschke, and D.C. Karampinos, MR-based assessment of body fat distribution and characteristics. Eur. J. Radiol. (2016).

[145] M.J. Devlin, D.J. Brooks, C. Conlon, M. Vliet, L. Louis, C.J. Rosen, and M.L. Bouxsein, Daily leptin blunts marrow fat but does not impact bone mass in calorie-restricted mice. J. Endocrinol. 229 (2016) 295-306.

[146] G. Di Pietro, S. Capuani, G. Manenti, V. Vinicola, A. Fusco, J. Baldi, M. Scimeca, G. Hagberg, M. Bozzali, G. Simonetti, and U. Tarantino, Bone Marrow Lipid Profiles from Peripheral Skeleton as Potential Biomarkers for Osteoporosis: A 1H-MR Spectroscopy Study. Acad. Radiol. 23 (2016) 273-83.

[147] M.W. Hamrick, M.E. McGee-Lawrence, and D.M. Frechette, Fatty Infiltration of Skeletal Muscle: Mechanisms and Comparisons with Bone Marrow Adiposity. Front Endocrinol (Lausanne) 7 (2016) 69.

[148] J.A. Keune, K.A. Philbrick, A.J. Branscum, U.T. Iwaniec, and R.T. Turner, Spaceflight-induced vertebral bone loss in ovariectomized rats is associated with increased bone marrow adiposity and no change in bone formation. NPJ microgravity 2 (2016) 16016.

[149] T.Y. Kim, and A.L. Schafer, Diabetes and Bone Marrow Adiposity. Current osteoporosis reports (2016).

[150] M.L. Mendonca, S.L. Batista, M.H. Nogueira-Barbosa, C.E. Salmon, and F.J. Paula, Primary Hyperparathyroidism: The Influence of Bone Marrow Adipose Tissue on Bone Loss and of Osteocalcin on Insulin Resistance. Clinics (Sao Paulo, Brazil) 71 (2016) 464-9.

[151] A.M. Pino, M. Miranda, C. Figueroa, J.P. Rodriguez, and C.J. Rosen, Qualitative Aspects of Bone Marrow Adiposity in Osteoporosis. Front Endocrinol (Lausanne) 7 (2016) 139.

[152] J. Plummer, M. Park, F. Perodin, M.C. Horowitz, and J.R. Hens, Methionine-Restricted Diet Increases miRNAs That Can Target RUNX2 Expression and Alters Bone Structure in Young Mice. J. Cell. Biochem. 118 (2017) 31-42.

[153] S. Suresh, J. Caban Alvarez, and C.T. Noguchi, Erythropoietin Eliminates Increased Bone Marrow Adiposity and Alters Bone Features in Obese Mice. Blood 130 (2017) 3778.

[154] J. Wang, S.F. Li, T. Wang, C.H. Sun, L. Wang, M.J. Huang, J. Chen, S.W. Zheng, N. Wang, Y.J. Zhang, and T.Y. Chen, Isopsoralen-mediated suppression of bone marrow adiposity and attenuation of the adipogenic commitment of bone marrow-derived mesenchymal stem cells. Int. J. Mol. Med. 39 (2017) 527-538.

[155] C.N. Withers, D.M. Brown, I. Byiringiro, M.R. Allen, K.W. Condon, J. Satin, and D.A. Andres, Rad GTPase is essential for the regulation of bone density and bone marrow adipose tissue in mice. Bone 103 (2017) 270-280.

[156] M.J. Hernandez, L.M. Dos Reis, I.D. Marques, M.J. Araujo, C.A.M. Truyts, I.B. Oliveira, F.C. Barreto, E. David-Neto, M.R. Custodio, R.M. Moyses, E. Bellorin-Font, and V. Jorgetti, The effect of vitamin D and zoledronic acid in bone marrow adiposity in kidney transplant patients: A post hoc analysis. PLoS One 13 (2018) e0197994.

[157] T. Rharass, and S. Lucas, MECHANISMS IN ENDOCRINOLOGY: Bone Marrow Adiposity and bone, a bad romance? Eur. J. Endocrinol. (2018).

[158] N. Sollmann, M. Dieckmeyer, S. Schlaeger, A. Rohrmeier, J. Syvaeri, M.N. Diefenbach, D. Weidlich, S. Ruschke, E. Klupp, D. Franz, E.J. Rummeny, C. Zimmer, J.S. Kirschke, D.C. Karampinos, and T. Baum, Associations Between Lumbar Vertebral Bone Marrow and Paraspinal Muscle Fat Compositions-An Investigation by Chemical Shift Encoding-Based Water-Fat MRI. Front Endocrinol (Lausanne) 9 (2018) 563.

[159] A.G. Veldhuis-Vlug, and C.J. Rosen, Clinical implications of bone marrow adiposity. J. Intern. Med. 283 (2018) 121-139.

[160] E. Burian, K. Subburaj, M.R.K. Mookiah, A. Rohrmeier, D.M. Hedderich, M. Dieckmeyer, M.N. Diefenbach, S. Ruschke, E.J. Rummeny, C. Zimmer, J.S. Kirschke, D.C. Karampinos, and T. Baum, Texture analysis of vertebral bone marrow using chemical shift encoding–based water-fat MRI: a feasibility study. Osteoporos. Int. (2019).

[161] M. Fujiwara, L. Tian, P.T. Le, V.E. DeMambro, K.A. Becker, C.J. Rosen, and A.R. Guntur, The mitophagy receptor Bcl-2-like protein 13 stimulates adipogenesis by regulating mitochondrial oxidative phosphorylation and apoptosis in mice. J. Biol. Chem. (2019).

[162] B. Lecka-Czernik, and C.J. Rosen, Bone Marrow Adiposity- Special Edition. Bone 118 (2019) 1.

[163] A.M.C. Lee, J.M. Bowen, Y.W. Su, E. Plews, R. Chung, D.M.K. Keefe, and C.J. Xian, Individual or combination treatments with lapatinib and paclitaxel cause potential bone loss and bone marrow adiposity in rats. J. Cell. Biochem. 120 (2019) 4180-4191.

[164] E.V. Morris, and C.M. Edwards, Bone marrow adiposity and multiple myeloma. Bone 118 (2019) 42-46.

[165] Z. Zhang, Z. Huang, B. Ong, C. Sahu, H. Zeng, and H.B. Ruan, Bone marrow adipose tissue-derived stem cell factor mediates metabolic regulation of hematopoiesis. Haematologica (2019).

[166] G.V. Halade, M.M. Rahman, P.J. Williams, and G. Fernandes, Combination of conjugated linoleic acid with fish oil prevents age-associated bone marrow adiposity in C57Bl/6J mice. J Nutr Biochem 22 (2011) 459-69.

[167] R.J. Sulston, and W.P. Cawthorn, Bone marrow adipose tissue as an endocrine organ: close to the bone? Horm Mol Biol Clin Investig 28 (2016) 21-38.

[168] W.P. Cawthorn, and E.L. Scheller, Editorial: Bone Marrow Adipose Tissue: Formation, Function, and Impact on Health and Disease. Front Endocrinol (Lausanne) 8 (2017) 112.

[169] M.S. Shafat, T. Oellerich, S. Mohr, S.D. Robinson, D.R. Edwards, C.R. Marlein, R.E. Piddock, M. Fenech, L. Zaitseva, A. Abdul-Aziz, J. Turner, J.A. Watkins, M. Lawes, K.M. Bowles, and S.A. Rushworth, Leukemic blasts program bone marrow adipocytes to generate a protumoral microenvironment. Blood 129 (2017) 1320-1332.

[170] E.V. Morris, and C.M. Edwards, Adipokines, adiposity, and bone marrow adipocytes: Dangerous accomplices in multiple myeloma. J. Cell. Physiol. 233 (2018) 9159-9166.

[171] K.J. Suchacki, and W.P. Cawthorn, Molecular Interaction of Bone Marrow Adipose Tissue with Energy Metabolism. Curr Mol Biol Rep 4 (2018) 41-49.

[172] M. Tencerova, F. Figeac, N. Ditzel, H. Taipaleenmaki, T.K. Nielsen, and M. Kassem, High-Fat Diet-Induced Obesity Promotes Expansion of Bone Marrow Adipose Tissue and Impairs Skeletal Stem Cell Functions in Mice. J. Bone Miner. Res. 33 (2018) 1154-1165.

[173] C. Attané, D. Estève, K. Chaoui, J. Iacovoni, J. Corre, M. Moutahir, P. Valet, O. Schiltz, N. Reina, and C. Muller, Yellow adipocytes comprise a new adipocyte sub-type present in human bone marrow. bioRxiv (2019) 641886.

[174] V. Cuminetti, and L. Arranz, Bone Marrow Adipocytes: The Enigmatic Components of the Hematopoietic Stem Cell Niche. Journal of Clinical Medicine 8 (2019) 707.

[175] A.S. Gorgey, H.J. Poarch, R.A. Adler, R.E. Khalil, and D.R. Gater, Femoral bone marrow adiposity and cortical bone cross-sectional areas in men with motor complete spinal cord injury. PM & R : the journal of injury, function, and rehabilitation 5 (2013) 939-48.

[176] O. Ghali, N. Al Rassy, P. Hardouin, and C. Chauveau, Increased Bone Marrow Adiposity in a Context of Energy Deficit: The Tip of the Iceberg? Front Endocrinol (Lausanne) 7 (2016) 125.

[177] P. Hardouin, P.J. Marie, and C.J. Rosen, New insights into bone marrow adipocytes: Report from the First European Meeting on Bone Marrow Adiposity (BMA 2015). Bone 93 (2016) 212-215.

[178] B. van der Eerden, and A. van Wijnen, Meeting report of the 2016 bone marrow adiposity meeting. Adipocyte 6 (2017) 304-313.

[179] G.N. Woods, S.K. Ewing, S. Sigurdsson, D.M. Kado, J.H. Ix, T.F. Hue, G. Eiriksdottir, K. Xu, V. Gudnason, T.J. Lang, E. Vittinghoff, T.B. Harris, C.J. Rosen, X. Li, and A.V. Schwartz, Chronic Kidney Disease is Associated with Greater Bone Marrow Adiposity. J. Bone Miner. Res. (2018).

[180] S. Badr, I. Legroux-Gérot, J. Vignau, C. Chauveau, S. Ruschke, D.C. Karampinos, J.-F. Budzik, B. Cortet, and A. Cotten, Comparison of regional bone marrow adiposity

characteristics at the hip of underweight and weight-recovered women with anorexia nervosa using magnetic resonance spectroscopy. Bone (2019).

[181] A. Corsi, B. Palmisano, J. Tratwal, M. Riminucci, and O. Naveiras, Brief Report From the 3rd International Meeting on Bone Marrow Adiposity (BMA 2017). Front Endocrinol (Lausanne) 10 (2019) 336.

[182] X. Coutel, J. Delattre, P. Marchandise, G. Falgayrac, H. Behal, G. Kerckhofs, G. Penel, and C. Olejnik, Mandibular bone is protected against microarchitectural alterations and bone marrow adipose conversion in ovariectomized rats. Bone 127 (2019) 343-352.

[183] J.G. Maciel, I.M. de Araujo, A.L. Carvalho, M.N. Simao, C.M. Bastos, L.E. Troncon, C.E. Salmon, F.J. de Paula, and M.H. Nogueira-Barbosa, Marrow Fat Quality Differences by Sex in Healthy Adults. Journal of clinical densitometry : the official journal of the International Society for Clinical Densitometry 20 (2017) 106-113.

[184] J. Coats, A Manual of Pathology, H. C. Lea's Son and Co., Philadelphia, PA, 1883.

[185] R. Muir, and W.B. Drummond, On the Structure of the Bone-Marrow in Relation to Blood-Formation. Journal of anatomy and physiology 28 (1893) 125-41.

[186] B.F. Steele, The Effects Of Blood Loss And Blood Destruction Upon The Erythroid Cells In The Bone Marrow Of Rabbits. The Journal of Experimental Medicine 57 (1933) 881-896.

[187] C. Huggins, and B.H. Blocksom, Changes in Outlying Bone Marrow Accompanying a Local Increase of Temperature within Physiological Limits. J. Exp. Med. 64 (1936) 253-74.

[188] T.P. Hilditch, and K.S. Murti, The component acids of an ox bone marrow fat. The Biochemical journal 34 (1940) 1299-1300.

[189] L. Berman, A.R. Axelrod, T.N. Horan, S.D. Jacobson, E.A. Sharp, and E.C. Vonderheide, The blood and bone marrow in patients with cirrhosis of the liver. Blood 4 (1949) 511-533.

[190] A. Maniatis, M. Tavassoli, and W.H. Crosby, Factors affecting the conversion of yellow to red marrow. Blood 37 (1971) 581-6.

[191] M. Tavassoli, Marrow adipose cells. Histochemical identification of labile and stable components. Arch. Pathol. Lab. Med. 100 (1976) 16-8.

[192] M. Tavassoli, D.N. Houchin, and P. Jacobs, Fatty acid composition of adipose cells in red and yellow marrow: A possible determinant of haematopoietic potential. Scand. J. Haematol. 18 (1977) 47-53.

[193] S. Trubowitz, and A. Bathija, Cell size and plamitate-1-14c turnover of rabbit marrow fat. Blood 49 (1977) 599-605.

[194] A. Bathija, M. Ohanian, S. Davis, and S. Trubowitz, The marrow fat cell: response to X-ray induced aplasia. Life Sci. 25 (1979) 921-7.

[195] A. Bathija, S. Davis, and S. Trubowitz, Bone marrow adipose tissue: response to acute starvation. Am. J. Hematol. 6 (1979) 191-8.

[196] M. Tavassoli, L.R. Watson, and R. Khademi, Retention of hemopoiesis in tail vertebrae of newborn rats. Cell Tissue Res. 200 (1979) 215-22.

[197] M.A. Maloney, M.L. Flannery, and H.M. Patt, Fat Content of Ectopic Marrow Implants and Cellularity of Resulting Ossicles. Proc. Soc. Exp. Biol. Med. 165 (1980) 309-312.

[198] C.L. Bigelow, and M. Tavassoli, Fatty involution of bone marrow in rabbits. Acta Anat. (Basel). 118 (1984) 60-4.

[199] C.L. Bigelow, and M. Tavassoli, Studies on conversion of yellow marrow to red marrow by using ectopic bone marrow implants. Exp. Hematol. 12 (1984) 581-5.

[200] M. Tavassoli, Hemopoiesis in ectopically implanted bone marrow. Kroc Found. Ser. 18 (1984) 31-54.

[201] M.E. Kricun, Red-yellow marrow conversion: its effect on the location of some solitary bone lesions. Skeletal Radiol. 14 (1985) 10-9.

[202] G.L. Wismer, B.R. Rosen, R. Buxton, D.D. Stark, and T.J. Brady, Chemical shift imaging of bone marrow: preliminary experience. AJR. Am. J. Roentgenol. 145 (1985) 1031-7.

[203] C.S. McKinstry, R.E. Steiner, A.T. Young, L. Jones, D. Swirsky, and V. Aber, Bone marrow in leukemia and aplastic anemia: MR imaging before, during, and after treatment. Radiology 162 (1987) 701-7.

[204] J.M. Gimble, The function of adipocytes in the bone marrow stroma. New Biol. 2 (1990) 304-12.

[205] R.B. Martin, B.D. Chow, and P.A. Lucas, Bone marrow fat content in relation to bone remodeling and serum chemistry in intact and ovariectomized dogs. Calcif. Tissue Int. 46 (1990) 189-94.

[206] S.G. Moore, and K.L. Dawson, Red and yellow marrow in the femur: age-related changes in appearance at MR imaging. Radiology 175 (1990) 219-23.

[207] G.E. Orlandini, L. Ruggiero, M. Gulisano, M. Ruggiero, N. Villari, and F. Casamassima, Magnetic resonance (MR) evaluation of bone marrow in vertebral bodies. Arch. Ital. Anat. Embriol. 96 (1991) 93-100.

[208] K.L. Dawson, S.G. Moore, and J.M. Rowland, Age-related marrow changes in the pelvis: MR and anatomic findings. Radiology 183 (1992) 47-51.

[209] T.M. Simonson, and S.C. Kao, Normal childhood developmental patterns in skull bone marrow by MR imaging. Pediatr. Radiol. 22 (1992) 556-9.

[210] E. De Bisschop, R. Luypaert, O. Louis, and M. Osteaux, Fat fraction of lumbar bone marrow using in vivo proton nuclear magnetic resonance spectroscopy. Bone 14 (1993) 133-6.

[211] S.H. Duda, M. Laniado, F. Schick, M. Strayle, and C.D. Claussen, Normal bone marrow in the sacrum of young adults: differences between the sexes seen on chemical-shift MR imaging. AJR. Am. J. Roentgenol. 164 (1995) 935-40.

[212] A. Taccone, M. Oddone, M. Occhi, A.D. Dell'Acqua, and M.A. Ciccone, MRI "road-map" of normal age-related bone marrow. I. Cranial bone and spine. Pediatr. Radiol. 25 (1995) 588-95.

[213] M. Yamada, T. Matsuzaka, M. Uetani, K. Hayashi, Y. Tsuji, and T. Nakamura, Normal age-related conversion of bone marrow in the mandible: MR imaging findings. AJR. Am. J. Roentgenol. 165 (1995) 1223-8.

[214] B.C. Vande Berg, J. Malghem, F.E. Lecouvet, and B. Maldague, Magnetic resonance imaging of normal bone marrow. Eur. Radiol. 8 (1998) 1327-34.

[215] B.C. Vande Berg, J. Malghem, F.E. Lecouvet, J.P. Devogelaer, B. Maldague, and F.A. Houssiau, Fat conversion of femoral marrow in glucocorticoid-treated patients: a cross-sectional and longitudinal study with magnetic resonance imaging. Arthritis Rheum. 42 (1999) 1405-11.

[216] C. Hollak, M. Maas, E. Akkerman, A. den Heeten, and H. Aerts, Dixon quantitative chemical shift imaging is a sensitive tool for the evaluation of bone marrow responses to individualized doses of enzyme supplementation therapy in type 1 Gaucher disease. Blood Cells. Mol. Dis. 27 (2001) 1005-12.

[217] P. Bianco, and P.G. Robey, Skeletal Stem Cells. in: R. Lanza, J. Gearhart, B. Hogan, D. Melton, R. Pedersen, J. Thomson, and M. West, (Eds.), Handbook of Adult and Fetal Stem Cells, Academic Press, Burlington, 2004, pp. 415-424.

[218] S.A. Kuznetsov, M. Riminucci, N. Ziran, T.W. Tsutsui, A. Corsi, L. Calvi, H.M. Kronenberg, E. Schipani, P.G. Robey, and P. Bianco, The interplay of osteogenesis and hematopoiesis: expression of a constitutively active PTH/PTHrP receptor in osteogenic cells perturbs the establishment of hematopoiesis in bone and of skeletal stem cells in the bone marrow. J. Cell Biol. 167 (2004) 1113-22.

[219] B.C. Vande Berg, R. Gilon, J. Malghem, F. Lecouvet, G. Depresseux, and F.A. Houssiau, Correlation between baseline femoral neck marrow status and the development of femoral head osteonecrosis in corticosteroid-treated patients: a longitudinal study by MR imaging. Eur. J. Radiol. 58 (2006) 444-9.

[220] A. Bosy-Westphal, W. Later, B. Schautz, M. Lagerpusch, K. Goele, M. Heller, C.C. Gluer, and M.J. Muller, Impact of intra- and extra-osseous soft tissue composition on changes in bone mineral density with weight loss and regain. Obesity 19 (2011) 1503-10.

[221] S.R. Tuljapurkar, T.R. McGuire, S.K. Brusnahan, J.D. Jackson, K.L. Garvin, M.A. Kessinger, J.T. Lane, O.K. BJ, and J.G. Sharp, Changes in human bone marrow fat content associated with changes in hematopoietic stem cell numbers and cytokine levels with aging. J. Anat. 219 (2011) 574-81.

[222] G. Trudel, E. Coletta, I. Cameron, D.L. Belavy, M. Lecompte, G. Armbrecht, D. Felsenberg, and H.K. Uhthoff, Resistive exercises, with or without whole body vibration, prevent vertebral marrow fat accumulation during 60 days of head-down tilt bed rest in men. J Appl Physiol (1985) 112 (2012) 1824-31.

[223] P.J. Bolan, L. Arentsen, T. Sueblinvong, Y. Zhang, S. Moeller, J.S. Carter, L.S. Downs, R. Ghebre, D. Yee, J. Froelich, and S. Hui, Water-fat MRI for assessing changes in bone marrow composition due to radiation and chemotherapy in gynecologic cancer patients. J. Magn. Reson. Imaging (2013).

[224] A.L. Newton, L.J. Hanks, M. Davis, and K. Casazza, The relationships among total body fat, bone mineral content and bone marrow adipose tissue in early-pubertal girls. BoneKEy Rep 2 (2013) 1-7.

[225] W. Shen, X. Gong, J. Weiss, and Y. Jin, Comparison among T1-weighted magnetic resonance imaging, modified dixon method, and magnetic resonance spectroscopy in measuring bone marrow fat. J Obes 2013 (2013) 298675.

[226] R.J. Zhu, M.Q. Wu, Z.J. Li, Y. Zhang, and K.Y. Liu, Hematopoietic recovery following chemotherapy is improved by BADGE-induced inhibition of adipogenesis. Int. J. Hematol. 97 (2013) 58-72.

[227] R. Carmona, J. Pritz, M. Bydder, S. Gulaya, H. Zhu, C.W. Williamson, C.S. Welch, F. Vaida, G. Bydder, and L.K. Mell, Fat composition changes in bone marrow during chemotherapy and radiation therapy. Int. J. Radiat. Oncol. Biol. Phys. 90 (2014) 155-63.

[228] P. Hardouin, V. Pansini, and B. Cortet, Bone marrow fat. Joint, bone, spine : revue du rhumatisme 81 (2014) 313-9.

[229] D.C. Karampinos, G. Melkus, T. Baum, J.S. Bauer, E.J. Rummeny, and R. Krug, Bone marrow fat quantification in the presence of trabecular bone: initial comparison between water-fat imaging and single-voxel MRS. Magn. Reson. Med. 71 (2014) 1158-65.

[230] X. Ojanen, R.J. Borra, M. Havu, S.M. Cheng, R. Parkkola, P. Nuutila, M. Alen, and S. Cheng, Comparison of vertebral bone marrow fat assessed by 1H MRS and inphase and out-of-phase MRI among family members. Osteoporos. Int. 25 (2014) 653-62.

[231] P. Bianco, and P.G. Robey, Skeletal stem cells. Development 142 (2015) 1023-7.

[232] M. Dieckmeyer, S. Ruschke, C. Cordes, S.P. Yap, H. Kooijman, H. Hauner, E.J. Rummeny, J.S. Bauer, T. Baum, and D.C. Karampinos, The need for T(2) correction on MRS-based vertebral bone marrow fat quantification: implications for bone marrow fat fraction age dependence. NMR Biomed. 28 (2015) 432-9.

[233] Y. Gao, K. Zong, Z. Gao, M.R. Rubin, J. Chen, S.B. Heymsfield, D. Gallagher, and W. Shen, Magnetic resonance imaging-measured bone marrow adipose tissue area is inversely related to cortical bone area in children and adolescents aged 5-18 years. Journal of clinical densitometry : the official journal of the International Society for Clinical Densitometry 18 (2015) 203-8.

[234] E.L. Scheller, C.R. Doucette, B.S. Learman, W.P. Cawthorn, S. Khandaker, B. Schell, B. Wu, S.Y. Ding, M.A. Bredella, P.K. Fazeli, B. Khoury, K.J. Jepsen, P.F. Pilch, A. Klibanski, C.J. Rosen, and O.A. MacDougald, Region-specific variation in the properties of skeletal adipocytes reveals regulated and constitutive marrow adipose tissues. Nature communications 6 (2015) 7808.

[235] C. Schraml, M. Schmid, S. Gatidis, H. Schmidt, C. la Fougere, K. Nikolaou, and N.F. Schwenzer, Multiparametric analysis of bone marrow in cancer patients using simultaneous PET/MR imaging: Correlation of fat fraction, diffusivity, metabolic activity, and anthropometric data. J. Magn. Reson. Imaging 42 (2015) 1048-56.

[236] Z.S. Templeton, W.R. Lie, W. Wang, Y. Rosenberg-Hasson, R.V. Alluri, J.S. Tamaresis, M.H. Bachmann, K. Lee, W.J. Maloney, C.H. Contag, and B.L. King, Breast Cancer Cell Colonization of the Human Bone Marrow Adipose Tissue Niche. Neoplasia 17 (2015) 849-61.

[237] C. Cordes, T. Baum, M. Dieckmeyer, S. Ruschke, M.N. Diefenbach, H. Hauner, J.S. Kirschke, and D.C. Karampinos, MR-Based Assessment of Bone Marrow Fat in Osteoporosis, Diabetes, and Obesity. Front Endocrinol (Lausanne) 7 (2016) 74.

[238] E.L. Scheller, W.P. Cawthorn, A.A. Burr, M.C. Horowitz, and O.A. MacDougald, Marrow Adipose Tissue: Trimming the Fat. Trends Endocrinol Metab 27 (2016) 392-403.

[239] A.L. Boyd, J.C. Reid, K.R. Salci, L. Aslostovar, Y.D. Benoit, Z. Shapovalova, M. Nakanishi, D.P. Porras, M. Almakadi, C.J.V. Campbell, M.F. Jackson, C.A. Ross, R. Foley, B. Leber, D.S. Allan, M. Sabloff, A. Xenocostas, T.J. Collins, and M. Bhatia, Acute myeloid leukaemia

disrupts endogenous myelo-erythropoiesis by compromising the adipocyte bone marrow niche. Nat. Cell. Biol. (2017).

[240] K. Ecklund, S. Vajapeyam, R.V. Mulkern, H.A. Feldman, J.M. O'Donnell, A.D. DiVasta, and C.M. Gordon, Bone marrow fat content in 70 adolescent girls with anorexia nervosa: Magnetic resonance imaging and magnetic resonance spectroscopy assessment. Pediatr. Radiol. 47 (2017) 952-962.

[241] J.S. Gregory, R.J. Barr, V. Varela, T.S. Ahearn, J.L. Gardiner, F.J. Gilbert, T.W. Redpath, J.D. Hutchison, and R.M. Aspden, MRI and the distribution of bone marrow fat in hip osteoarthritis. J. Magn. Reson. Imaging 45 (2017) 42-50.

[242] B. Lecka-Czernik, L.A. Stechschulte, P.J. Czernik, S.B. Sherman, S. Huang, and A. Krings, Marrow Adipose Tissue: Skeletal Location, Sexual Dimorphism, and Response to Sex Steroid Deficiency. Front Endocrinol (Lausanne) 8 (2017) 188.

[243] D.G. Whitney, H. Singh, F. Miller, M.F. Barbe, J.M. Slade, R.T. Pohlig, and C.M. Modlesky, Cortical bone deficit and fat infiltration of bone marrow and skeletal muscle in ambulatory children with mild spastic cerebral palsy. Bone 94 (2017) 90-97.

[244] B.O. Zhou, H. Yu, R. Yue, Z. Zhao, J.J. Rios, O. Naveiras, and S.J. Morrison, Bone marrow adipocytes promote the regeneration of stem cells and haematopoiesis by secreting SCF. Nat. Cell. Biol. 19 (2017) 891-903.

[245] T. Baum, A. Rohrmeier, J. Syväri, M.N. Diefenbach, D. Franz, M. Dieckmeyer, A. Scharr, H. Hauner, S. Ruschke, J.S. Kirschke, and D.C. Karampinos, Anatomical Variation of Age-Related Changes in Vertebral Bone Marrow Composition Using Chemical Shift Encoding-Based Water–Fat Magnetic Resonance Imaging. Front Endocrinol 9 (2018) 141.

[246] A.R.G. Cortes, O. Cohen, M. Zhao, E.M. Aoki, R.A. Ribeiro, L. Abu Nada, C. Costa, E.S. Arita, F. Tamimi, and J.L. Ackerman, Assessment of alveolar bone marrow fat content using 15 T MRI. Oral surgery, oral medicine, oral pathology and oral radiology 125 (2018) 244-249.

[247] C.S. Craft, N.K. Wee, and E.L. Scheller, Adipocytes and Bone, Primer on the Metabolic Bone Diseases and Disorders of Mineral Metabolism, 2018.

[248] B. Lecka-Czernik, S. Baroi, L.A. Stechschulte, and A.S. Chougule, Marrow Fat-a New Target to Treat Bone Diseases? Current osteoporosis reports 16 (2018) 123-129.

[249] Z. Li, J. Hardij, D.P. Bagchi, E.L. Scheller, and O.A. MacDougald, Development, regulation, metabolism and function of bone marrow adipose tissues. Bone 110 (2018) 134-140.

[250] M. Quittner, T. Rantalainen, N.D. Ridgers, G. Trudel, A. Sheikh, D. Connell, and D.L. Belavy, Intervertebral disc status is associated with vertebral marrow adipose tissue and muscular endurance. Eur. Spine J. 27 (2018) 1704-1711.

[251] T.I. Raglus, B. De Groef, S. Rochfort, G. Rawlin, and C. McCowan, Bone marrow fat analysis as a diagnostic tool to document ante-mortem starvation. The Veterinary Journal ePub ahead of Print (2018).

[252] M. Salas-Ramirez, J. Tran-Gia, C. Kesenheimer, A.M. Weng, A. Kosmala, A. Heidemeier, H. Köstler, and M. Lassmann, Quantification of fat fraction in lumbar vertebrae: correlation with age and implications for bone marrow dosimetry in molecular radiotherapy. Phys. Med. Biol. 63 (2018) 025029.

[253] V. Singhal, L.P. Torre Flores, F.C. Stanford, A.T. Toth, B. Carmine, M. Misra, and M.A. Bredella, Differential associations between appendicular and axial marrow adipose tissue with bone microarchitecture in adolescents and young adults with obesity. Bone 116 (2018) 203-206.

[254] R.T. Turner, S.A. Martin, and U.T. Iwaniec, Metabolic Coupling Between Bone Marrow Adipose Tissue and Hematopoiesis. Current osteoporosis reports 16 (2018) 95-104.

[255] K. Xu, S. Sigurdsson, V. Gudnason, T. Hue, A. Schwartz, and X. Li, Reliable quantification of marrow fat content and unsaturation level using in vivo MR spectroscopy. Magn. Reson. Med. 79 (2018) 1722-1729.

[256] X. Cheng, G.M. Blake, Z. Guo, J. Keenan Brown, L. Wang, K. Li, and L. Xu, Correction of QCT vBMD using MRI measurements of marrow adipose tissue. Bone 120 (2019) 504-511.

[257] K.J. Suchacki, A.A.S. Tavares, D. Mattiucci, E.L. Scheller, G. Papanastasiou, C. Gray, M.C. Sinton, L.E. Ramage, W.A. McDougald, A. Lovdel, R.J. Sulston, B.J. Thomas, B.M. Nicholson, A.J. Drake, C. Alcaide-Corral, D. Said, A. Poloni, S. Cinti, G.J. MacPherson, M.R. Dweck, J.P.M. Andrews, M.C. Williams, R.J. Wallace, E.J.R. van Beek, O.A. MacDougald, N.M. Morton, R.H. Stimson, and W.P. Cawthorn, Bone marrow adipose tissue is a unique adipose subtype with distinct roles in systemic glucose homeostasis. bioRxiv (2019) 673129.

[258] M. Tencerova, M. Frost, F. Figeac, T.K. Nielsen, D. Ali, J.L. Lauterlein, T.L. Andersen, A.K. Haakonsson, A. Rauch, J.S. Madsen, C. Ejersted, K. Hojlund, and M. Kassem, Obesity-Associated Hypermetabolism and Accelerated Senescence of Bone Marrow Stromal Stem Cells Suggest a Potential Mechanism for Bone Fragility. Cell reports 27 (2019) 2050-2062 e6.

[259] K. Ecklund, S. Vajapeyam, H.A. Feldman, C.D. Buzney, R.V. Mulkern, P.K. Kleinman, C.J. Rosen, and C.M. Gordon, Bone marrow changes in adolescent girls with anorexia nervosa. J. Bone Miner. Res. 25 (2010) 298-304.

[260] A.P. Shah, P.W. Patton, D.A. Rajon, and W.E. Bolch, Adipocyte spatial distributions in bone marrow: implications for skeletal dosimetry models. J. Nucl. Med. 44 (2003) 774-83.

[261] M. Tokunaga, E. Wakamatsu, M. Sato, O. Namiki, A. Yokosawa, and M. Motomiya, Lipid composition of adipose tissue from "membranous lipodystrophy". Tohoku J. Exp. Med. 133 (1981) 451-6.

[262] J.K. Zawin, and D. Jaramillo, Conversion of bone marrow in the humerus, sternum, and clavicle: changes with age on MR images. Radiology 188 (1993) 159-64.

[263] B. Chowdhury, L. Sjostrom, M. Alpsten, J. Kostanty, H. Kvist, and R. Lofgren, A multicompartment body composition technique based on computerized tomography. Int. J. Obes. Relat. Metab. Disord. 18 (1994) 219-34.

[264] C. Altehoefer, A. Schmid, M. Buchert, N.A. Ghanem, L. Heinrich, and M. Langer, Characterization of hematopoietic bone marrow in male professional cyclists by magnetic resonance imaging of the lumbar spine. J. Magn. Reson. Imaging 16 (2002) 284-8.

[265] O. Gurevitch, S. Slavin, and A.G. Feldman, Conversion of red bone marrow into yellow - Cause and mechanisms. Med. Hypotheses 69 (2007) 531-6.

[266] A. Poloni, G. Maurizi, F. Serrani, S. Mancini, M.C. Zingaretti, A. Frontini, S. Cinti, A. Olivieri, and P. Leoni, Molecular and functional characterization of human bone marrow adipocytes. Exp. Hematol. 41 (2013) 558-566 e2.

[267] V. Pansini, A. Monnet, J. Salleron, P. Hardouin, B. Cortet, and A. Cotten, 3 Tesla (1) H MR spectroscopy of hip bone marrow in a healthy population, assessment of normal fat content values and influence of age and sex. J. Magn. Reson. Imaging 39 (2014) 369-76.

[268] P. Hardouin, T. Rharass, and S. Lucas, Bone Marrow Adipose Tissue: To Be or Not To Be a Typical Adipose Tissue? Front Endocrinol (Lausanne) 7 (2016) 85.

[269] T.H. Ambrosi, A. Scialdone, A. Graja, S. Gohlke, A.M. Jank, C. Bocian, L. Woelk, H. Fan, D.W. Logan, A. Schurmann, L.R. Saraiva, and T.J. Schulz, Adipocyte Accumulation in the Bone Marrow during Obesity and Aging Impairs Stem Cell-Based Hematopoietic and Bone Regeneration. Cell Stem Cell 20 (2017) 771-784 e6.

[270] A.S. Gorgey, P.D. Moore, R.C. Wade, R.S. Gill, T. Lavis, and R.A. Adler, Disruption in bone marrow fat may attenuate testosterone action on muscle size after spinal cord injury: a case report. European journal of physical and rehabilitation medicine 53 (2017) 625-629.

[271] S. Ruschke, A. Pokorney, T. Baum, H. Eggers, J.H. Miller, H.H. Hu, and D.C. Karampinos, Measurement of vertebral bone marrow proton density fat fraction in children using quantitative water-fat MRI. MAGMA 30 (2017) 449-460.

[272] J. Bukowska, T. Frazier, S. Smith, T. Brown, R. Bender, M. McCarthy, X. Wu, B.A. Bunnell, and J.M. Gimble, Bone Marrow Adipocyte Developmental Origin and Biology. Current osteoporosis reports (2018).

[273] H. Wang, Y. Leng, and Y. Gong, Bone Marrow Fat and Hematopoiesis. Front Endocrinol (Lausanne) 9 (2018) 694.

[274] M. Zhu, G. Hao, J. Xing, S. Hu, D. Geng, W. Zhang, Q. Wang, C. Hu, and X. Wang, Bone marrow adipose amount influences vertebral bone strength. Experimental and therapeutic medicine 17 (2019) 689-694.

[275] E. Zakaria, and E. Shafrir, Yellow bone marrow as adipose tissue. Proc. Soc. Exp. Biol. Med. 124 (1967) 1265-8.

[276] J. Lundbom, A. Bierwagen, K. Bodis, M. Apostolopoulou, J. Szendroedi, K. Mussig, J.H. Hwang, and M. Roden, (1)H-MRS of femoral red and yellow bone marrow fat composition and water content in healthy young men and women at 3 T. MAGMA (2019).

[277] A. Argiris, T. Maris, G. Papavasiliou, A. Gouliamos, and C. Papavasiliou, Radiotherapy effects on vertebral bone marrow: easily recognizable changes in T2 relaxation times. Magn. Reson. Imaging 14 (1996) 633-8.

[278] Z. Vrselja, and G. Curic, Vertebral marrow adipose tissue adipokines as a possible cause of intervertebral disc inflammation. Joint, bone, spine : revue du rhumatisme 85 (2018) 143-146.

[279] V. Huovinen, V. Saunavaara, R. Kiviranta, M. Tarkia, H. Honka, C. Stark, J. Laine, K. Linderborg, P. Tuomikoski, R.M. Badeau, J. Knuuti, P. Nuutila, and R. Parkkola, Vertebral bone marrow glucose uptake is inversely associated with bone marrow fat in diabetic and healthy pigs: [(18)F]FDG-PET and MRI study. Bone 61 (2014) 33-8.

[280] C.S. Craft, and E.L. Scheller, Evolution of the Marrow Adipose Tissue Microenvironment. Calcif. Tissue Int. 100 (2017) 461-475.

[281] D.L. Belavy, M.J. Quittner, N.D. Ridgers, A. Shiekh, T. Rantalainen, and G. Trudel, Specific Modulation of Vertebral Marrow Adipose Tissue by Physical Activity. J. Bone Miner. Res. 33 (2018) 651-657.

[282] M. Tavassoli, A. Maniatis, and W.H. Crosby, Induction of sustained hemopoiesis in fatty marrow. Blood 43 (1974) 33-8.

[283] A. Piney, The anatomy of the bone marrow: With special reference to the distribution of the red marrow. Br. Med. J. 1922 (1922) 792-795.

[284] R.P. Custer, and F.E. Ahlfeldt, Studies on the structure and function of bone marrow: II. Variations in cellularity in various bones with advancing years of life and their relative response to stimuli. The Journal of Laboratory and Clinical Medicine 17 (1932) 960-962.

[285] C.P. Rhoads, and W.B. Castle, The Pathology of the Bone Marrow in Sprue Anemia. The American journal of pathology 9 (1933) 813-826.5.

[286] D.H. Andersen, Benzol Poisoning with Hyperplasia of the Bone Marrow. The American journal of pathology 10 (1934) 101-112.1.

[287] R.P. Custer, Studies on the structure and function of bone marrow: IV. Bone Marrow in Agranulocytosis. The American Journal of the Medical Sciences 189 (1935) 507-515.

[288] P.E. Rekers, and M. Coulter, A hematological and histological study of the bone marrow and peripheral blood of the adult dog. Am. J. Med. Sci. 216 (1948) 643-55.

[289] J.L. Emery, and G.F. Follett, Regression of Bone-Marrow Haemopoiesis from the Terminal Digits in the Foetus and Infant. Br. J. Haematol. 10 (1964) 485-489.

[290] M. Tavassoli, and W.H. Crosby, Bone marrow histogenesis: a comparison of fatty and red marrow. Science 169 (1970) 291-3.

[291] P. Meunier, J. Aaron, C. Edouard, and G. Vignon, Osteoporosis and the replacement of cell populations of the marrow by adipose tissue. A quantitative study of 84 iliac bone biopsies. Clin Orthop Relat Res 80 (1971) 147-54.

[292] M. Tavassoli, Differential response of bone marrow and extramedullary adipose cells to starvation. Experientia 30 (1974) 424-5.

[293] S.C. Miller, B.M. Bowman, J.M. Smith, and W.S. Jee, Characterization of endosteal bone-lining cells from fatty marrow bone sites in adult beagles. Anat. Rec. 198 (1980) 163-73.

[294] W.G. Totty, W.A. Murphy, W.I. Ganz, B. Kumar, W.J. Daum, and B.A. Siegel, Magnetic resonance imaging of the normal and ischemic femoral head. AJR. Am. J. Roentgenol. 143 (1984) 1273-80.

[295] H. Yoshida, S. Asai, N. Yashiro, and M. Iio, MRI of bone marrow. Radiat. Med. 3 (1985) 47-55.

[296] K. Kagawa, K. Hayashi, and M. Awai, Participation of bone marrow stromal cells in hemopoietic recovery of rats irradiated and then parabiosed with a non-irradiated litter mate. I. Light microscopic observations. Acta Pathol. Jpn. 36 (1986) 999-1010.

[297] K. Kagawa, K. Hayashi, and M. Awai, Participation of bone marrow stromal cells in hemopoietic recovery of rats irradiated and then parabiosed with a non-irradiated litter mate.

II. Scanning and transmission electron microscopic observations. Acta Pathol. Jpn. 36 (1986) 1011-26.

[298] A. Islam, Do bone marrow fat cells or their precursors have a pathogenic role in idiopathic aplastic anaemia? Med. Hypotheses 25 (1988) 209-17.

[299] R.M. Steiner, D.G. Mitchell, V.M. Rao, S. Murphy, M.D. Rifkin, D.L. Burk, Jr., S.K. Ballas, and S. Vinitski, Magnetic resonance imaging of bone marrow: diagnostic value in diffuse hematologic disorders. Magn. Reson. Q. 6 (1990) 17-34.

[300] S.K. Stevens, S.G. Moore, and I.D. Kaplan, Early and late bone-marrow changes after irradiation: MR evaluation. AJR. Am. J. Roentgenol. 154 (1990) 745-50.

[301] A. LeBlanc, C. Lin, H. Evans, L. Shackelford, C. Martin, and T. Hedrick, T2 vertebral bone marrow changes after space flight. Magn. Reson. Med. 41 (1999) 495-8.

[302] J.F. Griffith, D.K. Yeung, A.T. Ahuja, C.W. Choy, W.Y. Mei, S.S. Lam, T.P. Lam, Z.Y. Chen, and P.C. Leung, A study of bone marrow and subcutaneous fatty acid composition in subjects of varying bone mineral density. Bone 44 (2009) 1092-6.

[303] L. Xiao, T. Sobue, A. Esliger, M.S. Kronenberg, J.D. Coffin, T. Doetschman, and M.M. Hurley, Disruption of the Fgf2 gene activates the adipogenic and suppresses the osteogenic program in mesenchymal marrow stromal stem cells. Bone 47 (2010) 360-70.

[304] W. Shen, J. Chen, M. Gantz, M. Punyanitya, S.B. Heymsfield, D. Gallagher, J. Albu, E. Engelson, D. Kotler, X. Pi-Sunyer, and S. Shapses, Ethnic and sex differences in bone marrow adipose tissue and bone mineral density relationship. Osteoporos. Int. 23 (2012) 2293-301.

[305] W. Shen, R. Scherzer, M. Gantz, J. Chen, M. Punyanitya, C.E. Lewis, and C. Grunfeld, Relationship between MRI-measured bone marrow adipose tissue and hip and spine bone mineral density in African-American and Caucasian participants: the CARDIA study. J. Clin. Endocrinol. Metab. 97 (2012) 1337-46.

[306] P.K. Fazeli, M.C. Horowitz, O.A. Macdougald, E.L. Scheller, M.S. Rodeheffer, C.J. Rosen, and A. Klibanski, Marrow fat and bone--new perspectives. J. Clin. Endocrinol. Metab. 98 (2013) 935-45.

[307] S. Takeshita, T. Fumoto, Y. Naoe, and K. Ikeda, Age-related marrow adipogenesis is linked to increased expression of RANKL. J. Biol. Chem. 289 (2014) 16699-710.

[308] G. Li, Z. Xu, Y. Chen, S. Chang, H. Calimente, J. Hu, and D. Wu, Longitudinal assessment of marrow fat content using three-point Dixon technique in osteoporotic rabbits. Menopause 23 (2016) 1339-1344.

[309] Y. Sheu, F. Amati, A.V. Schwartz, M.E. Danielson, X. Li, R. Boudreau, J.A. Cauley, and G. Osteoporotic Fractures in Men Research, Vertebral bone marrow fat, bone mineral density and diabetes: The Osteoporotic Fractures in Men (MrOS) study. Bone 97 (2017) 299-305.

[310] H.J. Yoo, S.H. Hong, D.H. Kim, J.Y. Choi, H.D. Chae, B.M. Jeong, J.M. Ahn, and H.S. Kang, Measurement of fat content in vertebral marrow using a modified dixon sequence to differentiate benign from malignant processes. J. Magn. Reson. Imaging 45 (2017) 1534-1544.

[311] A. Razek, A. Abdalla, T. Barakat, H. El-Taher, and K. Ali, Multi-parametric MR imaging using apparent diffusion coefficient and fat fraction in quantification of bone marrow in pediatrics with Gaucher disease. Clin. Imaging 51 (2018) 318-322.

[312] P.A. Simkin, Marrow fat may distribute the energy of impact loading throughout subchondral bone. Rheumatology (Oxford). 57 (2018) 414-418.

[313] K.E. Jensen, M. Jensen, P. Grundtvig, C. Thomsen, H. Karle, and O. Henriksen, Localized in vivo proton spectroscopy of the bone marrow in patients with leukemia. Magn. Reson. Imaging 8 (1990) 779-89.

[314] Z. Belaid, F. Hubint, C. Humblet, J. Boniver, B. Nusgens, and M.P. Defresne, Differential expression of vascular endothelial growth factor and its receptors in hematopoietic and fatty bone marrow: evidence that neuropilin-1 is produced by fat cells. Haematologica 90 (2005) 400-1.

[315] K. Uchihashi, S. Aoki, M. Shigematsu, N. Kamochi, E. Sonoda, H. Soejima, K. Fukudome, H. Sugihara, T. Hotokebuchi, and S. Toda, Organotypic culture of human bone marrow adipose tissue. Pathol. Int. 60 (2010) 259-67.

[316] S. Muruganandan, and C.J. Sinal, The impact of bone marrow adipocytes on osteoblast and osteoclast differentiation. IUBMB life (2014).

[317] Z. Belaid-Choucair, Y. Lepelletier, G. Poncin, A. Thiry, C. Humblet, M. Maachi, A. Beaulieu, E. Schneider, A. Briquet, P. Mineur, C. Lambert, D. Mendes-Da-Cruz, M.L. Ahui, V. Asnafi, M. Dy, J. Boniver, B.V. Nusgens, O. Hermine, and M.P. Defresne, Human bone marrow adipocytes block granulopoiesis through neuropilin-1-induced granulocyte colony-stimulating factor inhibition. Stem Cells 26 (2008) 1556-64.

[318] W.P. Cawthorn, E.L. Scheller, S.D. Parlee, H.A. Pham, B.S. Learman, C.M. Redshaw, R.J. Sulston, A.A. Burr, A.K. Das, B.R. Simon, H. Mori, A.J. Bree, B. Schell, V. Krishnan, and O.A. MacDougald, Expansion of Bone Marrow Adipose Tissue During Caloric Restriction Is Associated With Increased Circulating Glucocorticoids and Not With Hypoleptinemia. Endocrinology 157 (2016) 508-21.

[319] J.D. Evans, R.M. Bird, M. Seip, and L. Becker, Metabolism of rabbit bone marrow in vitro in ringer-bicarbonate medium containing no added glucose. J. Biol. Chem. 181 (1949) 357-365.

[320] P. Braidotti, and L. Stagni, A critical damping approach for assessing the role of marrow fat on the mechanical strength of trabecular bone. Med. Hypotheses 69 (2007) 43-6.

[321] B. Lecka-Czernik, Marrow fat metabolism is linked to the systemic energy metabolism. Bone (2011).

[322] C. Falank, H. Fairfield, and M.R. Reagan, Signaling Interplay between Bone Marrow Adipose Tissue and Multiple Myeloma cells. Front Endocrinol (Lausanne) 7 (2016) 67.

[323] K.J. Suchacki, W.P. Cawthorn, and C.J. Rosen, Bone marrow adipose tissue: formation, function and regulation. Current opinion in pharmacology 28 (2016) 50-56.

[324] C.S. Craft, Z. Li, O.A. MacDougald, and E.L. Scheller, Molecular differences between subtypes of bone marrow adipocytes. Curr Mol Biol Rep 4 (2018) 16-23.

[325] Q. Li, Y. Wu, and N. Kang, Marrow Adipose Tissue: Its Origin, Function, and Regulation in Bone Remodeling and Regeneration. Stem cells international 2018 (2018) 7098456.

[326] D. Mattiucci, G. Maurizi, V. Izzi, L. Cenci, M. Ciarlantini, S. Mancini, E. Mensa, R. Pascarella, M. Vivarelli, A. Olivieri, P. Leoni, and A. Poloni, Bone marrow adipocytes support hematopoietic stem cell survival. J. Cell. Physiol. 233 (2018) 1500-1511.

[327] E.L. Scheller, S. Khandaker, B.S. Learman, W.P. Cawthorn, L.M. Anderson, H.A. Pham, H. Robles, Z. Wang, Z. Li, S.D. Parlee, B.R. Simon, H. Mori, A.J. Bree, C.S. Craft, and O.A. MacDougald, Bone marrow adipocytes resist lipolysis and remodeling in response to beta-adrenergic stimulation. Bone 118 (2018) 32-41.

[328] D.K. Miller, and C.P. Rhoads, The Effect Of Hemoglobin Injections On Erythropoiesis And Erythrocyte Size In Rabbits Rendered Anemic By Bleeding. The Journal of Experimental Medicine 59 (1934) 333-346.

[329] J.E. Allen, D.L. Henshaw, P.A. Keitch, A.P. Fews, and J.P. Eatough, Fat cells in red bone marrow of human rib: their size and spatial distribution with respect to the radon-derived dose to the haemopoietic tissue. Int. J. Radiat. Biol. 68 (1995) 669-78.

[330] H. Kugel, C. Jung, O. Schulte, and W. Heindel, Age- and sex-specific differences in the 1H-spectrum of vertebral bone marrow. J. Magn. Reson. Imaging 13 (2001) 263-8.

[331] J.D. Diedrich, M.K. Herroon, E. Rajagurubandara, and I. Podgorski, The Lipid Side of Bone Marrow Adipocytes: How Tumor Cells Adapt and Survive in Bone. Current osteoporosis reports (2018).

[332] J. Bao, X. Cui, Y. Huang, J. Zhong, and Z. Chen, Resolution enhancement in MR spectroscopy of red bone marrow fat via intermolecular double-quantum coherences. Phys. Med. Biol. 60 (2015) 6391-406.

[333] F. Oberling, J.P. Cazenave, and R. Waitz, Ultrastructure of adipose tissue in the normal hematopoietic marrow of the rabbit. Pathol. Biol. (Paris). 20 (1972) 337-47.

[334] J. Justesen, K. Stenderup, E.N. Ebbesen, L. Mosekilde, T. Steiniche, and M. Kassem, Adipocyte tissue volume in bone marrow is increased with aging and in patients with osteoporosis. Biogerontology 2 (2001) 165-71.

[335] J.F. Griffith, D.K. Yeung, H.T. Ma, J.C. Leung, T.C. Kwok, and P.C. Leung, Bone marrow fat content in the elderly: a reversal of sex difference seen in younger subjects. J. Magn. Reson. Imaging 36 (2012) 225-30.

[336] S.K. Curtis, R.R. Cowden, and J.W. Nagel, Ultrastructure of the bone marrow of the salamander Plethodon glutinosus (Caudata: Plethodontidae). J. Morphol. 159 (1979) 151-183.

[337] M. Tavassoli, Ultrastructural development of bone marrow adipose cell. Acta Anat. (Basel). 94 (1976) 65-77.

[338] M.A. Tran, L. Dang Tran, M. Lafontan, and P. Montastruc, Adrenergic neurohumoral influences on FFA release from bone marrow adipose tissue. J. Pharmacol. 16 (1985) 171-9.

[339] J. Hirata, T. Umemura, S. Kaneko, J. Nishimura, S. Motomura, and H. Ibayashi, Difference of bone marrow adipocyte colony-forming capacity between aplastic anemia and iron deficiency anemia. Leuk. Res. 12 (1988) 179-83.

[340] J. Hirata, H. Takahira, S. Kaneko, J. Nishimura, and H. Nawata, Bone Marrow Stromal Cells in Myeloproliferative Disorders. Acta Haematol. 82 (1989) 35-39.

[341] C. Rozman, E. Feliu, M. Rozman, J.C. Reverter, C. Climent, and L. Berga, [Acquired aplastic anemia: a stereological analysis of bone marrow fatty tissue and its clinical correlations]. Med. Clin. (Barc). 101 (1993) 441-5.

[342] M.E. Nuttall, A.J. Patton, D.L. Olivera, D.P. Nadeau, and M. Gowen, Human trabecular bone cells are able to express both osteoblastic and adipocytic phenotype: implications for osteopenic disorders. J. Bone Miner. Res. 13 (1998) 371-82.

[343] A. Gaja, Z. Chury, L. Pecen, H. Fra kova, E. Jandakova, and N. Hejlova, Bone marrow and peripheral blood leptin levels in lymphoproliferative diseases--relation to the bone marrow fat and infiltration. Neoplasma 47 (2000) 307-12.

[344] T. Kurabayashi, M. Tomita, H. Matsushita, A. Honda, K. Takakuwa, and K. Tanaka, Effects of a beta 3 adrenergic receptor agonist on bone and bone marrow adipocytes in the tibia and lumbar spine of the ovariectomized rat. Calcif. Tissue Int. 68 (2001) 248-54.

[345] F.H. Wezeman, and Z. Gong, Bone marrow triglyceride accumulation and hormonal changes during long-term alcohol intake in male and female rats. Alcohol. Clin. Exp. Res. 25 (2001) 1515-22.

[346] E. Abella, E. Feliu, I. Granada, F. Milla, A. Oriol, J.M. Ribera, L. Sanchez-Planell, L.I. Berga, J.C. Reverter, and C. Rozman, Bone marrow changes in anorexia nervosa are correlated with the amount of weight loss and not with other clinical findings. Am. J. Clin. Pathol. 118 (2002) 582-8.

[347] J. Justesen, L. Mosekilde, M. Holmes, K. Stenderup, J. Gasser, J.J. Mullins, J.R. Seckl, and M. Kassem, Mice deficient in 11beta-hydroxysteroid dehydrogenase type 1 lack bone marrow adipocytes, but maintain normal bone formation. Endocrinology 145 (2004) 1916-25.

[348] I. Sekiya, B.L. Larson, J.T. Vuoristo, J.G. Cui, and D.J. Prockop, Adipogenic differentiation of human adult stem cells from bone marrow stroma (MSCs). J. Bone Miner. Res. 19 (2004) 256-64.

[349] M.W. Hamrick, M.A. Della-Fera, Y.H. Choi, C. Pennington, D. Hartzell, and C.A. Baile, Leptin treatment induces loss of bone marrow adipocytes and increases bone formation in leptin-deficient ob/ob mice. J. Bone Miner. Res. 20 (2005) 994-1001.

[350] J.M. Kindblom, E.F. Gevers, S.M. Skrtic, M.K. Lindberg, S. Gothe, J. Tornell, B. Vennstrom, and C. Ohlsson, Increased adipogenesis in bone marrow but decreased bone mineral density in mice devoid of thyroid hormone receptors. Bone 36 (2005) 607-16.

[351] N. Tamura, T. Kurabayashi, H. Nagata, H. Matsushita, T. Yahata, and K. Tanaka, Effects of testosterone on cancellous bone, marrow adipocytes, and ovarian phenotype in a young female rat model of polycystic ovary syndrome. Fertil. Steril. 84 Suppl 2 (2005) 1277-84.

[352] M.W. Hamrick, M.A. Della Fera, Y.H. Choi, D. Hartzell, C. Pennington, and C.A. Baile, Injections of leptin into rat ventromedial hypothalamus increase adipocyte apoptosis in peripheral fat and in bone marrow. Cell Tissue Res. 327 (2007) 133-41.

[353] F.A. Syed, M.J. Oursler, T.E. Hefferanm, J.M. Peterson, B.L. Riggs, and S. Khosla, Effects of estrogen therapy on bone marrow adipocytes in postmenopausal osteoporotic women. Osteoporos. Int. 19 (2008) 1323-30.

[354] M.A. Bredella, P.K. Fazeli, K.K. Miller, M. Misra, M. Torriani, B.J. Thomas, R.H. Ghomi, C.J. Rosen, and A. Klibanski, Increased bone marrow fat in anorexia nervosa. J. Clin. Endocrinol. Metab. 94 (2009) 2129-36.

[355] A. Elbaz, D. Rivas, and G. Duque, Effect of estrogens on bone marrow adipogenesis and Sirt1 in aging C57BL/6J mice. Biogerontology 10 (2009) 747-55.

[356] A. Hozumi, M. Osaki, H. Goto, K. Sakamoto, S. Inokuchi, and H. Shindo, Bone marrow adipocytes support dexamethasone-induced osteoclast differentiation. Biochem. Biophys. Res. Commun. 382 (2009) 780-4.

[357] A. Clabaut, S. Delplace, C. Chauveau, P. Hardouin, and O. Broux, Human osteoblasts derived from mesenchymal stem cells express adipogenic markers upon coculture with bone marrow adipocytes. Differentiation 80 (2010) 40-5.

[358] A. Hozumi, M. Osaki, K. Sakamoto, H. Goto, T. Fukushima, H. Baba, and H. Shindo, Dexamethasone-induced plasminogen activator inhibitor-1 expression in human primary bone marrow adipocytes. Biomedical research 31 (2010) 281-6.

[359] L.F. Liu, W.J. Shen, Z.H. Zhang, L.J. Wang, and F.B. Kraemer, Adipocytes decrease Runx2 expression in osteoblastic cells: roles of PPARgamma and adiponectin. J. Cell. Physiol. 225 (2010) 837-45.

[360] D.F. Razidlo, T.J. Whitney, M.E. Casper, M.E. McGee-Lawrence, B.A. Stensgard, X. Li, F.J. Secreto, S.K. Knutson, S.W. Hiebert, and J.J. Westendorf, Histone deacetylase 3 depletion in osteo/chondroprogenitor cells decreases bone density and increases marrow fat. PLoS One 5 (2010) e11492.

[361] H. Goto, A. Hozumi, M. Osaki, T. Fukushima, K. Sakamoto, A. Yonekura, M. Tomita, K. Furukawa, H. Shindo, and H. Baba, Primary human bone marrow adipocytes support TNF-α-induced osteoclast differentiation and function through RANKL expression. Cytokine 56 (2011) 662-668.

[362] H. Goto, M. Osaki, T. Fukushima, K. Sakamoto, A. Hozumi, H. Baba, and H. Shindo, Human bone marrow adipocytes support dexamethasone-induced osteoclast differentiation and function through RANKL expression. Biomedical research 32 (2011) 37-44.

[363] K. Sakamoto, M. Osaki, A. Hozumi, H. Goto, T. Fukushima, H. Baba, and H. Shindo, Simvastatin suppresses dexamethasone-induced secretion of plasminogen activator inhibitor-1 in human bone marrow adipocytes. BMC musculoskeletal disorders 12 (2011) 82.

[364] J. Chen, and F. Long, beta-catenin promotes bone formation and suppresses bone resorption in postnatal growing mice. J. Bone Miner. Res. 28 (2013) 1160-9.

[365] J. Jin, L. Wang, X.K. Wang, P.L. Lai, M.J. Huang, D.D. Jin, Z.M. Zhong, J.T. Chen, and X.C. Bai, Risedronate inhibits bone marrow mesenchymal stem cell adipogenesis and switches RANKL/OPG ratio to impair osteoclast differentiation. J. Surg. Res. 180 (2013) e21-9.

[366] W. Shen, G. Velasquez, J. Chen, Y. Jin, S.B. Heymsfield, D. Gallagher, and F.X. Pi-Sunyer, Comparison of the Relationship Between Bone Marrow Adipose Tissue and Volumetric Bone Mineral Density in Children and Adults. Journal of Clinical Densitometry (2014).

[367] V. Huovinen, H. Viljakainen, A. Hakkarainen, T. Saukkonen, S. Toiviainen-Salo, N. Lundbom, J. Lundbom, and O. Makitie, Bone marrow fat unsaturation in young adults is not affected by present or childhood obesity, but increases with age: A pilot study. Metabolism. 64 (2015) 1574-81.

[368] B.M. Khoury, E.M. Bigelow, L.M. Smith, S.H. Schlecht, E.L. Scheller, N. Andarawis-Puri, and K.J. Jepsen, The use of nano-computed tomography to enhance musculoskeletal research. Connect. Tissue Res. 56 (2015) 106-19.

[369] J.D. Diedrich, E. Rajagurubandara, M.K. Herroon, G. Mahapatra, M. Huttemann, and I. Podgorski, Bone marrow adipocytes promote the Warburg phenotype in metastatic prostate tumors via HIF-1alpha activation. Oncotarget 7 (2016) 64854-64877.

[370] R.J. Sulston, B.S. Learman, B. Zhang, E.L. Scheller, S.D. Parlee, B.R. Simon, H. Mori, A.J. Bree, R.J. Wallace, V. Krishnan, O.A. MacDougald, and W.P. Cawthorn, Increased Circulating Adiponectin in Response to Thiazolidinediones: Investigating the Role of Bone Marrow Adipose Tissue. Front Endocrinol 7 (2016) 128.

[371] G. van Niekerk, T. Davis, and A.M. Engelbrecht, Bone marrow fat: What is it good for? Semin. Arthritis Rheum. 45 (2016) e14.

[372] Y. Tabe, S. Yamamoto, K. Saitoh, K. Sekihara, N. Monma, K. Ikeo, K. Mogushi, M. Shikami, V. Ruvolo, J. Ishizawa, N. Hail, Jr., S. Kazuno, M. Igarashi, H. Matsushita, Y. Yamanaka, H. Arai, I. Nagaoka, T. Miida, Y. Hayashizaki, M. Konopleva, and M. Andreeff, Bone Marrow Adipocytes Facilitate Fatty Acid Oxidation Activating AMPK and a Transcriptional Network Supporting Survival of Acute Monocytic Leukemia Cells. Cancer Res. 77 (2017) 1453-1464.

[373] N.Y. Yu, T. Wolfson, M.S. Middleton, G. Hamilton, A. Gamst, J.E. Angeles, J.B. Schwimmer, and C.B. Sirlin, Bone marrow fat content is correlated with hepatic fat content in paediatric non-alcoholic fatty liver disease. Clin. Radiol. 72 (2017) 425.e9-425.e14.

[374] W. Lu, W. Weng, Q. Zhu, Y. Zhai, Y. Wan, H. Liu, S. Yang, Y. Yu, Y. Wei, and J. Shi, Small bone marrow adipocytes predict poor prognosis in acute myeloid leukemia. Haematologica 103 (2018) e21-e24.

[375] K. Wang, Y. Zha, H. Lei, and X. Xu, MRI Study on the Changes of Bone Marrow Microvascular Permeability and Fat Content after Total-Body X-Ray Irradiation. Radiat. Res. 189 (2018) 205-212.

[376] D.G. Whitney, M.D. Peterson, M.J. Devlin, M.S. Caird, E.A. Hurvitz, and C.M. Modlesky, Bone marrow fat physiology in relation to skeletal metabolism and cardiometabolic disease risk in children with cerebral palsy. Am. J. Phys. Med. Rehabil. (2018).

[377] K.M. Beekman, A.G. Veldhuis-Vlug, M. den Heijer, M. Maas, A.M. Oleksik, M.W. Tanck, S.M. Ott, R.J. van 't Hof, P. Lips, P.H. Bisschop, and N. Bravenboer, The effect of raloxifene on bone marrow adipose tissue and bone turnover in postmenopausal women with osteoporosis. Bone 118 (2019) 62-68.

[378] S.E. Delikat, D.W. Galvani, and M. Zuzel, The metabolic effects of interleukin 1 beta on human bone marrow adipocytes. Cytokine 7 (1995) 338-43.

[379] P. Laharrague, D. Larrouy, A.M. Fontanilles, N. Truel, A. Campfield, R. Tenenbaum, J. Galitzky, J.X. Corberand, L. Penicaud, and L. Casteilla, High expression of leptin by human bone marrow adipocytes in primary culture. FASEB J. 12 (1998) 747-52.

[380] P. Laharrague, N. Truel, A.M. Fontanilles, J.X. Corberand, L. Penicaud, and L. Casteilla, Regulation by cytokines of leptin expression in human bone marrow adipocytes. Horm. Metab. Res. 32 (2000) 381-5.

[381] P. Laharrague, A.M. Fontanilles, J. Tkaczuk, J.X. Corberand, L. Penicaud, and L. Casteilla, Inflammatory/haematopoietic cytokine production by human bone marrow adipocytes. Eur. Cytokine Netw. 11 (2000) 634-9.

[382] E.F. Gevers, N. Loveridge, and I.C. Robinson, Bone marrow adipocytes: a neglected target tissue for growth hormone. Endocrinology 143 (2002) 4065-73.

[383] J. Corre, V. Planat-Benard, J.X. Corberand, L. Penicaud, L. Casteilla, and P. Laharrague, Human bone marrow adipocytes support complete myeloid and lymphoid differentiation from human CD34 cells. Br. J. Haematol. 127 (2004) 344-7.

[384] M. Gasparrini, D. Rivas, A. Elbaz, and G. Duque, Differential expression of cytokines in subcutaneous and marrow fat of aging C57BL/6J mice. Exp. Gerontol. 44 (2009) 613-8.

[385] W.P. Cawthorn, E.L. Scheller, B.S. Learman, S.D. Parlee, B.R. Simon, H. Mori, X. Ning, A.J. Bree, B. Schell, D.T. Broome, S.S. Soliman, J.L. DelProposto, C.N. Lumeng, A. Mitra, S.V. Pandit, K.A. Gallagher, J.D. Miller, V. Krishnan, S.K. Hui, M.A. Bredella, P.K. Fazeli, A. Klibanski, M.C. Horowitz, C.J. Rosen, and O.A. MacDougald, Bone Marrow Adipose Tissue Is an Endocrine Organ that Contributes to Increased Circulating Adiponectin during Caloric Restriction. Cell Metab. 20 (2014) 368-75.

[386] E.L. Scheller, A.A. Burr, O.A. MacDougald, and W.P. Cawthorn, Inside out: Bone marrow adipose tissue as a source of circulating adiponectin. Adipocyte 5 (2016) 251-269.

[387] J. Wang, G.L. Chen, S. Cao, M.C. Zhao, Y.Q. Liu, X.X. Chen, and C. Qian, Adipogenic niches for melanoma cell colonization and growth in bone marrow. Lab. Invest. 97 (2017) 737-745.

[388] H. Fairfield, C. Falank, M. Farrell, C. Vary, J.M. Boucher, H. Driscoll, L. Liaw, C.J. Rosen, and M.R. Reagan, Development of a 3D bone marrow adipose tissue model. Bone (2018).

[389] G. Luo, Y. He, and X. Yu, Bone Marrow Adipocyte: An Intimate Partner With Tumor Cells in Bone Metastasis. Front Endocrinol (Lausanne) 9 (2018) 339.

[390] G. Luo, L. Tian, J.M. Hock, and X. Yu, Editorial: Novel Endocrine Functions of Bone Marrow Fat. Front Endocrinol (Lausanne) 10 (2019) 349.

[391] H. Robles, S. Park, M.S. Joens, J.A.J. Fitzpatrick, C.S. Craft, and E.L. Scheller, Characterization of the bone marrow adipocyte niche with three-dimensional electron microscopy. Bone 118 (2019) 89-98.

[392] A. Samimi, M. Ghanavat, S. Shahrabi, S. Azizidoost, and N. Saki, Role of bone marrow adipocytes in leukemia and chemotherapy challenges. Cell. Mol. Life Sci. (2019).

[393] D. Ferland-McCollough, D. Maselli, G. Spinetti, M. Sambataro, N. Sullivan, A. Blom, and P. Madeddu, MCP-1 Feedback Loop Between Adipocytes and Mesenchymal Stromal Cells Causes Fat Accumulation and Contributes to Hematopoietic Stem Cell Rarefaction in the Bone Marrow of Patients With Diabetes. Diabetes 67 (2018) 1380-1394.

[394] M. Tavassoli, Cytochemistry of marrow and extramedullary adipocytes in monolayer cultures. Scand. J. Haematol. 20 (1978) 330-4.

[395] L.A. Johnson, B.E. Hoppel, E.L. Gerard, S.P. Miller, S.H. Doppelt, G.C. Zirzow, D.I. Rosenthal, J.M. Dambrosia, S.C. Hill, R.O. Brady, and et al., Quantitative chemical shift imaging of vertebral bone marrow in patients with Gaucher disease. Radiology 182 (1992) 451-5.

[396] A. Honda, T. Kurabayashi, T. Yahata, M. Tomita, H. Matsushita, K. Takakuwa, and K. Tanaka, Effects of pregnancy and lactation on trabecular bone and marrow adipocytes in rats. Calcif. Tissue Int. 67 (2000) 367-72.

[397] M.W. Hamrick, K.H. Ding, S. Ponnala, S.L. Ferrari, and C.M. Isales, Caloric restriction decreases cortical bone mass but spares trabecular bone in the mouse skeleton: implications for the regulation of bone mass by body weight. J. Bone Miner. Res. 23 (2008) 870-8.

[398] A. Krings, S. Rahman, S. Huang, Y. Lu, P.J. Czernik, and B. Lecka-Czernik, Bone marrow fat has brown adipose tissue characteristics, which are attenuated with aging and diabetes. Bone 50 (2012) 546-552.

[399] D. Seidlova-Wuttke, G. Stecher, M. Kammann, J. Haunschild, N. Eder, V. Stahnke, J. Wessels, and W. Wuttke, Osteoprotective effects of Cimicifuga racemosa and its triterpene-saponins are responsible for reduction of bone marrow fat. Phytomedicine : international journal of phytotherapy and phytopharmacology 19 (2012) 855-60.

[400] E.L. Scheller, N. Troiano, J.N. Vanhoutan, M.A. Bouxsein, J.A. Fretz, Y. Xi, T. Nelson, G. Katz, R. Berry, C.D. Church, C.R. Doucette, M.S. Rodeheffer, O.A. Macdougald, C.J. Rosen, and M.C. Horowitz, Use of osmium tetroxide staining with microcomputerized tomography to visualize and quantify bone marrow adipose tissue in vivo. Methods Enzymol. 537 (2014) 123-39.

[401] J.Z. Fan, Y. Wang, Y. Meng, G.W. Li, S.X. Chang, H. Nian, and Y.J. Liang, Panax notoginseng saponins mitigate ovariectomy-induced bone loss and inhibit marrow adiposity in rats. Menopause 22 (2015) 1343-50.

[402] S. Smaldone, N.P. Clayton, M. Del Solar, G. Pasqual-Gonzales, S.H. Cheng, B.M. Wentworth, M.B. Schaffler, and F. Ramirez, Fibrillin-1 Regulates Skeletal Stem Cell Differentiation by Modulating TGFbeta Activity Within the Marrow Niche. J. Bone Miner. Res. (2015).

[403] I.M. de Araujo, C.E. Salmon, A.K. Nahas, M.H. Nogueira-Barbosa, J. Elias Junior, and F.J. de Paula, Marrow Adipose Tissue Spectrum in Obesity and Type 2 Diabetes Mellitus. Eur. J. Endocrinol. (2016).

[404] F.C. Ko, J.S. Martins, P. Reddy, B. Bragdon, A.I. Hussein, L.C. Gerstenfeld, and M.B. Demay, Acute Phosphate Restriction Impairs Bone Formation and Increases Marrow Adipose Tissue in Growing Mice. J. Bone Miner. Res. 31 (2016) 2204-2214.

[405] G. Qiang, H. Whang Kong, S. Xu, H.A. Pham, S.D. Parlee, A.A. Burr, V. Gil, J. Pang, A. Hughes, X. Gu, G. Fantuzzi, O.A. MacDougald, and C.W. Liew, Lipodystrophy and severe metabolic dysfunction in mice with adipose tissue-specific insulin receptor ablation. Mol Metabol 5 (2016) 480-90.

[406] M. Styner, G.M. Pagnotti, C. McGrath, X. Wu, B. Sen, G. Uzer, Z. Xie, X. Zong, M.A. Styner, C.T. Rubin, and J. Rubin, Exercise Decreases Marrow Adipose Tissue Through ss-Oxidation in Obese Running Mice. J. Bone Miner. Res. 32 (2017) 1692-1702.

[407] F. Ermetici, S. Briganti, A. Delnevo, P. Cannao, G.D. Leo, S. Benedini, I. Terruzzi, F. Sardanelli, and L. Luzi, Bone marrow fat contributes to insulin sensitivity and adiponectin secretion in premenopausal women. Endocrine 59 (2018) 410-418.

[408] G. Li, Z. Xu, H. Lin, Y. Chen, X. Li, and S. Chang, Association between insulin resistance and the magnetic resonance spectroscopy-determined marrow fat fraction in nondiabetic postmenopausal women. Menopause 25 (2018) 676-682.

[409] W. Lu, Y. Wan, Z. Li, B. Zhu, C. Yin, H. Liu, S. Yang, Y. Zhai, Y. Yu, Y. Wei, and J. Shi, Growth differentiation factor 15 contributes to marrow adipocyte remodeling in response to the growth of leukemic cells. J. Exp. Clin. Cancer Res. 37 (2018) 66.

[410] D. Martel, B. Leporq, A. Saxena, H.M. Belmont, G. Turyan, S. Honig, R.R. Regatte, and G. Chang, 3T chemical shift-encoded MRI: Detection of altered proximal femur marrow adipose tissue composition in glucocorticoid users and validation with magnetic resonance spectroscopy. J. Magn. Reson. Imaging (2018).

[411] E.S.L.T. Parreiras, I.M. de Araujo, J. Elias, Jr., M.H. Nogueira-Barbosa, V.M.M. Suen, J.S. Marchini, J. Bonella, A.K. Nahas, C.E.G. Salmon, and F.J.A. de Paula, Short bowel syndrome: influence of nutritional therapy and incretin GLP1 on bone marrow adipose tissue. Ann. N. Y. Acad. Sci. 1415 (2018) 47-56.

[412] H. Artsi, I. Gurt, M. El-Haj, R. Müller, G.A. Kuhn, G. Ben Shalom, E. Cohen-Kfir, E. Abramowitz, L. Kandel, O. Safran, and R. Dresner-Pollak, Sirt1 Promotes a Thermogenic Gene Program in Bone Marrow Adipocytes: From Mice to (Wo)Men. Front Endocrinol (Lausanne) (2019).

[413] D. Giovannone, S. Paul, S. Schindler, C. Arata, D.T. Farmer, P. Patel, J. Smeeton, and J.G. Crump, Programmed conversion of hypertrophic chondrocytes into osteoblasts and marrow adipocytes within zebrafish bones. eLife 8 (2019).

[414] J. He, H. Fang, and X. Li, Vertebral bone marrow fat content in normal adults with varying bone densities at 3T magnetic resonance imaging. Acta Radiol. 60 (2019) 509-515.

[415] R.W. Anderson, S.L. Mann, D.A. Crouse, and J.G. Sharp, Modulation of one of three murine bone marrow stromal cell lines to adipose cells by serum and insulin. J. Supramol. Struct. Cell. Biochem. 16 (1981) 377-84.

[416] I. Touw, and B. Lowenberg, No stimulative effect of adipocytes on hematopoiesis in long-term human bone marrow cultures. Blood 61 (1983) 770-774.

[417] S. Zhou, K. Eid, and J. Glowacki, Cooperation between TGF-beta and Wnt pathways during chondrocyte and adipocyte differentiation of human marrow stromal cells. J. Bone Miner. Res. 19 (2004) 463-70.

[418] Y. Wang, Y. Li, K. Mao, J. Li, Q. Cui, and G.J. Wang, Alcohol-induced adipogenesis in bone and marrow: a possible mechanism for osteonecrosis. Clin Orthop Relat Res (2003) 213-24.

[419] A. Grey, V. Beckley, A. Doyle, S. Fenwick, A. Horne, G. Gamble, and M. Bolland, Pioglitazone increases bone marrow fat in type 2 diabetes: results from a randomized controlled trial. Eur. J. Endocrinol. 166 (2012) 1087-91.

[420] W. Shen, J. Chen, M. Gantz, M. Punyanitya, S.B. Heymsfield, D. Gallagher, J. Albu, E. Engelson, D. Kotler, X. Pi-Sunyer, and V. Gilsanz, MRI-measured pelvic bone marrow adipose tissue is inversely related to DXA-measured bone mineral in younger and older adults. Eur. J. Clin. Nutr. 66 (2012) 983-8.

[421] Y.H. Ma, A.V. Schwartz, S. Sigurdsson, T.F. Hue, T.F. Lang, T.B. Harris, C.J. Rosen, E. Vittinghoff, G. Eiriksdottir, A.M. Hauksdottir, K. Siggeirsdottir, G. Sigurdsson, D. Oskarsdottir, N. Napoli, L. Palermo, V. Gudnason, and X. Li, Circulating sclerostin associated with vertebral bone marrow fat in older men but not women. J. Clin. Endocrinol. Metab. 99 (2014) E2584-90.

[422] E.J. Limonard, A.G. Veldhuis-Vlug, L. van Dussen, J.H. Runge, M.W. Tanck, E. Endert, A.C. Heijboer, E. Fliers, C.E. Hollak, E.M. Akkerman, and P.H. Bisschop, Short-Term Effect of Estrogen on Human Bone Marrow Fat. J. Bone Miner. Res. 30 (2015) 2058-66.

[423] L.M. Pop, I. Lingvay, Q. Yuan, X. Li, B. Adams-Huet, and N.M. Maalouf, Impact of pioglitazone on bone mineral density and bone marrow fat content. Osteoporos. Int. 28 (2017) 3261-3269.

[424] B. de Almeida Mallmann, E.M. Martin, K. Soo Kim, N.L. Calderon-Apodaca, M.F.A. Baxter, J.D. Latorre, X. Hernandez-Velasco, L. Paasch-Martinez, C.M. Owens, S. Dridi, W.G. Bottje, E.S. Greene, and G. Tellez-Isaias, Evaluation of Bone Marrow Adipose Tissue and Bone Mineralization on Broiler Chickens Affected by Wooden Breast Myopathy. Front Physiol 10 (2019) 674.

[425] M. Tavassoli, Marrow adipose cells. Ultrastructural and histochemical characterization. Arch. Pathol. 98 (1974) 189-92.

[426] P.A. Bryon, O. Gentilhomme, and D. Fiere, [Histomorphometric analysis of bone-marrow adipose density and heterogeneity in myeloid aplasia and dysplasia (author's transl)]. Pathol. Biol. (Paris). 27 (1979) 209-13.

[427] M. Kashimura, Scanning electron microscopy studies of bone marrow. Scan. Electron Microsc. (1982) 445-53.

[428] M. Tavassoli, Marrow adipose cells and hemopoiesis: an interpretative review. Exp. Hematol. 12 (1984) 139-46.

[429] C. Rozman, E. Feliu, L. Berga, J.C. Reverter, C. Climent, and M.J. Ferran, Age-related variations of fat tissue fraction in normal human bone marrow depend both on size and number of adipocytes: a stereological study. Exp. Hematol. 17 (1989) 34-7.

[430] M.A. Tran, T.L. Dang, and M. Berlan, Effects of catecholamines on free fatty acid release from bone marrow adipose tissue. J. Lipid Res. 22 (1981) 1271-6.

[431] M.A. Tran, L.D. Tran, M. Berlan, and M. Lafontan, In vivo alpha 2-adrenoceptor-mediated inhibition of isoproterenol-stimulated free fatty acid output in dog bone marrow adipose tissue. Eur. J. Pharmacol. 76 (1981) 435-8.

[432] M.A. Tran, D.T. Lac, M. Berlan, and M. Lafontan, Interplay of alpha-2 and beta adrenoceptors in the control of free fatty acid release from bone marrow adipose tissue. J. Pharmacol. Exp. Ther. 230 (1984) 228-231.

[433] W. Shen, G. Velasquez, J. Chen, Y. Jin, S.B. Heymsfield, D. Gallagher, and F.X. Pi-Sunyer, Comparison of the relationship between bone marrow adipose tissue and volumetric bone mineral density in children and adults. Journal of clinical densitometry : the official journal of the International Society for Clinical Densitometry 17 (2014) 163-9.

[434] S.S. Shouse, S.L. Warren, and G.H. Whipple, II. Aplasia of marrow and fatal intoxication in dogs produced by roentgen radiation of all bones. The Journal of Experimental Medicine 53 (1931) 421-435.

[435] F.R. Sabin, F.R. Miller, K.C. Smithburn, R.M. Thomas, and L.E. Hummel, Changes in the bone marrow and blood cells of developing rabbits. The Journal of Experimental Medicine 64 (1936) 97-120.

[436] C.O. Warren, Tissue Metabolism Studies on Bone Marrow. Consideration in Relation to Tumor Metabolism. Cancer Res. 3 (1943) 621-625.

[437] B. Steinberg, Bone marrow regeneration in experimental benzene intoxication. Blood 4 (1949) 550-556.

[438] D.G. Scarpelli, Fat necrosis of bone marrow in acute pancreatitis. The American journal of pathology 32 (1956) 1077-1087.

[439] T. Nasu, Y. Tsukahara, and K. Terayama, A lipid metabolic disease —"membranous lipodystrophy"— an autopsy case demonstrating numerous peculiar membrane-structures composed of compound lipid in bone and bone marrow and various adipose tissues. Pathol. Int. 23 (1973) 539-558.

[440] R.J. Ferguson, R.H. Webber, and E.R. Hayes, Suppression and regeneration of rat bone marrow under the influence of methotrexate. An EM study. Acta Anat. (Basel). 92 (1975) 481-512.

[441] P.J. Cornbleet, R.C. Moir, and P.L. Wolf, A histochemical study of bone marrow hypoplasia in anorexia nervosa. Virchows Arch. A. Pathol. Anat. Histol. 374 (1977) 239-47.

[442] A. Biermann, and D. Graf von Keyserlingk, Ultrastructure of reticulum cells in the bone marrow. Acta Anat. (Basel). 100 (1978) 34-43.

[443] S. Bentley, and J. Foidart, Some properties of marrow derived adherent cells in tissue culture. Blood 56 (1980) 1006-1012.

[444] M.Y. Gordon, J.A. King, and E.C. Gordon-Smith, Bone marrow fibroblasts, fat cells and colony-stimulating activity. Br. J. Haematol. 46 (1980) 151-2.

[445] B. Wittels, Bone marrow biopsy changes following chemotherapy for acute leukemia. Am. J. Surg. Pathol. 4 (1980) 135-42.

[446] K. Harigaya, E.P. Cronkite, M.E. Miller, and R.K. Shadduck, Murine bone marrow cell line producing colony-stimulating factor. Proc. Natl. Acad. Sci. U. S. A. 78 (1981) 6963-6966.

[447] C.G. Potter, A.C. Rowell, and D.J. Weatherall, Continuous long-term culture of human bone marrow. Clin. Lab. Haematol. 3 (1981) 245-255.

[448] C. Rozman, L. Hernandez-Nieto, E. Montserrat, and R. Brugues, Prognostic significance of bone-marrow patterns in chronic lymphocytic leukaemia. Br. J. Haematol. 47 (1981) 529-37.

[449] M. Lanotte, D. Scott, T.M. Dexter, and T.D. Allen, Clonal preadipocyte cell lines with different phenotypes derived from murine marrow stroma: factors influencing growth and adipogenesis in vitro. J. Cell. Physiol. 111 (1982) 177-86.

[450] A. Islam, D. Catovsky, J.M. Goldman, and D.A.G. Galton, Histomorphological study of cellular interactions between stromal and haemopoietic stem cells in normal and leukaemic bone marrow. Histopathology 8 (1984) 293-313.

[451] F. Kuto, T. Nagaoka, Y. Watanabe, M. Hayashi, Y. Horasawa, Y. Hirasawa, and H. Tokuhiro, Chronic myelocytic leukemia: ultrastructural histopathology of bone marrow from patients in the chronic phase. Ultrastruct. Pathol. 6 (1984) 307-17.

[452] F. Malik, M.Y. Gordon, J.M. Goldman, and E.C. Gordon-Smith, Comparisons of the composition of fat cells obtained from the marrow of normal individuals or of subjects with aplastic anemia and from bone marrow cultures. Exp. Hematol. 12 (1984) 191-7.

[453] P. Bianco, M. Costantini, L.C. Dearden, and E. Bonucci, Alkaline phosphatase positive precursors of adipocytes in the human bone marrow. Br. J. Haematol. 68 (1988) 401-3.

[454] C. Schmid, B. Frisch, A. Beham, K. Jager, and G. Kettner, Comparison of bone marrow histology in early chronic granulocytic leukemia and in leukemoid reaction. Eur. J. Haematol. 44 (1990) 154-8.

[455] T. Yokota, C.S. Meka, K.L. Medina, H. Igarashi, P.C. Comp, M. Takahashi, M. Nishida, K. Oritani, J. Miyagawa, T. Funahashi, Y. Tomiyama, Y. Matsuzawa, and P.W. Kincade, Paracrine regulation of fat cell formation in bone marrow cultures via adiponectin and prostaglandins. J. Clin. Invest. 109 (2002) 1303-10.

[456] K. Kita, K. Kawai, and K. Hirohata, Changes in bone marrow blood flow with aging. J. Orthop. Res. 5 (1987) 569-75.

[457] S.K. Das, M.T. Scott, and P.K. Adhikary, Effect of the nature and amount of dietary energy on lipid composition of rat bone marrow. Lipids 10 (1975) 584-590.

[458] G.J. Wang, D.E. Sweet, S.I. Reger, and R.C. Thompson, Fat-cell changes as a mechanism of avascular necrosis of the femoral head in cortisone-treated rabbits. J. Bone Joint Surg. Am. 59 (1977) 729-35.

[459] I. Miyoshi, S. Irino, and K. Hiraki, Fibroblast-like transformation of human bone marrow fat cells in vitro. Exp. Cell Res. 41 (1966) 220-223.

[460] M. Shigematsu, H. Watanabe, and H. Sugihara, Proliferation and differentiation of unilocular fat cells in the bone marrow. Cell Struct. Funct. 24 (1999) 89-100.

[461] K. Miyanishi, T. Yamamoto, T. Irisa, A. Yamashita, S. Jingushi, Y. Noguchi, and Y. Iwamoto, Bone marrow fat cell enlargement and a rise in intraosseous pressure in steroid-treated rabbits with osteonecrosis. Bone 30 (2002) 185-90.

[462] T. Yokota, C.S. Meka, T. Kouro, K.L. Medina, H. Igarashi, M. Takahashi, K. Oritani, T. Funahashi, Y. Tomiyama, Y. Matsuzawa, and P.W. Kincade, Adiponectin, a fat cell product, influences the earliest lymphocyte precursors in bone marrow cultures by activation of the cyclooxygenase-prostaglandin pathway in stromal cells. J. Immunol. 171 (2003) 5091-9.

[463] G. Motomura, T. Yamamoto, K. Miyanishi, A. Yamashita, K. Sueishi, and Y. Iwamoto, Bone marrow fat-cell enlargement in early steroid-induced osteonecrosis--a histomorphometric study of autopsy cases. Pathol. Res. Pract. 200 (2005) 807-11.

[464] W.G. Hocking, and D.W. Golde, Long-term human bone marrow cultures. Blood 56 (1980) 118-24.

[465] I.A. Svet-Moldavskaya, S.N. Zinzar, G.J. Svet-Moldavsky, Z. Arlin, C. Vergara, B. Koziner, B.D. Clarkson, and J.F. Holland, Phenomenon of formation of giant fat-containing cells in human bone marrow cultures induced by human serum factor: normal and leukemic patterns. Proc. Natl. Acad. Sci. U. S. A. 80 (1983) 4847-4850.

[466] I.A. Svet-Moldavskaya, G.J. Svet-Moldavsky, S.N. Zinzar, J.F. Holland, C. Vergara, Z. Arlin, B. Koziner, and B.D. Clarkson, Induction of giant fat cells in human bone marrow culture by human serum factor. Biomedicine 35 (1981) 141-2.

[467] V. Sottile, K. Seuwen, and M. Kneissel, Enhanced marrow adipogenesis and bone resorption in estrogen-deprived rats treated with the PPARgamma agonist BRL49653 (rosiglitazone). Calcif. Tissue Int. 75 (2004) 329-37.

[468] R.B. Martin, and S.L. Zissimos, Relationships between marrow fat and bone turnover in ovariectomized and intact rats. Bone 12 (1991) 123-31.

[469] C. Wood, Membranous lipodystrophy of bone. Arch. Pathol. Lab. Med. 102 (1978) 22-7.

[470] E.L. Scheller, and C.J. Rosen, What's the matter with MAT? Marrow adipose tissue, metabolism, and skeletal health. Ann. N. Y. Acad. Sci. 1311 (2014) 14-30.

[471] M. Styner, W.R. Thompson, K. Galior, G. Uzer, X. Wu, S. Kadari, N. Case, Z. Xie, B. Sen, A. Romaine, G.M. Pagnotti, C.T. Rubin, M.A. Styner, M.C. Horowitz, and J. Rubin, Bone marrow fat accumulation accelerated by high fat diet is suppressed by exercise. Bone 64 (2014) 39-46.

[472] C.R. Doucette, M.C. Horowitz, R. Berry, O.A. MacDougald, R. Anunciado-Koza, R.A. Koza, and C.J. Rosen, A High Fat Diet Increases Bone Marrow Adipose Tissue (MAT) But Does Not Alter Trabecular or Cortical Bone Mass in C57BL/6J Mice. J. Cell. Physiol. 230 (2015) 2032-7.

[473] M. Styner, G.M. Pagnotti, K. Galior, X. Wu, W.R. Thompson, G. Uzer, B. Sen, Z. Xie, M.C. Horowitz, M.A. Styner, C. Rubin, and J. Rubin, Exercise Regulation of Marrow Fat in the Setting of PPARgamma Agonist Treatment in Female C57BL/6 Mice. Endocrinology 156 (2015) 2753-61.

[474] V. Singhal, K.K. Miller, M. Torriani, and M.A. Bredella, Short- and long-term reproducibility of marrow adipose tissue quantification by 1H-MR spectroscopy. Skeletal Radiol. 45 (2016) 221-5.

[475] M.A. Bredella, L.B. Greenblatt, A. Eajazi, M. Torriani, and E.W. Yu, Effects of Roux-en-Y gastric bypass and sleeve gastrectomy on bone mineral density and marrow adipose tissue. Bone 95 (2017) 85-90.

[476] E. Zach, and E. Shafrir, Composition of bone marrow adipose tissue in relation to body fat depots in various species. Isr. J. Med. Sci. 10 (1974) 1541-50.

[477] C. Rozman, J.C. Reverter, E. Feliu, M. Rozman, C. Climent, and L. Berga, [Adaptation of human bone marrow adipose tissue to different grades of hemopoietic cellularity]. Sangre (Barc). 38 (1993) 121-4.

[478] A. Garg, R.M. Peshock, and J.L. Fleckenstein, Adipose tissue distribution pattern in patients with familial partial lipodystrophy (Dunnigan variety). J. Clin. Endocrinol. Metab. 84 (1999) 170-4.

[479] L. Tornvig, L.I. Mosekilde, J. Justesen, E. Falk, and M. Kassem, Troglitazone treatment increases bone marrow adipose tissue volume but does not affect trabecular bone volume in mice. Calcif. Tissue Int. 69 (2001) 46-50.

[480] C.Y. Wang, Y.J. Hsu, Y.J. Peng, H.S. Lee, Y.C. Chang, C.S. Chang, S.W. Chiang, Y.C. Hsu, M.H. Lin, and G.S. Huang, Knee subchondral bone perfusion and its relationship to marrow fat and trabeculation on multi-parametric MRI and micro-CT in experimental CKD. Scientific reports 7 (2017) 3073.

[481] F.C. Schmeel, T. Vomweg, F. Traber, A. Gerhards, S.J. Enkirch, A. Faron, A.M. Sprinkart, L.C. Schmeel, J.A. Luetkens, D. Thomas, and G.M. Kukuk, Proton density fat fraction MRI

of vertebral bone marrow: Accuracy, repeatability, and reproducibility among readers, field strengths, and imaging platforms. J. Magn. Reson. Imaging (2019).

[482] W. Shen, J. Chen, M. Punyanitya, S. Shapses, S. Heshka, and S.B. Heymsfield, MRI-measured bone marrow adipose tissue is inversely related to DXA-measured bone mineral in Caucasian women. Osteoporos. Int. 18 (2007) 641-7.

[483] K. Casazza, L.J. Hanks, B. Hidalgo, H.H. Hu, and O. Affuso, Short-term physical activity intervention decreases femoral bone marrow adipose tissue in young children: a pilot study. Bone 50 (2012) 23-7.

[484] L. Soley, C. Falank, and M.R. Reagan, MicroRNA Transfer Between Bone Marrow Adipose and Multiple Myeloma Cells. Current osteoporosis reports 15 (2017) 162-170.

[485] I.K. Blom-Høgestøl, T. Mala, J.A. Kristinsson, E.-M. Hauge, C. Brunborg, H.L. Gulseth, and E.F. Eriksen, Changes in bone marrow adipose tissue one year after Roux-en-Y gastric bypass - a prospective cohort study. J. Bone Miner. Res. 0 (2019).

[486] A.L. Carvalho, B. Massaro, L. Silva, C.E.G. Salmon, S.Y. Fukada, M.H. Nogueira-Barbosa, J. Elias, Jr., M.C.F. Freitas, C.E.B. Couri, M.C. Oliveira, B.P. Simoes, C.J. Rosen, and F.J.A. de Paula, Emerging Aspects of the Body Composition, Bone Marrow Adipose Tissue and Skeletal Phenotypes in Type 1 Diabetes Mellitus. Journal of clinical densitometry : the official journal of the International Society for Clinical Densitometry 22 (2019) 420-428.

[487] P. Liu, Y. Ji, T. Yuen, E. Rendina-Ruedy, V.E. DeMambro, S. Dhawan, W. Abu-Amer, S. Izadmehr, B. Zhou, A.C. Shin, R. Latif, P. Thangeswaran, A. Gupta, J. Li, V. Shnayder, S.T. Robinson, Y.E. Yu, X. Zhang, F. Yang, P. Lu, Y. Zhou, L.L. Zhu, D.J. Oberlin, T.F. Davies, M.R. Reagan, A. Brown, T.R. Kumar, S. Epstein, J. Iqbal, N.G. Avadhani, M.I. New, H. Molina, J.B. van Klinken, E.X. Guo, C. Buettner, S. Haider, Z. Bian, L. Sun, C.J. Rosen, and M. Zaidi, Blocking FSH induces thermogenic adipose tissue and reduces body fat. Nature 546 (2017) 107-112.

[488] J.D. Evans, R.W. Riemenschneider, and S.F. Herb, Fat composition and in vitro oxygen consumption of marrow from fed and fasted rabbits. Arch. Biochem. Biophys. 53 (1954) 157-66.

[489] J.D. Evans, J.M. Baker, and M.J. Oppenheimer, Alteration of Rabbit Marrow Fat in Anemia From Acetylphenylhydrazine. American Journal of Physiology-Legacy Content 181 (1955) 504-508.

[490] J.D. Evans, Autoxidation of Normal Rabbit Marrow Fat in Acetate Buffer. American Journal of Physiology-Legacy Content 184 (1956) 461-464.

[491] P. Cohen, and F.H. Gardner, Effect of Massive Triamcinolone Administration in Blunting the Erythropoietic Response to Phenylhydrazine Hemolysis. J. Lab. Clin. Med. 65 (1965) 88-101.

[492] J.K. Gong, and J.S. Arnold, Skeletal marrow volume in dog. American Journal of Physiology-Legacy Content 209 (1965) 340-346.

[493] J.K. Gong, Effects of altitude acclimatization and deacclimatization on bone and marrow volume in dog. American Journal of Physiology-Legacy Content 209 (1965) 347-352.

[494] M. Tavassoli, D.T. Eastlund, L.T. Yam, R.S. Neiman, and H. Finkel, Gelatinous Transformation of Bone Marrow in Prolonged Self-Induced Starvation. Scand. J. Haematol. 16 (1976) 311-319.

[495] R.B. Mazess, Marrow fat and estimates of bone loss. Ann. Intern. Med. 102 (1985) 276-7.

[496] A.M. Laval-Jeantet, B. Roger, S. Bouysee, C. Bergot, and R.B. Mazess, Influence of vertebral fat content on quantitative CT density. Radiology 159 (1986) 463-6.

[497] W.T. Yuh, J.S. Collison, W.J. Sickels, T.J. Barloon, D.C. Brennan, and M.J. Flanigan, Partial lipodystrophy. Magnetic resonance findings in one case. J. Comput. Tomogr. 12 (1988) 287-91.

[498] D.I. Rosenthal, W. Mayo-Smith, M.M. Goodsitt, S. Doppelt, and H.J. Mankin, Bone and bone marrow changes in Gaucher disease: evaluation with quantitative CT. Radiology 170 (1989) 143-6.

[499] M. Lambert, C. Hubert, G. Depresseux, B. Vande Berg, J.P. Thissen, C. Nagant de Deuxchaisnes, and J.P. Devogelaer, Hematological changes in anorexia nervosa are correlated with total body fat mass depletion. Int. J. Eat. Disord. 21 (1997) 329-34.

[500] R.V. Mulkern, J. Huang, S. Vajapeyam, A.B. Packard, K. Oshio, and S. Grinspoon, Fat fractions and spectral T2 values in vertebral bone marrow in HIV- and non-HIV-infected men: a 1H spectroscopic imaging study. Magn. Reson. Med. 52 (2004) 552-8.

[501] J.F. Griffith, D.K. Yeung, G.E. Antonio, F.K. Lee, A.W. Hong, S.Y. Wong, E.M. Lau, and P.C. Leung, Vertebral bone mineral density, marrow perfusion, and fat content in healthy men and men with osteoporosis: dynamic contrast-enhanced MR imaging and MR spectroscopy. Radiology 236 (2005) 945-51.

[502] D.K. Yeung, J.F. Griffith, G.E. Antonio, F.K. Lee, J. Woo, and P.C. Leung, Osteoporosis is associated with increased marrow fat content and decreased marrow fat unsaturation: a proton MR spectroscopy study. J. Magn. Reson. Imaging 22 (2005) 279-85.

[503] J. Ren, I. Dimitrov, A.D. Sherry, and C.R. Malloy, Composition of adipose tissue and marrow fat in humans by 1H NMR at 7 Tesla. J. Lipid Res. 49 (2008) 2055-62.

[504] A.I. Idris, A. Sophocleous, E. Landao-Bassonga, M. Canals, G. Milligan, D. Baker, R.J. van't Hof, and S.H. Ralston, Cannabinoid receptor type 1 protects against age-related osteoporosis by regulating osteoblast and adipocyte differentiation in marrow stromal cells. Cell Metab. 10 (2009) 139-47.

[505] J.F. Griffith, D.K. Yeung, J.C. Leung, T.C. Kwok, and P.C. Leung, Prediction of bone loss in elderly female subjects by MR perfusion imaging and spectroscopy. Eur. Radiol. 21 (2011) 1160-9.

[506] X. Li, D. Kuo, A.L. Schafer, A. Porzig, T.M. Link, D. Black, and A.V. Schwartz, Quantification of vertebral bone marrow fat content using 3 Tesla MR spectroscopy: reproducibility, vertebral variation, and applications in osteoporosis. J. Magn. Reson. Imaging 33 (2011) 974-9.

[507] C. Vidal, S. Bermeo, W. Li, D. Huang, R. Kremer, and G. Duque, Interferon gamma inhibits adipogenesis in vitro and prevents marrow fat infiltration in oophorectomized mice. Stem Cells 30 (2012) 1042-8.

[508] M.A. Bredella, P.K. Fazeli, S.M. Daley, K.K. Miller, C.J. Rosen, A. Klibanski, and M. Torriani, Marrow fat composition in anorexia nervosa. Bone (2014).

[509] A.L. Schafer, X. Li, A.V. Schwartz, L.S. Tufts, A.L. Wheeler, C. Grunfeld, L. Stewart, S.J. Rogers, J.T. Carter, A.M. Posselt, D.M. Black, and D.M. Shoback, Changes in vertebral bone marrow fat and bone mass after gastric bypass surgery: A pilot study. Bone 74 (2015) 140-5.

[510] L. Singh, S. Tyagi, D. Myers, and G. Duque, Good, Bad, or Ugly: the Biological Roles of Bone Marrow Fat. Current osteoporosis reports 16 (2018) 130-137.

[511] Y. Zhang, Z. Zhou, C. Wang, X. Cheng, L. Wang, Y. Duanmu, C. Zhang, N. Veronese, and G. Guglielmi, Reliability of measuring the fat content of the lumbar vertebral marrow and paraspinal muscles using MRI mDIXON-Quant sequence. Diagnostic and interventional radiology (Ankara, Turkey) 24 (2018) 302-307.

[512] D.L. Gursahaney, M.K. Jesse, and J. Stoneback, Extraosseous marrow fat: an MRI sign of acute aggressive osteomyelitis. BJR case reports 5 (2019) 20180050.

[513] M. Schoonderwoerd, C.E. Doige, G.A. Wobeser, and J.M. Naylor, Protein energy malnutrition and fat mobilization in neonatal calves. Can. Vet. J. 27 (1986) 365-71.

[514] D. Schellinger, C.S. Lin, J. Lim, H.G. Hatipoglu, J.C. Pezzullo, and A.J. Singer, Bone marrow fat and bone mineral density on proton MR spectroscopy and dual-energy X-ray absorptiometry: their ratio as a new indicator of bone weakening. AJR. Am. J. Roentgenol. 183 (2004) 1761-5.

[515] A. Elbaz, X. Wu, D. Rivas, J.M. Gimble, and G. Duque, Inhibition of fatty acid biosynthesis prevents adipocyte lipotoxicity on human osteoblasts in vitro. Journal of cellular and molecular medicine 14 (2010) 982-91.

[516] M.A. Bredella, M. Torriani, R.H. Ghomi, B.J. Thomas, D.J. Brick, A.V. Gerweck, C.J. Rosen, A. Klibanski, and K.K. Miller, Vertebral Bone Marrow Fat Is Positively Associated With Visceral Fat and Inversely Associated With IGF-1 in Obese Women. Obesity (2011).

[517] G. Gokalp, F.S. Mutlu, Z. Yazici, and N. Yildirim, Evaluation of vertebral bone marrow fat content by chemical-shift MRI in osteoporosis. Skeletal Radiol. 40 (2011) 577-85.

[518] A. Regis-Arnaud, B. Guiu, P.M. Walker, D. Krause, F. Ricolfi, and D. Ben Salem, Bone marrow fat quantification of osteoporotic vertebral compression fractures: comparison of multi-voxel proton MR spectroscopy and chemical-shift gradient-echo MR imaging. Acta Radiol. 52 (2011) 1032-6.

[519] H. Zhang, W. Lu, Y. Zhao, P. Rong, R. Cao, W. Gu, J. Xiao, D. Miao, J. Lappe, R. Recker, and G.G. Xiao, Adipocytes derived from human bone marrow mesenchymal stem cells exert inhibitory effects on osteoblastogenesis. Current molecular medicine 11 (2011) 489-502.

[520] M.A. Bredella, E. Lin, A.V. Gerweck, M.G. Landa, B.J. Thomas, M. Torriani, M.L. Bouxsein, and K.K. Miller, Determinants of bone microarchitecture and mechanical properties in obese men. J. Clin. Endocrinol. Metab. 97 (2012) 4115-22.

[521] J.M. Patsch, X. Li, T. Baum, S.P. Yap, D.C. Karampinos, A.V. Schwartz, and T.M. Link, Bone marrow fat composition as a novel imaging biomarker in postmenopausal women with prevalent fragility fractures. J. Bone Miner. Res. 28 (2013) 1721-8.

[522] F. Wang, S.E. Mullican, J.R. DiSpirito, L.C. Peed, and M.A. Lazar, Lipoatrophy and severe metabolic disturbance in mice with fat-specific deletion of PPARgamma. Proc. Natl. Acad. Sci. U. S. A. 110 (2013) 18656-61.

[523] R.N. Moorthi, W. Fadel, G.J. Eckert, K. Ponsler-Sipes, S.M. Moe, and C. Lin, Bone marrow fat is increased in chronic kidney disease by magnetic resonance spectroscopy. Osteoporos. Int. 26 (2015) 1801-7.

[524] B.S. Koo, Y. Song, J.H. Shin, S. Lee, and T.H. Kim, Evaluation of disease chronicity by bone marrow fat fraction using sacroiliac joint magnetic resonance imaging in patients with spondyloarthritis: A retrospective study. International journal of rheumatic diseases 22 (2019) 734-741.

[525] D. Sicard, N. Casadevall, B. Wyplosz, F. Picart, and P. Blanene, Anorexia nervosa and gelatinous transformation of bone marrow. Nouv. Rev. Fr. Hematol. 36 Suppl 1 (1994) S85-6.

[526] D.D. Waid, and R.J. Warren, Seasonal variations in physiological indices of adult female white-tailed deer in Texas. J. Wildl. Dis. 20 (1984) 212-9.

[527] C.C. Gluer, and H.K. Genant, Impact of marrow fat on accuracy of quantitative CT. J. Comput. Assist. Tomogr. 13 (1989) 1023-35.

[528] J.S. Huang, R.V. Mulkern, and S. Grinspoon, Reduced intravertebral bone marrow fat in HIV-infected men. AIDS 16 (2002) 1265-9.

[529] T. Baum, S.P. Yap, D.C. Karampinos, L. Nardo, D. Kuo, A.J. Burghardt, U.B. Masharani, A.V. Schwartz, X. Li, and T.M. Link, Does vertebral bone marrow fat content correlate with abdominal adipose tissue, lumbar spine bone mineral density, and blood biomarkers in women with type 2 diabetes mellitus? J. Magn. Reson. Imaging 35 (2012) 117-24.

[530] C.K. Chan, E.Y. Seo, J.Y. Chen, D. Lo, A. McArdle, R. Sinha, R. Tevlin, J. Seita, J. Vincent-Tompkins, T. Wearda, W.J. Lu, K. Senarath-Yapa, M.T. Chung, O. Marecic, M. Tran, K.S. Yan, R. Upton, G.G. Walmsley, A.S. Lee, D. Sahoo, C.J. Kuo, I.L. Weissman, and M.T. Longaker, Identification and specification of the mouse skeletal stem cell. Cell 160 (2015) 285-98.

[531] P. Robey, "Mesenchymal stem cells": fact or fiction, and implications in their therapeutic use. F1000Research 6 (2017).

[532] C.K.F. Chan, G.S. Gulati, R. Sinha, J.V. Tompkins, M. Lopez, A.C. Carter, R.C. Ransom, A. Reinisch, T. Wearda, M. Murphy, R.E. Brewer, L.S. Koepke, O. Marecic, A. Manjunath, E.Y. Seo, T. Leavitt, W.J. Lu, A. Nguyen, S.D. Conley, A. Salhotra, T.H. Ambrosi, M.R. Borrelli, T. Siebel, K. Chan, K. Schallmoser, J. Seita, D. Sahoo, H. Goodnough, J. Bishop, M. Gardner, R. Majeti, D.C. Wan, S. Goodman, I.L. Weissman, H.Y. Chang, and M.T. Longaker, Identification of the Human Skeletal Stem Cell. Cell 175 (2018) 43-56.e21.

[533] B.M. Abdallah, C.H. Jensen, G. Gutierrez, R.G. Leslie, T.G. Jensen, and M. Kassem, Regulation of human skeletal stem cells differentiation by Dlk1/Pref-1. J. Bone Miner. Res. 19 (2004) 841-52.

[534] S. Raeth, B. Sacchetti, G. Siegel, U.A. Mau-Holzmann, J. Hansmann, G. Vacun, T.G. Hauk, K. Pfizenmaier, and A. Hausser, A mouse bone marrow stromal cell line with skeletal stem cell characteristics to study osteogenesis in vitro and in vivo. Stem cells and development 23 (2014) 1097-108.

[535] D.L. Worthley, M. Churchill, J.T. Compton, Y. Tailor, M. Rao, Y. Si, D. Levin, M.G. Schwartz, A. Uygur, Y. Hayakawa, S. Gross, B.W. Renz, W. Setlik, A.N. Martinez, X. Chen, S. Nizami, H.G. Lee, H.P. Kang, J.M. Caldwell, S. Asfaha, C.B. Westphalen, T. Graham, G. Jin, K. Nagar, H. Wang, M.A. Kheirbek, A. Kolhe, J. Carpenter, M. Glaire, A. Nair, S. Renders, N. Manieri, S. Muthupalani, J.G. Fox, M. Reichert, A.S. Giraud, R.F. Schwabe, J.P. Pradere, K. Walton, A. Prakash, D. Gumucio, A.K. Rustgi, T.S. Stappenbeck, R.A. Friedman, M.D. Gershon, P. Sims, T. Grikscheit, F.Y. Lee, G. Karsenty, S. Mukherjee, and T.C. Wang,

Gremlin 1 identifies a skeletal stem cell with bone, cartilage, and reticular stromal potential. Cell 160 (2015) 269-84.

[536] R.H. Lee, B. Kim, I. Choi, H. Kim, H.S. Choi, K. Suh, Y.C. Bae, and J.S. Jung, Characterization and expression analysis of mesenchymal stem cells from human bone marrow and adipose tissue. Cell. Physiol. Biochem. 14 (2004) 311-24.

[537] E.L. Herzog, L. Chai, and D.S. Krause, Plasticity of marrow-derived stem cells. Blood 102 (2003) 3483-93.

[538] J.M. Gimble, C.E. Robinson, X. Wu, K.A. Kelly, B.R. Rodriguez, S.A. Kliewer, J.M. Lehmann, and D.C. Morris, Peroxisome proliferator-activated receptor-gamma activation by thiazolidinediones induces adipogenesis in bone marrow stromal cells. Mol. Pharmacol. 50 (1996) 1087-94.

[539] A. Cohen, D.W. Dempster, R.R. Recker, J.M. Lappe, H. Zhou, A. Zwahlen, R. Muller, B. Zhao, X. Guo, T. Lang, I. Saeed, X.S. Liu, X.E. Guo, S. Cremers, C.J. Rosen, E.M. Stein, T.L. Nickolas, D.J. McMahon, P. Young, and E. Shane, Abdominal fat is associated with lower bone formation and inferior bone quality in healthy premenopausal women: a transiliac bone biopsy study. J. Clin. Endocrinol. Metab. 98 (2013) 2562-72.

[540] D.W. Dempster, J.E. Compston, M.K. Drezner, F.H. Glorieux, J.A. Kanis, H. Malluche, P.J. Meunier, S.M. Ott, R.R. Recker, and A.M. Parfitt, Standardized nomenclature, symbols, and units for bone histomorphometry: a 2012 update of the report of the ASBMR Histomorphometry Nomenclature Committee. J. Bone Miner. Res. 28 (2013) 2-17.

[541] S. Verma, J.H. Rajaratnam, J. Denton, J.A. Hoyland, and R.J. Byers, Adipocytic proportion of bone marrow is inversely related to bone formation in osteoporosis. J. Clin. Pathol. 55 (2002) 693-8.

[542] K. Arashi, Quantitative analysis of biopsied bone marrow tissue embedded in resin from hemopathic patients. I. Distribution of marrow adipose volume (MAV) and hematopoietic cells (HC) in certain part of the bone marrow biopsied specimen. Nihon Ketsueki Gakkai zasshi : journal of Japan Haematological Society 46 (1983) 65-80.

[543] L. Berman, and A.R. Axelrod, Fat, total cell and megakaryocyte content of sections of aspirated marrow of normal persons. Am. J. Clin. Pathol. 20 (1950) 686-7.

[544] J.A. Wright, A comparison of rat femoral, sternebral and lumbar vertebral bone marrow fat content by subjective assessment and image analysis of histological sections. J. Comp. Pathol. 100 (1989) 419-426.

[545] Y.X. Wang, J.F. Griffith, M. Deng, D.K. Yeung, and J. Yuan, Rapid increase in marrow fat content and decrease in marrow perfusion in lumbar vertebra following bilateral oophorectomy: an MR imaging-based prospective longitudinal study. Korean journal of radiology 16 (2015) 154-9.

[546] J.F. Griffith, D.K. Yeung, S.K. Chow, J.C. Leung, and P.C. Leung, Reproducibility of MR perfusion and (1)H spectroscopy of bone marrow. J. Magn. Reson. Imaging 29 (2009) 1438-42.

[547] F. Geiser, P. Murtz, G. Lutterbey, F. Traber, W. Block, K. Imbierowicz, G. Schilling, H. Schild, and R. Liedtke, Magnetic resonance spectroscopic and relaxometric determination of bone marrow changes in anorexia nervosa. Psychosom. Med. 63 (2001) 631-7.

[548] I.S. Idilman, F. Gumruk, M. Haliloglu, and M. Karcaaltincaba, The Feasibility of Magnetic Resonance Imaging for Quantification of Liver, Pancreas, Spleen, Vertebral Bone Marrow, and Renal Cortex R2* and Proton Density Fat Fraction in Transfusion-Related Iron Overload. Turkish journal of haematology : official journal of Turkish Society of Haematology 33 (2016) 21-7.

[549] L.J. Vogt, A. Steveling, P.J. Meffert, M.L. Kromrey, R. Kessler, N. Hosten, J. Kruger, S. Gartner, A.A. Aghdassi, J. Mayerle, M.M. Lerch, and J.P. Kuhn, Magnetic Resonance Imaging of Changes in Abdominal Compartments in Obese Diabetics during a Low-Calorie Weight-Loss Program. PLoS One 11 (2016) e0153595.

[550] D. Schellinger, C.S. Lin, D. Fertikh, J.S. Lee, W.C. Lauerman, F. Henderson, and B. Davis, Normal lumbar vertebrae: anatomic, age, and sex variance in subjects at proton MR spectroscopy--initial experience. Radiology 215 (2000) 910-6.

[551] T. Baum, S.P. Yap, D.C. Karampinos, L. Nardo, D. Kuo, A.J. Burghardt, U.B. Masharani, A.V. Schwartz, X. Li, and T.M. Link, Does vertebral bone marrow fat content correlate with abdominal adipose tissue, lumbar spine bone mineral density, and blood biomarkers in women with type 2 diabetes mellitus? Journal of magnetic resonance imaging : JMRI 35 (2012) 117-24.

[552] J.F. Griffith, D.K. Yeung, G.E. Antonio, S.Y. Wong, T.C. Kwok, J. Woo, and P.C. Leung, Vertebral marrow fat content and diffusion and perfusion indexes in women with varying bone density: MR evaluation. Radiology 241 (2006) 831-8.

[553] M.A. Bredella, A.V. Gerweck, L.A. Barber, A. Breggia, C.J. Rosen, M. Torriani, and K.K. Miller, Effects of growth hormone administration for 6 months on bone turnover and bone marrow fat in obese premenopausal women. Bone 62 (2014) 29-35.

[554] G.M. Blake, J.F. Griffith, D.K. Yeung, P.C. Leung, and I. Fogelman, Effect of increasing vertebral marrow fat content on BMD measurement, T-Score status and fracture risk prediction by DXA. Bone 44 (2009) 495-501.

[555] P.J. Littrup, A.M. Aisen, E.M. Braunstein, and W. Martel, Magnetic resonance imaging of femoral head development in roentgenographically normal patients. Skeletal Radiol. 14 (1985) 159-63.

[556] P.K. Fazeli, A. Faje, M.A. Bredella, S. Polineni, S. Russell, M. Resulaj, C.J. Rosen, and A. Klibanski, Changes in marrow adipose tissue with short-term changes in weight in premenopausal women with anorexia nervosa. Eur. J. Endocrinol. (2018).

[557] H.U. Kauczor, B. Dieti, G. Brix, K. Jarosch, M.V. Knopp, and G. van Kaick, Fatty replacement of bone marrow after radiation therapy for Hodgkin disease: quantification with chemical shift imaging. J. Magn. Reson. Imaging 3 (1993) 575-80.

[558] M.M. Goodsitt, and D.I. Rosenthal, Quantitative computed tomography scanning for measurement of bone and bone marrow fat content. A comparison of single- and dual-energy techniques using a solid synthetic phantom. Invest. Radiol. 22 (1987) 799-810.

[559] L. Arentsen, K.E. Hansen, M. Yagi, Y. Takahashi, R. Shanley, A. McArthur, P. Bolan, T. Magome, D. Yee, J. Froelich, and S.K. Hui, Use of dual-energy computed tomography to measure skeletal-wide marrow composition and cancellous bone mineral density. J. Bone Miner. Metab. 35 (2017) 428-436.

[560] G. Kerckhofs, S. Stegen, N. van Gastel, A. Sap, G. Falgayrac, G. Penel, M. Durand, F.P. Luyten, L. Geris, K. Vandamme, T. Parac-Vogt, and G. Carmeliet, Simultaneous three-dimensional visualization of mineralized and soft skeletal tissues by a novel microCT contrast agent with polyoxometalate structure. Biomaterials 159 (2018) 1-12.
